# Modeling combinatorial regulation from single-cell multi-omics provides regulatory units underpinning cell type landscape using cRegulon

**DOI:** 10.1186/s13059-025-03680-w

**Published:** 2025-07-24

**Authors:** Zhanying Feng, Xi Chen, Zhana Duren, Jingxue Xin, Hao Miao, Qiuyue Yuan, Yong Wang, Wing Hung Wong

**Affiliations:** 1 State Key Laboratory of Mathematical Sciences, Academy of Mathematics and Systems Science, Chinese Academy of Sciences, Beijing, 100190 China; 2 Department of Statistics, Department of Biomedical Data Science, Bio-X Program, Stanford University, Stanford, CA 94305 USA; 3 Center for Computational Biology and Bioinformatics and Department of Medical and Molecular Genetics, Indiana University School of Medicine, Indianapolis, IN 46202 USA; 4 Center for Human Genetics and Department of Genetics and Biochemistry, Clemson University, Greenwood, SC 29646 USA; 5 School of Mathematics, University of Chinese Academy of Sciences, Chinese Academy of Sciences, Beijing, 100049 China; 6 Center for Excellence in Animal Evolution and Genetics, Chinese Academy of Sciences, Kunming, 650223 China; 7 Key Laboratory of Systems Biology, Hangzhou Institute for Advanced Study, University of Chinese Academy of Sciences, Chinese Academy of Sciences, Hangzhou, 330106 China

## Abstract

Advances in single-cell technology enable large-scale generation of omics data, promising for clarifying gene regulatory networks governing different cell type/states. Nonetheless, prevailing methods fail to account for universal and reusable regulatory modules in GRNs, which are fundamental underpinnings of cell type landscape. We introduce cRegulon to infer regulatory modules by modeling combinatorial regulation of transcription factors based on diverse GRNs from single-cell multi-omics data. Through benchmarking and applications using simulated datasets and real datasets, cRegulon outperforms existing approaches in identifying TF combinatorial modules as regulatory units and annotating cell types. cRegulon offers new insights and methodology into combinatorial regulation.

**Supplementary Information:**

The online version contains supplementary material available at 10.1186/s13059-025-03680-w.

## Background

Cells are the fundamental units of living organisms. They can be grouped into different cell types or cellular states based on the cells’ morphologies, internal compositions, and intracellular biological processes [1]. Categorization and organization of cells into types greatly reduces the complexity of investigating the organization and function of cells and enables higher resolution study of human health and disease [2]. A wide range of cellular properties has been measured to determine cell types [3–5], but there has not been a consistent and standard definition of cell types [1]. This problem is hindered by limitation of high-resolution data resources and systematic methodologies for modeling fundamental units. Promisingly, some recent progress, including both technologies and theories, can help us move forward in understanding cellular states.

First, in the past 15 years, single-cell technology has witnessed remarkable progress, revolutionizing our understanding of cellular heterogeneity and complexity. With increasing cell number (such as human cell atlas [6–8] and mouse cell atlas [9]), expanding omics diversity (such as transcriptomics, proteomics, metabolomics, epigenomics, spatial-omics, and genomics), and decreasing cost, advances in single-cell technology have propelled discoveries in diverse areas including developmental biology [10, 11], cancer research [12], immunology [13], and neuroscience [14]. These efforts to analyze and characterize individual cells have resulted in a large amount of single-cell data informative on previously inaccessible cell types. For example, in the immune system alone, human cell atlas has identified 101 cell types and studied their distribution in 16 different tissues [13]. The increasing amount of single-cell data provides a foundation for characterizing cell type landscape in a systematic and global viewpoint.

Second, given single-cell data, cell types have been identified as clusters of similar cells, delineated by distinctive features such as gene expression profiles, epigenetic alterations, and molecular signatures. This offers the possibility of understand cell types under the framework of Waddington’s landscape [15]. In this framework, one imagines a cell as a pebble going downhill along established pathways in the terrain, guided by forces that direct it toward various potential outcomes depicted as basins. Waddington’s visionary insights also hinted at the existence of genetic regulatory mechanisms underlying the structure of this landscape. His concept of epigenetic landscape along with its underlying regulatory mechanisms has gained attention from numerous researchers [15–21], leading to further endeavors [22–24] aimed at formalizing the Waddington landscape in dynamic systems where cell types are perceived as attractors, fundamentally characterized by its underlying gene regulatory network (GRN) [15, 17] composing of transcription factors (TFs), regulatory elements (REs), and target genes (TGs). The GRN serves as a pivotal tool, elucidating the establishment, perturbation, and transition dynamics of cell types [25]. The GRN underlying Waddington landscape has been investigated and quantified in low dimension, both experimentally [26] and computationally [27–32]. However, genome-scale analysis has been lacking due to the difficulty in analyzing GRN’s which may involve tens of thousands of genes and hundreds of thousands of regulatory elements.

**Fig. 1:**
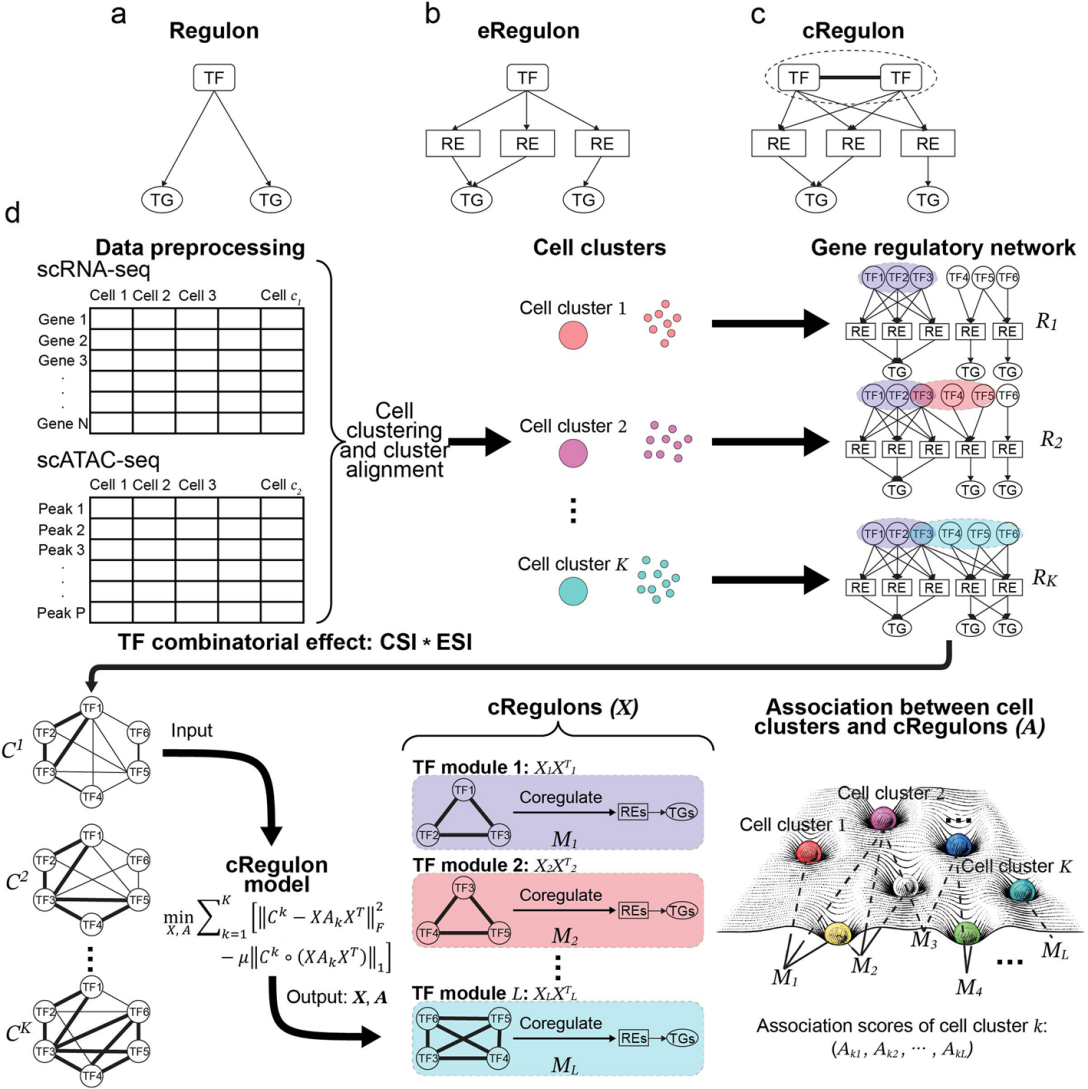
The conceptual representation of **a** Regulon, **b** eRegulon, and **c** cRegulon. **d** The schematic of cRegulon model. scRNA-seq and scATAC-seq data will undergo preprocessing, cell clustering, and GRN construction for each cell cluster. Then we combine connection specificity index (CSI) and expression specificity index (ESI) to define TF’s combinatorial effect from GRN. The TF combinatorial effect matrices of all cell clusters are input into an optimization model to identify TF modules, whose specific REs and TGs will form cRegulons. Simultaneously, our model will output association scores between cRegulons and cell clusters, underpinning cell type landscape by the usage of universal and reusable cRegulons for each cell cluster

Fortunately, one can reduce the complexity in the analysis of GRNs by taking advantage of their modularity [33, 34]. GRNs are believed to be modular in the sense that each GRN can be decomposed into several regulatory modules, each representing a cohesive functional unit consisting of a set of regulators that act on a set of REs to orchestrate synchronized gene expression necessary for specific biological functions or pathways [35–37]. These regulators, represented by TFs, may offer a more sensible choice of coordinates to define cell types [38]. Traditionally, the simplest definition of a regulator unit is a Regulon (Fig. 1a), which represents a collection of TGs under the control of one specific TF. Regulon can be inferred from diverse types of TF binding location, genome-wide perturbation, and gene expression data [35, 39–44]. Regulons have been extensively employed to delineate heterogeneous regulatory mechanisms across various cell types, including those pertinent to disease [45–47] and cancer development [48]. With the rapid expansion of genome-wide chromatin state datasets, analysis of gene regulation has expanded to encompass REs such as enhancers and insulators, denoting the binding sites of TFs orchestrating the expression of nearby TGs. This gives rise to the conceptualization of a regulatory unit known as the enhancer-regulon (eRegulon), comprising a TF, its associated binding REs, and TGs (Fig. 1b). eRegulon can be inferred from multi-omics data (gene expression and chromatin accessibility) by tools such as ChromVAR [49], i-cisTarget [50], PECA and PECA2 [51, 52], and SCENIC + [53], and has been applied to identify the master regulators in stem cell differentiation [54], to interpret conserved regions for the non-model organisms [55], and to explain how genetic variants affect complex traits through gene regulation in certain tissues or cell types [56–59]. Nonetheless, the units of Regulon and eRegulon only consider single TF and neither of them can account for the combinatorial nature of gene regulation involving multiple TFs. In many experiments on cell differentiation, cell reprogramming, and cell transdifferentiation, it is observed that several TFs could work together to regulate common TGs in a collaborative or competing manner, characterizing cell fate and developmental decisions [60]. For example, *Sox2*, *Nanog*, and *Pou5f1* cooperatively govern pluripotency in mESC [61, 62] (Additional file 1: Fig. S1). *MYOD*, *MYOG*, *MEF2A*, and *MYF6* collectively dictate the specification of skeletal muscle [63–65]. Perturbing a combination of key TFs is necessary to reprogram the cell fate from fibroblast to iPSC (*Oct3/4*, *Sox2*, *c-Myc*, and *Klf4*) [61], cardiomyocytes (*Gata4*, *Mef2c*, and *Tbx5*) [66], neurons (*Ascl1*, *Brn2*, and *Myt1l*) [67], and hepatocytes (*Gata4*, *Hnf1α*, and *Foxa3*) [68].

Despite the promise of modeling regulatory units by TF combinatorial regulation, there are only a few works in this field, and they are limited in their ability to account for cell-type specificity in the regulation. For example, some databases profile TF interactions such as protein interactions [69] and motif similarity [70], but they are not context-specific. There are a limited number of studies that systematically model and infer combinatorial regulation, and these studies are usually based on bulk data. For example, some methods rely on TF ChIP-seq to identify TF combinations [71–77]. However, TF ChIP-seq data is difficult to profile at the cell type level. On the other hand, Hi-C data provides another evidence of TF binding regions that are connected by chromatin loops, and there are also some existing methods based on Hi-C data [78, 79]. Nevertheless, like ChIP-seq, the limitation of Hi-C data is the requirement for the large number of cells for sequencing, which makes it impossible to be applied to cell type analysis. The advance in single-cell genomic sequencing promises analysis at the cell type level. By combining scRNA-seq data with co-expression or covariation analysis, PPI, or motif similarity, the TF combination can be inferred at the cell type level [80, 81]. However, such current methods lack the ability to combine diverse cell types to infer the regulatory units in the modular GRNs underlying Waddington’s landscape. Therefore, methods for the inference of combinatorial regulatory modules based on single-cell multi-omics data are urgently needed.

The foregoing discussions underscore two important points: (1) single-cell multi-omics offer a broader spectrum of cell type resource useful for the systematic study of GRNs underlying cell types; (2) there is a need for new methods to infer TF combinatorial modules, which are interpretable functional units in these GRNs. In this paper, we refer to such a TF combinatorial module as a cRegulon. Specifically, a cRegulon (Fig. 1c) is a set of TF pairs (TF module), a corresponding set of binding REs, and a set of TGs whose expressions are co-regulated by these TFs and REs. The GRN of a cell type may be composed of several distinct cRegulons that regulate gene expression in distinct pathways or processes. Conversely, a cRegulon may be shared by GRNs of multiple cell types.

The identification of the TF modules is a key step to the construction of cRegulon. Here, we present a method to simultaneously infer TF modules and annotate cell types based on GRN from scRNA-seq and scATAC-seq data. The scRNA-seq and scATAC-seq data can be paired at either the cell level or the context level. In this method, single-cell multi-omics data first undergoes initial data preprocessing, cell clustering, and GRN construction for each cell cluster. For each cluster-specific GRN, we combine the co-regulation effect and activity specificities to define a combinatorial effect for the TF pair. And in this way, we obtain a matrix $C$ containing such pairwise combinatorial effects. To model modularity of the GRN, we assume $C$ is well approximated by a mixture of rank-1 matrices and each of them corresponds to a module of co-regulating TFs. We design an optimization model (Eq. 2 below) to identify the TF modules across all cell clusters. The model’s outputs are a set of cRegulons, each represented by their TF module, REs, and TGs. Simultaneously, we also annotate each cell type (i.e., biologically well annotated cell cluster) by the cRegulons. It is important to note that one cell type can utilize multiple cRegulons and conversely, one cRegulon may be relevant to multiple cell types. To validate our cRegulon concept, we apply our method to an in-silico data simulation, which proves cRegulon’s superiority in identifying TF modules and cell type compositions over existing methods. Through a real data simulation by mixing four cell lines, the biological properties of cRegulon-identified regulatory units are validated: (1) cRegulon can precisely capture the hallmark TFs distinctive of cell types; (2) cRegulon can identify TF modules having higher functional concentration; (3) TFs in cRegulons have superior capability in forming TF combinations compared to other TF-centric methods, as judged by ground-truths defined by independent ChIP-seq, ChIA-PET, and Protein–Protein Interaction (PPI) data. Extending our approach to large-scale single-cell data, we apply our method to 54 cell types from a human fetal atlas. The results show that our approach can identify regulatory units underpinning early human development, characterize diverse fetal cell types, reveal spatial and temporal cellular states, and offer useful annotation of beta cell subpopulations in an external single-cell data. Finally, to explore cell state transition, we generate time series scRNA-seq and scATAC-seq data during RA-induced mEB differentiation from day 0 to day 10 in-vitro. The results demonstrate cRegulon’s ability to identify TF modules governing cell state transitions and to reveal intricate details of TF combinatorial regulation. Using a mouse fetal brain atlas, we show the temporal patterns of these regulatory units, which are inferred from the in-vitro time course data, aligned with developmental changes in-vivo. In conclusion, cRegulon is a promising method to model combinatorial regulations into regulatory underpinnings of cell type landscape.

## Results

### cRegulon models TF’s combinatorial regulation from scRNA-seq and scATAC-seq data

cRegulon is introduced as a concept to integrate gene expression and epigenome state into regulatory units of gene regulation underlying cell types. It is formally defined as the TF combinatorial module as well as the RE that they bind to and the TGs that they regulate. We developed an optimization model to simultaneously infer cRegulons and their associations with cell types (depicted in Fig. 1d).

The initial step involves data preprocessing, followed by clustering the single-cell data into *K* cell clusters. For single-cell data that are not paired at the cell level, cell clusters from the two data modalities should be aligned first, so that we can obtain the pseudo-bulk RNA-profile and the ATAC profile for each aligned cell cluster. This can be achieved by integrative clustering tools like Seurat [82] and CoupledNMF [83]. Subsequently, in tandem with the paired pseudo-bulk RNA-seq and ATAC-seq data of each cell cluster, we construct its regulatory network using specialized GRN construction tools, such as PECA2 [52]. The cluster-specific regulatory networks are encapsulated in the TF-TG regulatory strength matrices ${R}^{1}$,${R}^{2}$,$\cdots$, ${R}^{K}$, where $K$ denotes that number of cell clusters.

GRNs are believed to be modular [33, 34]. We assume that the modularity of a GRN is induced by TF combinatorial modules, which can be shared by GRNs of many cell clusters or specific to certain cell clusters. Therefore, we conduct a joint analysis of the GRNs from all the cell clusters to infer TF combinatorial modules. For cluster $k$, we first perform pairwise assessments of TF combinatorial effects. In the definition of the combinatorial effects for a pair of TFs in a cell cluster, we have two considerations: (1) TFs should have high ability to co-regulate other TGs in this cluster, i.e., they should have high connection specificity index (CSI) [84]; (2) TFs should have specific activity in this cluster relative to other clusters, i.e., they should have high expression specificity index (ESI). Then, the combinatorial effect of a pair of TFs is the product of the CSI score and the ESI scores of these two TFs:

1 $$\begin{array}{c}C_{ij}^k={CSI}_{ij}^k\cdot{ESI}_i^k\cdot{ESI}_j^k\end{array}$$

Here ${CSI}_{ij}^{k}$ is connection specificity index of $i$ th TF and $j$ th TF in cluster $k$, ${ESI}_{i}^{k}$ is expression specificity index of $i$ th TF in cluter $k$. High combinatorial effect is an indication of strong co-regulation by two TFs with specific activities in the cell cluster. This procedure generates $K$ combinatorial effect matrices ${C}^{1}$,${C}^{2}$,$\cdots$, ${C}^{K}$ from $K$ TF-TG regulatory strength matrices ${R}^{1}$,${R}^{2}$,$\cdots$, ${R}^{K}$, where ${C}^{k}$ contains the pairwise combinatorial effects (among the TFs) in cluster $k$. See “Methods” for details of the computation of the $C$ matrices from the $R$ matrices.

Then, an optimization model is developed to identify $L$ TF combinatorial modules underpinning *K* combinatorial effect matrices ${C}^{1}$,${C}^{2}$,$\cdots$, ${C}^{K}$. Formally, our optimization model is formulated as follows:

2 $$\begin{array}{c}\underset{X,A}{\text{min}}\sum\nolimits_k\left[{\Arrowvert C^k-{XA_kX}^T\Arrowvert}_F^2-\mu{\Arrowvert C^k\circ\left({XA_kX}^T\right)\Arrowvert}_1\right]\end{array}$$

$$\begin{array}{c}s.t.X\ge 0;{\sum }_{i}{x}_{il}^{2}\le 1;{\sum }_{l}{x}_{il}\le 1;l=\text{1,2},\cdots L,i=\text{1,2},\cdots M\\ {A}_{k}=diag\left({A}_{k1},{A}_{k2},\cdots ,{A}_{kL}\right);{A}_{k}\ge 0;{\sum }_{l}{A}_{kl}=1;k=\text{1,2},\cdots K\end{array}$$

The first term decomposes the TF combinatorial effect matrices in terms of TF modules. The ${j}$ th column of $X$ corresponds to the ${j}$ th TF-module in the sense that its large non-zero components indicate the key TFs in the module. The diagonal elements of the association matrix ${A}_{k}$ indicate the relative importances of the different TF modules in the combinatorial effect matrix for cell cluster $k$. The second term is a regularization term to encourage extracting TF modules with large combinatorial effects. Solving the optimization problem yields a solution represented by two variables $X$ and $A$. The TF modules can be obtained by thresholding the columns of $X$. Our method further extracts co-regulated REs and TGs for each TF module, culminating in the formation of $L$ different cRegulons. Furthermore, the association scores in the diagonal elements of ${A}_{k},$ which represent the strengths of associations between cRegulons and cell clusters, can serve as valuable tools for annotating cell types, individual cells, or defined cellular groupings. In essence, the collective representation of cRegulons, coupled with the association matrix, provides a useful picture of the cell type landscape (Fig. 1d).

Further details of data preprocessing, combinatorial effect definition, optimization model as well as algorithm, construction of TF modules and cRegulons, and the cell type annotation through cRegulon combinations can be found in the “Methods” section.

### Benchmarking cRegulon’s performance using synthetic regulatory modules and cell types

**Fig. 2:**
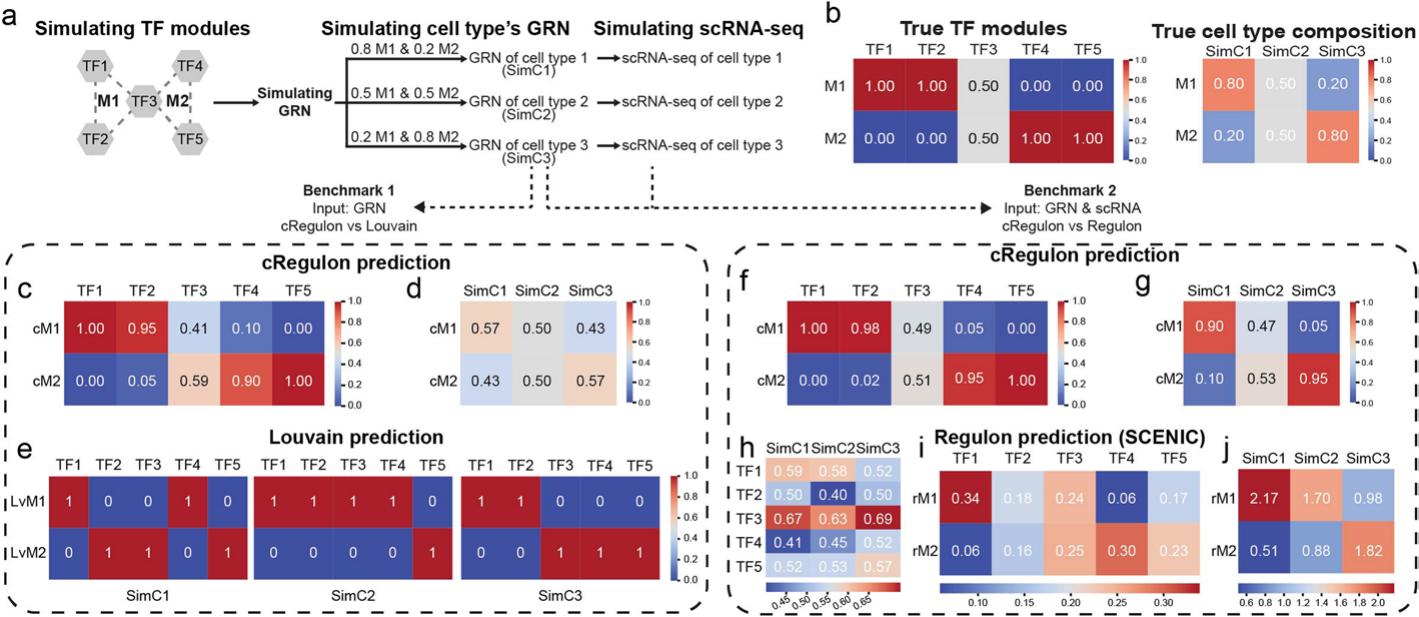
Benchmark with synthesized dataset. **a** The process of simulating cell type specific GRN and scRNA-seq data with different compositions of TF modules. **b** The true TF modules and cell type-TF module associations. With cell type-specific GRN as input, **c** the cRegulon predicted TF modules and **d** predicted cell type-TF module associations; **e** the Louvain method predicted TF modules for each cell type. With the input of simulated GRNs and scRNA-seq data, **f** the cRegulon predicted TF modules and **g** predicted cell type-TF module associations; **h** Averaged SCENIC TF activity across three cell types; **i** the SCENIC TF activity-based prediction of TF modules and **j** cell type-TF module associations

We first used an in-silico simulation as proof of concept to show cRegulon can identify modular regulatory units and their associations with preset cell types (Fig. 2a). Briefly, we manually created two TF combinatorial modules (M1: TF1/TF2/TF3, M2: TF3/TF4/TF5) with one overlapped TF (Additional file 1: Fig. S2a, b) and simulated three cell types’ GRNs (TF-TG regulatory strength matrix) with different weights of two TF modules (SimC1: 0.8 M1 & 0.2 M2; SimC2: 0.5 M1 & 0.5 M2; SimC3: 0.2 M1 & 0.8 M2, Additional file 1: Fig. S2c-g). Then we use SERGIO [85] to simulate scRNA-seq data for each cell type from their GRN (Additional file 1: Fig. S2h). Details of simulation can be found in “Methods.” We used the simulated dataset and its true TF modules and true cell type composition (Fig. 2b) to validate cRegulon alongside other methods, focusing on two key aspects: (1) the ability to accurately reconstruct TF modules and (2) the capability to reveal the module composition within each cell type.

Since TF modules detection in GRN can be seen as a traditional community searching problem in graph, we first compared cRegulon with Louvain method, which is a commonly used algorithm to detect modules. We applied cRegulon directly to the simulated TF-TG regulatory strength matrices (Additional file 1: Fig. S2e-g) of the three cell types to compute the CSI matrices for each cell type. Then we used cRegulon to identify two TF modules and their associations with the three cell types. It was important to note that this was not the formal version of cRegulon, as the formal approach combined CSI and TF activity to define the $C$ matrix. Figure 2c displays the $X$ matrix output by cRegulon, illustrating the weights of TFs in the two TF modules. We observed that cRegulon-identified TF module 1 (cM1) consisted of TF1, TF2, and TF3, while cM2 comprised TF3, TF4, and TF5. This perfectly reconstructed the TF modules as simulated. Figure 2d shows the cRegulon-predicted association between TF modules and cell types. We found that SimC1 was dominated by cM1, SimC3 was dominated by cM2, and SimC2 had relatively balanced weights of cM1 and cM2, which aligns with the truth (Fig. 2b). These findings demonstrated that cRegulon can accurately identify TF modules and their relationships with cell types. We compared cRegulon’s performance with community detection algorithms by applying the Louvain algorithm [86] from the “networkx” package to the simulated R matrices. To obtain two TF modules, we increased the resolution parameter of Louvain algorithm from 0.05 with a step of 0.05 and stopped when two modules were identified (Fig. 2e). For SimC1, the Louvain method incorrectly identified TF1 and TF4 as a module and TF2/TF3/TF5 as a module, failing to capture the fact that TF1/TF2/TF3 function as a cohesive module dominating SimC1. In the case of SimC2, the Louvain method incorrectly put TF4 into the same module of TF1-TF3. Only the TF modules of SimC3 seemed reasonable, where TF1 and TF2 were in the same module and TF3, TF4, and TF5 were in the same module. These results indicated that accurate inference of TF modules cannot be achieved by directly applying existing module detection methods to the cell type-specific GRNs.

Another widely used method for scRNA-seq data analysis is SCENIC [44], which constructs regulons for TFs and employs AUC scores to assess TFs’ activity in individual cells. Next, we compared cRegulon to SCENIC with additional expression data as input. In our formal model of cRegulon, the TF activity specificity is combined with the CSI matrix to define the final $C$ matrix. Using the simulated scRNA-seq data, we calculated TF specificity across different cell types and input this data into cRegulon. Incorporating TF specificity allowed cRegulon to produce results that were more closely aligned with the truth. For example, cRegulon still identified that cM1 consisted of TF1, TF2, and TF3, while cM2 comprised TF3, TF4, and TF5. Moreover, the scores of TF2 and TF4 were closer to 1.0. And the scores of TF3 were more balanced between the two modules (closer to 0.5) (Fig. 2f). Additionally, with TF specificity factored in, annotation to SimC1 (0.9 cM1 and 0.1 cM2) was more consistent with the fact that it had a large strength of M1 (0.8) and small strength of M2 (0.2). This improvement was also observed from SimC3’s annotation (0.95 cM2 and 0.05 cM1), which was more consistent with a large strength of M2 (0.8) and small strength of M1 (0.2) in our simulation (Fig. 2g). Thus, incorporating TF’s specificity into cRegulon clarified TF modules and their associations with cell types. For a fair comparison, we modified the SCENIC workflow by replacing the GRN and regulon construction steps with pre-defined regulons from simulated GRNs. We then used the “AUCell” function to calculate the activity scores of these regulons in each cell, averaging the results across cell types (Fig. 2h). The resulting TF regulon activity matrix was decomposed using non-negative matrix factorization (NMF) to identify TF modules and cell types’ TF module compositions. However, the regulon-based approach did not provide as precise TF modules as cRegulon. For example, in Fig. 2i, TF2 exhibited similar scores across both rM1 and rM2, making it difficult to determine which module it belonged to. Furthermore, TF5, which should not be included in rM1, had a high score in rM1. In addition, the regulon-based method failed to accurately capture the association between cell types and TF modules, particularly in cases of cell type with mixed TF modules. As shown in Fig. 2j, the regulon-based approach incorrectly predicted that SimC2 had a two-fold higher score for rM1 compared to rM2, whereas SimC2 should have balanced contributions from both modules.

In summary, our in-silico simulation and benchmark show cRegulon’s unique advantages over existing methods in two aspects: (1) cRegulon identifies more accurate TF modules and (2) cRegulon accurately reveals the composition of TF modules in each cell type.

### Benchmarking with real single cell data

To show cRegulon can reveal better biologically meaningful regulatory units, we simulated another dataset by mixing single-cell data from four distinct cell lines—BJ (fibroblasts), H1-ESC (embryonic stem cells), K562 (erythroleukemia), and GM12878 (lymphoblastoid)—to simulate data including both similar cellular contexts (K562 and GM12878) and dissimilar contexts (BJ and H1-ESC). We intended to evaluate the ability of cRegulon to reveal biological characteristics of regulatory units and to compare its performance against alternative methods.

**Fig. 3:**
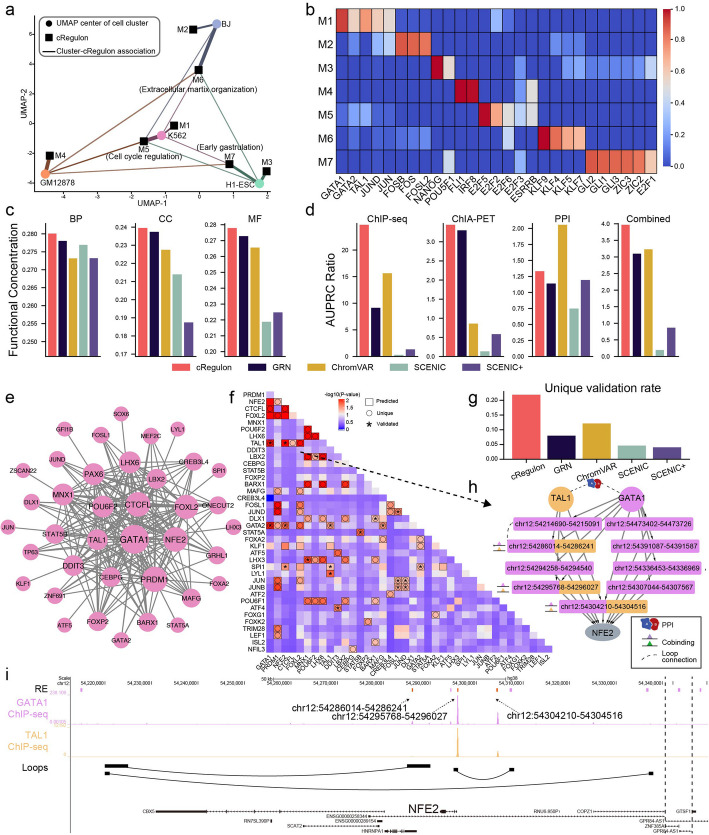
Benchmark with real dataset by mixing four cell lines. **a** The association plot between 4 cell lines and 7 inferred cRegulons. The thickness of lines indicates the association score. Only association scores larger than 0.02 are plotted. **b** Heatmap of marker TFs’ combinatorial effect shows cRegulon can precisely capture the landmark TFs of cell types. **c** Comparison of averaged functional concentration for BP, CC, and MF. **d** Validation of TF combinations with ChIP-seq, ChIA-PET, PPI, and combined dataset. **e** TF module of M1, specific to K562. **f** The unique and validated TF combinatorial regulation in M1. The squared frame indicates predicted TF combination. Circle label indicates this TF combination can only be predicted by cRegulon, without presence in other methods’ predictions. Star indicates this TF combination is validated by TF ChIP-seq data, ChIA-PET loops, or PPI dataset of K562. **g** Comparison of unique-validation rate, the proportion of validated TF pairs in uniquely identified TF pairs. **h** Combinatorial regulatory network of GATA1 and TAL1 on NFE2. **i** Genomic tracks show the REs, GATA1 ChIP-seq, TAL1 ChIP-seq, and Hi-C loops that support the coregulation of TAL1 and GATA1

The application of cRegulon to this simulated dataset derived 7 cRegulons associated with 4 cell lines. Scrutinizing these 7 cRegulons (termed as M1-M7) and their association plot (Fig. 3a) with 4 cell types (Methods), we found cRegulon prioritized pivotal TFs within their respective TF modules (Fig. 3b), which had consistent functions with their associated cell types. Some cRegulons displayed specificity toward cell types. For instance, M1 was exclusively associated with K562. It featured GATA1/2 and JUN/D/B, which are well-known regulators for K562. M2 demonstrated specificity toward BJ and was marked by FOS TF family (FOSB, FOS, FOSL2). The TGs of M2 were enriched in “collagen fibril organization,” “supramolecular fiber organization,” and “epithelial to mesenchymal transition” (Additional file 2: Table S1), which were consistent with functions of BJ. M3 only exhibited association with H1-ESC, featuring NANOG and POU5F1 within its TF module [87]. M4 was specific to GM12878 and prominently showcased IRF8 and FLI1, which are important lymphocyte regulators. Conversely, some cRegulons showed shared associations across different cell types. M5 showed a marked presence of the E2F TF family—E2F5/2/6/3—known for its role in regulating the cell cycle [88]. Additionally, ESRRB, which is associated with cell cycle processes [89], also appeared as one of the top TFs within M5. M5 exhibited associations across all four cell lines, a plausible observation given the necessity of cell cycle regulation in maintaining cell lines. Similarly, M6 displayed associations with all four cell lines and a closer affinity to BJ. M6 had KLF family (KLF9/4/5/7), and its TGs also enriched in fibroblast functions and extracellular matrix organization, such as “collagen fibril organization,” “extracellular matrix organization,” and “elastic fiber assembly” (Additional file 2: Table S1). M7 encompassed the E2F1, GLI family (GLI1/2/3), and ZIC family (ZIC2/3), suggesting that M7 be involved in cell lineages in the early gastrulation [90–92]. M7 was also linked to cell cycle and cell division control [93–96], which make M7 to be associated with most cell lines. Overall, the functions of the modules are well aligned with those of the cell types to which they are associated with.

We next evaluated if the identified cRegulons revealed characteristics of regulatory units and made comparison with other methods. On the one hand, regulatory unit is expected to have specific functions or biological processes in the cell, and its associated TFs should show concentrated enrichment in GO terms related to those functions and processes. We used the GOSemSim package to compute the similarity of enriched GO terms as an approximation for functional concentration. From the association plot (Fig. 3a), we found M1 was specific to K562, M2 specific to BJ, M3 specific to H1-ESC, and M4 specific to GM12878. We compared GO term similarities inferred from cRegulon with cell type-specific TFs from alternative methods, including GRN, ChromVAR, SCENIC, and SCENIC + (Methods). From Fig. 3c, we observed that, on average of four cell lines, cRegulons had higher functional similarity than other methods, showing a better functional concentration characteristic of regulatory units. On the other hand, to achieve combinatorial regulation, the TFs in a regulatory module are expected to interact with each other in various ways. We used several independent datasets to validate putative interactions between pairs of TFs within a TF module. In the physical realm, TFs may cooperate or interact via co-binding at REs, chromatin loop linkages, and protein interactions. Therefore, we utilized K562 as an example and employed ChIP-seq, ChIA-PET, and PPI data to establish ground truths for co-binding, loop-connected, and protein interaction TF pairs. We also constructed a combined ground truth by integrating evidence from three resources (Methods). We compared cRegulon with 4 baseline methods (abbreviated as ChromVAR, SCENIC, SCENIC + and GRN, details in Methods). Briefly, the first three methods came from the TF module identification pipeline described in Suo et al. [80] and are based on TF activity scores computed by ChromVAR, SCENIC, and SCENIC + respectively; the fourth method directly utilized GRN TF mutual regulatory strength to identify TF modules [56, 97]. We obtained the K562’s TF combinatorial effect and calculated AUPR ratio (Methods) as metric to evaluate the accuracy of TF combinations from different methods. From Fig. 3d and Additional file 2: Table S2, only ChromVAR performs better than cRegulon in the situation of PPI, and for the other ground truth, including the combined ground truth, cRegulon performs best among the five methods. We can use Gamma distribution assumption on TF combinatorial effect and *P*-value 0.05 as threshold to obtain a set of TF pairs of different methods (Methods). cRegulon predicted 199 TF pairs in M1, which was specific to K562. GRN, ChromVAR, SCENIC, and SCENIC + predicted 76, 211, 138, and 234 TF pairs for K562, respectively. From Additional file 1: Fig. S3, only on for ChIA-PET, SCENIC + performs best for recall and F1 score, and for all other comparison scenarios, cRegulon is the best for precision, recall, and F1 score. TF combinations could also be inferred by combining TFs’ motif similarity with identified TFs, which can derive four additional methods: GRN + Motif, ChromVAR + Motif, SCENIC + Motif, and SCENIC + + Motif (Methods, Additional file 1: Fig. S4a). We compared cRegulon with them on K562 and found cRegulon still had the best performance (Additional file 1: Fig. S4b). These validations and comparison unequivocally established the efficacy of cRegulon model in recovering two important aspects of regulatory units: functional specificity and combinatorial regulation via TF interactions.

Many of the TF combinations detected by cRegulon are unique and not discovered by any other methods. For example, there are 78 TF combinations in the K562-specific module M1 (Fig. 3e), and 64 of them were only identified by cRegulon. And among these uniquely identified TF combinations, 14 TF pairs were validated by ChIP-seq data, ChIA-PET loop, or PPI dataset (Fig. 3f). To evaluate the ability of a method for identifying novel TF combinations, we defined its unique-validation rate as the validation rate of the set of TF pairs uniquely identified by this method (i.e., not identified by any of the other competing methods). cRegulon gave a much higher unique-validation rate (0.22) than other methods (Fig. 3g), indicating superiority in finding novel TF combinatorial regulations. We also noticed that some TF pairs were not validated because we do not have their ChIP-seq data in K562. For example, no ChIP-seq data are available for JUN and FOXL2, one un-validated but unique TF pairs of cRegulon. Additional ChIP-seq or other types of data could potentially validate these predicted TF combinations. For instance, Thomas M. Norman et al. constructed combinatorial overexpression libraries involving 112 genes and calculated a genetic interaction (GI) score to assess the epistatic effects of gene pairs [98]. Their findings revealed that the JUN-FOSL2 had a significant epistatic effect, with an absolute *z*-score of 1.8 for the GI score. We also observed that the TF combinations uniquely identified by cRegulon are relevant to the cancer state and erythroid state of K562. For example, the STAT5 family members (STAT5A and STAT5B) were included in M1, which are known for their roles in anti-apoptosis and tumorigenesis [99]. On the other hand, GATA1 and TAL1 are key TFs defining the erythroid identity: GATA1 is a marker of K562 [100, 101]; TAL1 is involved in myeloid cell differentiation and is a positive regulator of erythrocyte differentiation [102]. Previous studies have demonstrated a precisely organized complex formed by TAL1 and GATA1 [103] and our finding recapitulated this. In the regulatory network of K562, the regulations of TAL1 and GATA1 were largely shared (Additional file 1: Fig. S5a). TAL1 and GATA1 bound to 6720 and 15,553 REs, respectively, with 6528 REs being shared between the two TFs, yielding a Jaccard similarity of 0.41 and a significant *p*-value of 0.029 by permutation test. Moreover, TAL1 regulated 3018 TGs, and GATA1 regulated 6227 TGs, with a Jaccard similarity of 0.48 and a significant permutation test *p*-value of 0.030 among their common TGs. Among their 2991 common TGs, NFE2 emerged as one of the top co-regulated genes, and the cRegulon model provided intricate insights into the combinatorial regulatory network orchestrating the co-regulation on NFE2 by TAL1 and GATA1 (Fig. 3h). TAL1 bound to 3 REs to regulate NFE2 and GATA1 had 9 REs for NFE2, where 3 REs were shared. This combinatorial regulatory network was supported by substantial independent evidence. For example, TAL1 and GATA1 have strong protein interaction potential to form complex [104, 105]. The 3 shared REs are supported by common ChIP-seq peaks of TAL1 and GATA1 (Fig. 3i), suggesting their capability to bind to the same genomic regions for co-regulation. Additionally, for the non-shared RE, GATA1 at chr12:54,214,690–54215091 was observed to form chromatin loops with TAL1 at chr12:54,286,014–54,286,241; and chr12:54,307,044–54,307,567 of GATA1 had a chromatin loop with chr12:54,295,768–54,296,027 of TAL1 (Fig. 3i), implying that while TAL1 and GATA1 might bind to different REs connected by long-range chromatin loops. These examples illustrate the potential of detecting biologically relevant TF combinations by cRegulon.

Finally, in the Additional file 1:Supplementary Texts, we analyzed the impact of incorporating more cellular contexts into cRegulon analysis (Additional file 1: Fig. S5b-h), conducted ablation studies to show the importance of cRegulon’s components (Additional file 1: Fig. S6), designed experiments to show the robustness of cRegulon to cell clustering (Additional file 1: Fig. S7) and single-cell data that are not paired for same cell (Additional file 1: Fig. S8) and showed cRegulon’s extensiveness to study RE combinations (Additional file 1: Fig. S9).

### Application to human fetal atlas reveals regulatory units decoding both spatial and temporal states of early human development

**Fig. 4:**
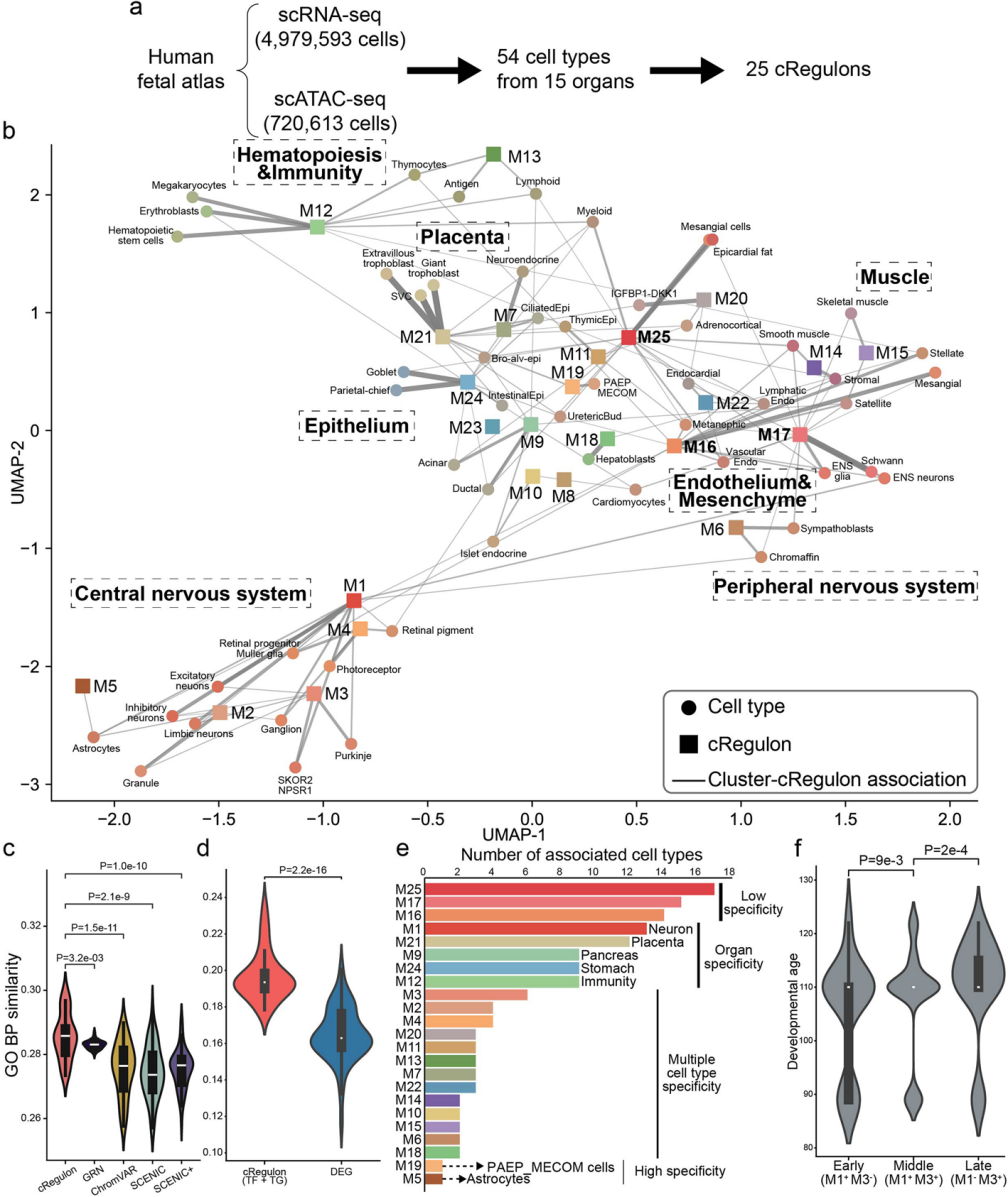
cRegulon reveals the landscape of early human development. **a **Application to human fetal atlas gives 25 cRegulons underpinning 54 cell types of 15 organs. **b** Association plot shows the relationship between 25 cRegulons and 54 cell types. The thickness of lines indicates the association score. Only cell type-cRegulon association scores that are no less than 0.1 are displayed. **c** The GO BP similarity scores of TFs from cRegulon, GRN, ChromVAR, SCENIC, and SCENIC +. **d** The GO BP similarity score of cRegulon genes (TFs + TGs) and DEGs. **e** cRegulons’ spatial influence shows both specificity and commonality. The *y*-axis is the number of associated cell types. **f** cRegulons’ temporal influence predicts intermediate states of excitatory neurons (*x*-axis, Additional file 1: Fig. S11g), which show significant and consistent differences in embryo ages (*y*-axis).

To assess the utility of our method in large-scale single-cell data analysis, we applied our model to the human fetal atlas with 4,979,593 scRNA-seq cells and 720,613 scATAC-seq cells. We balanced the cell numbers across different cell types using a sampling strategy [10] and obtained a dataset comprising 329,464 scRNA-seq cells and 68,944 scATAC-seq cells covering 54 distinct and well annotated cell types derived from 15 organs. From this data, our method inferred 25 cRegulons that can be used as regulatory units to elucidate the landscape of early human developmental processes (Fig. 4a).

These 25 cRegulons, denoted as M1-M25, exhibited diverse functionalities, which might serve as modular regulatory units that could be used in different ways in different cellular contexts (Table 1). To understand the roles and functions of a cRegulon, we examined the functions of the top TFs in the module and performed functional enrichment analyses of both TFs and TGs within the cRegulon (Additional file 2: Table S3). Using this approach, M1 to M8 were seen to be predominantly associated with neural states: M1 portrayed the neural progenitor state; M2 delineated postmitotic early neurons; and M3 depicted postmitotic late neurons. Furthermore, M4 was characterized by the VAX, RAX, and VSX families, known markers for ocular and retinal development [106–108]; M5 was responsible for the neuroglia; M6 was related to sympathetic system; M7 and M8 were also marked by neural TFs, such as NEUROD1 and NEUROG1, and demonstrated associations with neural functions in non-CNS organs, such as the neuroendocrine and neuromuscular systems. M9 and M10 showed enrichments in pancreatic exocrine and exocrine functions, respectively. M11 (immune cells), M12 (hemopoiesis), and M13 (cytokine signals) manifested functionalities associated with hematopoietic and immune roles. Additionally, M14 (muscle organ development) and M15 (muscle structure) were distinctive to muscular states. The spectrum of functions encompassed by M16-M22 spanned mesodermal and endodermal organs: M16 and M17 included a cluster of HOX genes linked to anterior–posterior patterning of internal organs and skeletal muscles, respectively; M18 was associated with liver functions, including lipid and glucose homeostasis as well as transport activity; M19 with kidney development; M20 with adrenocortical functions; M21 with placental functions and M22 with angiogenesis in the cardiac system. Finally, three cRegulons were identified to be associated with epi-endothelial functions, namely M23 and M24 responsible for epithelial transport and structure, and M25 enriched in endothelial tissues.

**Table 1:** Twenty-five cRegulons for human fetal atlas, as well as their top TFs and enriched biological functions

	cRegulon	Top TFs	Enriched functions
Neural state	M1	FOXG1, RFX4, NEUROD2, NEUROG2, ASCL1, ESRRB	Neural progenitor state
	M2	PAX3, RFX4, POU3F2/F3, OLIG1, NEUROD2, PAX6	Postmitotic early neuron
	M3	TFAP2A, LHX-, POU3F2, OLIG, EN-	Postmitotic late neuron
	M4	VAX-, RAX-, VSX-, NEUROD/G-, PAX6	Ocular development
	M5	OLIG1/2/3, PAX6, NR2E1, VAX1, SOX8, SOX9, GLI3	Neuroglia
	M6	TFAP2B, SOX11, ASCL1, PHOX2B, PHOX2A	Sympathetic system
	M7	NEUROD1, HNF1A, FOXA2	Neuroendocrine system
	M8	HMX3, NEUROG1, POU4F2, DBX2	Neuromuscular system
Pancreatic state	M9	HNF4A, HES1, PROX1, HNF1A, ONECUT1, GATA6, PDX1	Pancreas exocrine
	M10	GATA6, INSM1	Pancreas endocrine
Hemopoiesis and immunity	M11	MAFB, SIX4, SIX1, TCF7, LEF1, STAT4	Immune cells
	M12	STAT5B, TAL1, GATA2, GATA1	Hemopoiesis
	M13	IRF4, IRF1, IRF7, IRF8, IRF5, IRF9	Cytokine signals
Muscular state	M14	FOXF1, TBX18, NFATC4, TBX2, HLX, NR2F2, MSC, MEOX2	Muscle organ development
	M15	MYOG, TBX1, MYOD1, MYF6, MYF5	Muscle structure
Mes-endoderm state	M16	HOX-, WT1, FOXC2, GLI3, ARID5B, TCF21, GLI2	AP pattern of internal organs
	M17	HOX-, HEY2, TBX3, NFATC4, TBX2	AP pattern of skeleton and muscle
	M18	CEBPA, XBP1, ONECUT2, HNF4A, ONECUT1, HNF1A, NR1H3, PPARG	Lipid-glucose homeostasis and transport activity
	M19	FOXB1, PAX8, PAX2	Kidney development
	M20	OSR2, HLX, MYC, TWIST1, TWIST2, MSX1, FOXO4	Adrenal gland
	M21	CEBPA, CEBPB, EPAS1, GATA2, DLX-	Placental development
	M22	FOXC1, EPAS1, TAL1, ID1, MEOX2, ELK3	Angiogenesis
Epi-endothelial state	M23	AIRE, FOXN1, PRDM1, SPDEF	Epithelium transport
	M24	JUN-, FOS-, FOXA1, SOX9, GRHL2, RREB1	Epithelium structure
	M25	JUN-, FOS-, FOXC1, KLF5, ID1, HOXB3, MEOX2	Endothelium

The association analysis between 25 cRegulons and 54 cell types portrayed a comprehensive landscape of early human development. UMAP visualization based on the cRegulon annotation vectors of the cell types revealed that cRegulons effectively recapitulated both anatomical and functional classifications (Fig. 4b, Additional file 1: Fig. S10a). For instance, neural, retinal, and glial cell types, constituting the central nervous system, were governed by M1-M5, aligning well with their neural functionalities. The peripheral nervous system, encompassing ENS neurons, ENS glia, Schwann cells, sympathoblasts, and chromaffin cells, was clustered together. M6, which was a cRegulon specific to the sympathetic system, controlled this system: M6 had strong association scores (> 0.3) with sympathoblasts and chromaffin cells (Fig. 4b); and it also had associations (> 0.01) with ENS neurons, ENS glia, and Schwann cells (Additional file 2: Table S3). Additionally, hematopoietic cell types and immune cell types clustered closely due to their association with M12 and M13, indicative of hematopoiesis and immune functions. Muscular cell types showed close associations owing to the annotation of M14 and M15, dedicated to muscular states. Similarly, the association of cell types with meso-endoderm cRegulons (M9/10/18/19) demonstrated the diverse functions observed in pancreas, liver, stomach, and intestine cell types. Epithelial cell types, such as intestinal epithelial cells, bronchiolar-and-alveolar epithelial cells, and ciliated epithelial cells, were together and governed by M23 and M24. Notably, endothelial cell types and mesenchymal cell types shared closer relationships mainly governed by M25, M22, M16, and M17. M25 and M22 tended to be responsible for endothelial functions, while M16 and M17 were more related to anterior–posterior pattern. This distribution of cell types was consistent with the previously published human fetal atlas [10], wherein our model further revealed the cRegulons as the underlying functional units contributing to the early human developmental landscape.

The annotation by cRegulon can capture specific characteristics of cell types. We hypothesized that the genes in a good functional annotation should have strong concentration in functional enrichment, which means genes are enriched in GO terms that are closely placed in the GO hierarchy. GOSemSim [109] can evaluate the similarities of associated GO terms for genes to access their concentration in functional enrichment. Using GOSemSim (Methods), we first compared the functional enrichment concentration of TFs in cRegulon with TFs inferred by GRN, ChromVAR, SCENIC, and SCENIC +. We found that cRegulon TFs exhibited a higher concentration of functional enrichment than TFs from other methods across biological pathways (BP, Fig. 4c), cellular components (CC, Additional file 1: Fig. S11a), and molecular functions (MF, Additional file 1: Fig. S11b). Next, we compared the entire gene sets of cRegulon (TFs and TGs) with gene sets inferred by differential expression (DEG genes). In the biological process hierarchy of GO, cRegulon genes showed significantly higher concentration than DEGs across every cell type (Fig. 4d, Additional file 1: Fig. S11c). Similarly, for CC and MF, cRegulon also exhibited higher functional similarity scores compared to DEGs (Additional file 1: Fig. S11d, e). This observation underscores cRegulon’s superior concentration of functional enrichment compared to conventional methods. The enhanced functional enrichment annotation of cRegulon can be further demonstrated in detail in the following two examples. In the first example, we examined the association score of hematopoietic stem cells (HSCs) and found that it is strongly annotated with M12. We checked the functional enrichment of M12’s top 20 TFs in its TF module and top 200 TGs. M12 was enriched in many immune and hematopoietic pathways, such as “erythrocyte differentiation,” “eosinophil differentiation,” “myeloid cell apoptotic process,” and “negative regulation of myeloid cell apoptotic process” (Additional file 2: Table S4). However, in the top enriched pathways of HSCs’ top 200 DEGs, no immune terms were found. For top 20 TFs identified by ChromVAR, we observed one immune cell associated pathway: “negative regulation of erythrocyte differentiation”. For top 20 TFs identified by SCENIC, only one pathway (“definitive hemopoiesis”) was consistent with the cell type’s functions. For the top 20 TFs identified by SCENIC +, there are no enriched immune pathways. In the second example, we examined the annotation of Syncytiotrophoblasts-and-villous-cytotrophoblasts (SVCs) and found that this cell type was only associated with M21. M21’s Top 20 TFs and Top 200 TGs were enriched in “embryonic placenta development,” “positive regulation of fat cell differentiation,” and epithelial functions (“epithelial cell maturation” and “mammary gland epithelial cell differentiation”) (Additional file 2: Table S5), which were highly relevant functions of placenta cell type SVCs. In contrast, the other methods resulted in much less relevant functional enrichment: DEG and SCENIC analysis led to enrichment only in fatty acid associated functions; ChromVAR and SCENIC + failed to reveal any functions related to placental development. These comparisons demonstrate that cRegulon provides more specific functional annotations than alternative methods.

The 25 cRegulons exhibited different degrees of specificities in their association with cell types. Figure 4e provided a ranking of these cRegulons based on the number of their associated cell types showing their diverse ranges of impacts. Some cRegulons were specific to only one or two cell types, signifying their usage was highly cell-type-specific. For instance, M5, with neuroglial functions, exhibited specificity toward astrocytes; M19 was exclusively present in “PAEP MECOM positive cells,” thereby linking this cell cluster with nephritic functions. In contrast, some cRegulons were associated with multiple cell types, but were limited to certain organs or tissues. For instance, M1 was associated with 13 cell types, all exclusively neural; M21 was linked to 12 cell types, predominantly placental. Moreover, most enriched cell types for M9, M24, and M12 were from pancreas, stomach, and immunity, respectively. Finally, there were some cRegulons (such as M25, M16, and M17) that have very low degree of cell-type-specificity. They were associated with a minimum of 1/4 of the cell types, spanning many organs (9 organs for M17, and 4 for M16, Fig. 4b, e, Additional file 1: Fig. S10b). This indicated their spatially broader influence on human embryos compared to other cRegulons. Examination of their top 50 TFs revealed that all three cRegulons featured a set of HOX genes (Additional file 1: Fig. S11f), known for their multifaceted roles in early development [110], potentially explaining the wider cell type associations of M25, M16, and M17 (Fig. 4b).

Some of the cRegulons from the human fetal atlas are informative on developmental stages. We considered the cRegulon M1 as an example, which was broadly associated with all neuron cell types (Fig. 4b). Since M1 was enriched in progenitor functions, we attributed its broad associations to the presence of progenitors in the various neural cell types. To validate this hypothesis, we examined the excitatory neurons, which were strongly associated with M1 (Fig. 4b, Additional file 1: Fig. S10a). To reveal the early and intermediate role of M1, we categorized excitatory neurons into early, middle, and late groups based on their M1 and M3 association (Methods, Additional file 1: Fig. S11g). Subsequent observation revealed that the early group cells exhibited the smallest developmental age of the fetal samples, the middle group featured intermediate age, and the late group displayed the largest age, demonstrating significant differences among these groups by *t*-test analysis (Fig. 4f). These findings underscored the capacity of our model in identifying regulatory units relevant to developmental stages.

### cRegulons from human fetal atlas provide useful annotation for new cell subtypes

The panel of regulatory units from atlas-scale dataset can be used to facilitate precise annotations for cell types from new datasets. This annotation further allows us to compare the cell type/state by revealing differential TF modules and cRegulon compositions. Such analysis should be conducted in biologically (e.g., developmental divergence) and technical (e.g., dataset heterogeneity) relevant dataset. Given that human fetal and adult β cells exhibit high conservation of core transcriptional networks [111] and fetal and adult pancreatic endocrine cells demonstrate consistent expression patterns [112], we conducted an exploratory experiment to show the annotation and differential analysis ability of cRegulon on an external beta cell dataset.

**Fig. 5:**
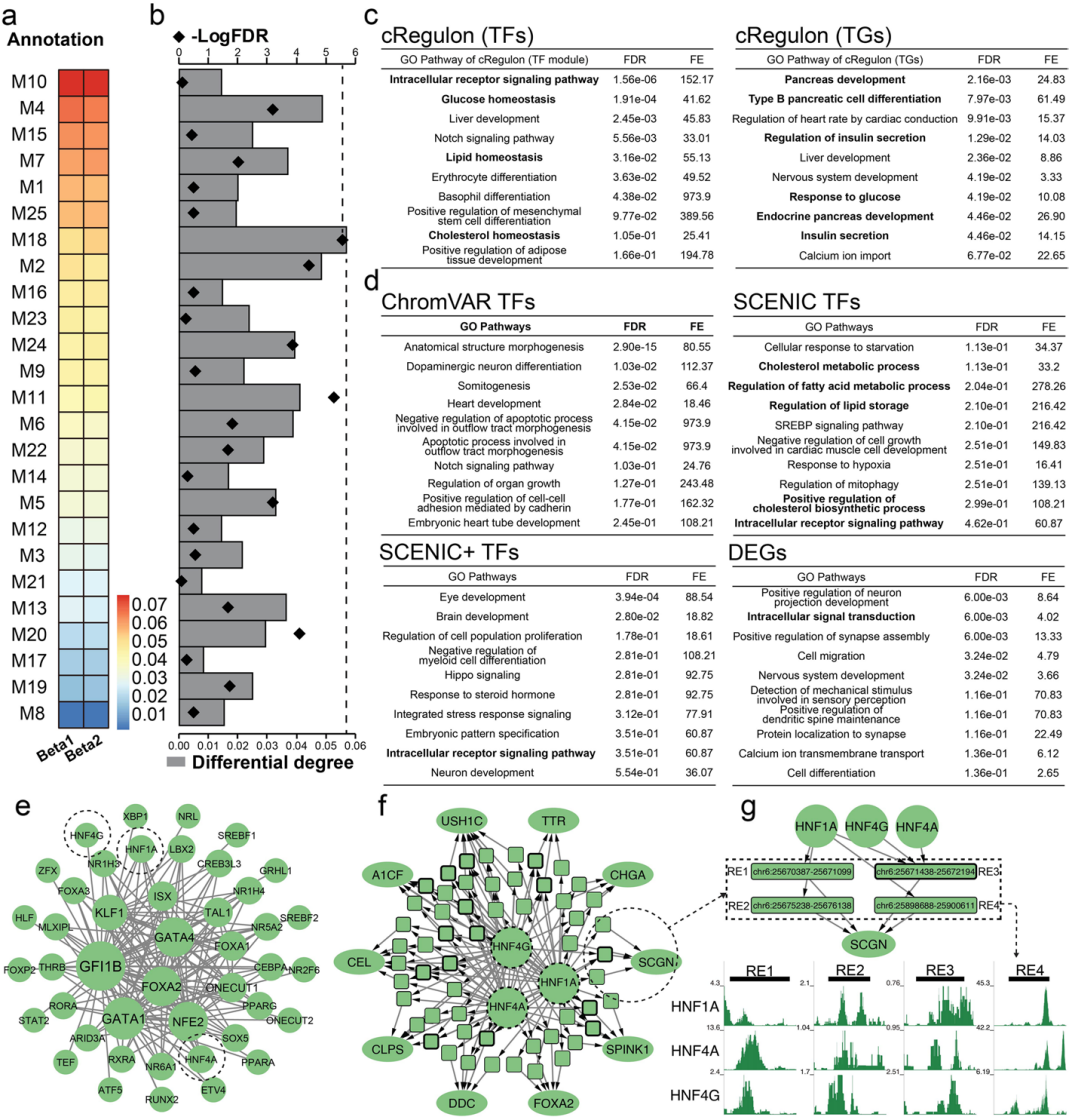
Twenty-five cRegulons from human fetal atlas can annotate external cell types and their difference. **a** The cRegulon annotation scores of two beta subtypes. **b** The differential degrees and their FDR adjusted *P*-value of cRegulon annotation scores to two beta cell subtypes. **c** Functional enrichment of top 20 TFs and top 200 TGs from M18. **d** Functional enrichment of differential ChromVAR TFs, differential SCENIC TFs, differential SCENIC+ TFs, and differentially expressed genes. **e** TF module of M18. **f** Combinatorial regulatory network of HNF1A, HNF4A, and HNF4G on their top 10 TGs. **g** The local regulation of HNF1A, HNF4A, and HNF4G on SCGN. The predicted co-binding REs are validated by ChIP-seq data

We acquired single-cell multi-omics data for two β subtypes [113], beta1 and beta2, distinguished by their associations with “non-diabetic” and “diabetic” states, respectively. Annotating these beta subtypes with our 25 cRegulons revealed highly similar annotation patterns (Fig. 5a). Notably, both subtypes exhibited strong associations with M10, responsible for pancreas endocrine functions, and M7 for neuroendocrine (Table 1). Additionally, the common association between M4 (Ocular development) and both subtypes was caused by the top TFs’ activities in pancreatic cells, such as NFE2L3 [114], PAX6 [115], and NEUROD1 [116, 117]. Furthermore, both subtypes displayed strong associations with M15 (Muscle structure) because of its top TFs’ multiple roles in muscular and pancreatic functions, such as MAFA [118, 119] and RXRG [120, 121].

We then performed the differential analysis of each cRegulon by integrating absolute and relative fold differences between the two β subtypes (Methods). Notably, the differential degrees between two β subtypes were small, indicating two beta subtypes were similar. M18 exhibited the highest differential degree, indicative of the most likely functional disparities between the subtypes (Fig. 5b). We used permutations on beta cells to conduct a hypothesis test to see if these differential degrees of cRegulon are significantly different from zero (Methods), and we found M18 also gave the most significant *P*-value (Fig. 5b). Functional enrichment analysis of M18 showed its top 20 TFs were enriched in cholesterol-glucose homeostasis, and its top 200 TGs were associated with pancreatic cells and insulin secretion, such as “pancreas development,” “type B pancreatic cell differentiation,” “regulation of insulin secretion,” “response to glucose,” “endocrine pancreas development,” and “insulin secretion” (Fig. 5c). This is consistent with the previous findings that these two beta subtypes are different in the ability of exocytosis [113]. This ability to provide fine-grained annotation on new cell types seems to be unique to our method: ChromVAR TFs did not show enrichment in functions associated with lipid or glucose. SCENIC TFs only showed enrichment in cholesterol associated terms, such as “cholesterol metabolic process,” “regulation of fatty acid metabolic process,” and “regulation of lipid storage,” without enrichment in glucose or pancreatic functions. SCENIC + TFs only showed enrichment in “Intracellular receptor signaling pathway.” We also compared with the top 200 DEGs. However, DEGs only gave one associated pathway, “intracellular signal transduction,” without enrichment in cholesterol-glucose homeostasis or pancreatic functions (Fig. 5d).

In addition to its ability to identify functional differences of two β subtypes, cRegulon offered insights into the intricate combinatorial regulation of the differential cRegulon M18. We used combinatorial regulation of three important TFs (HNF1A, HNF4A, and HNF4G) to demonstrate M18’s revealed functional difference between β subtypes. Because the differential role of HNF TFs has been reported by previous work [113] but their regulation remained unknown. cRegulon facilitated the delineation of the combinatorial regulatory network of HNF TFs, revealing their common TGs tightly associated with diabetes and insulin transport (Fig. 5e). For example, SCGN is a β-cell enriched, secretory/cytosolic Ca2 + -binding protein [122]. FOXA2 regulates multiple pathways of insulin secretion [123]. CEL is associated with Maturity onset diabetes of the young, type 8 (MODY8) [124]. Ablation of CHGA affects the islet volume, the composition, distribution, and nuclear size of islet cell types and plasma insulin concentration [125]. We can also use cRegulon to study the local co-regulation of HNF TFs on TGs, such as SCGN (Fig. 5f). We found four REs were used by HNF TFs to regulate SCGN, where RE3 was bound by all three TFs and the other three REs were bound only by HNF1A (Fig. 5g). These co-binding REs are supported by ChIP-seq of HepG2, which also has strong molecule transport functions [126]. Interestingly, this combination was not identified by other methods: SCENIC failed to recognize HNF4A as a regulon for either β subtypes; ChromVAR and SCENIC + were unable to prioritize either of these TFs. Motif-based methods were ineffective due to the different motifs of HNF1A and HNF4A. Specifically, we measured their motif similarity using PWM matrices, resulting in a similarity score of 0.29 with a *z*-score of 0.2 (the average motif similarity between TFs is 0.27). This indicates that these two TFs could only be identified by cRegulon. Furthermore, in vivo knock-out experiments in islets have been conducted to reveal epistatic effects between HNF1A and HNF4A [127], which provided a strong validation for this unique finding from cRegulon. Notable, in M18, there were TFs with higher combinatorial effects that were also tightly associated with pancreatic insulin’s functions (Fig. 5e), such as FOXA2 [123] and ISX [128], which were more likely to be associated with beta cell difference. cRegulon showed their combinatorial regulatory network was tightly associated with pancreatic functions [122, 124, 125] (Additional file 1: Fig. S11h). To regulate SCGN, cRegulon revealed that 6 common REs were used by FOAX2 and ISX, for cooperative regulation (Additional file 1: Fig. S11i).

### cRegulon clarifies cell state transition in RA-induced mEB differentiation

**Fig. 6:**
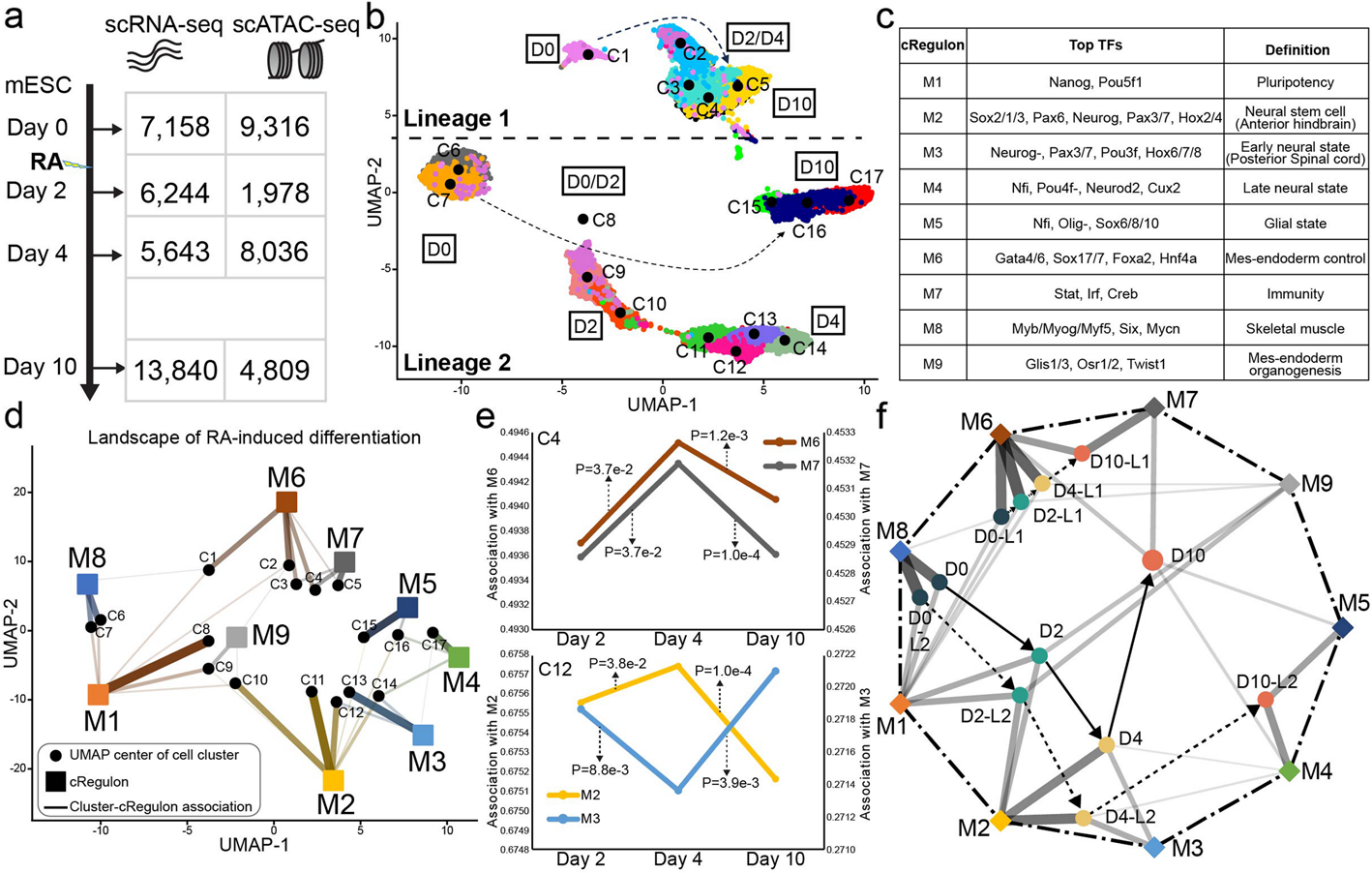
cRegulon reveals landscape of cell state transition in RA-induced mEB differentiation. **a** Single-cell data profiling for RA-induced mEB differentiation. The numbers in the table are the number of cells at each time point. **b** UMAP of scRNA-seq data shows 17 cell clusters across four time points, which can form two developmental lineages. The time point label around cell cluster is based on the proportion of four time points in Additional file 2: Table S8. **c** Nine cRegulons are identified from 17 cell clusters of RA-induced mEB differentiation. **d** The association plot shows the relationship between 9 cRegulons and 17 cell clusters. The thickness of lines shows the association score. Only cell type-cRegulon association scores no less than 0.1 are displayed. **e** The change cRegulon association of C4 and C12, two time-point-shared cell clusters. **f** The cRegulon annotations of four time points and two lineages (Lineage 1 and Lineage 2 defined in **b**). The thickness of solid lines shows the cRegulon annotation score

To see if our method can elucidate the dynamics of regulatory units throughout cellular state transitions, we generated a temporal series of single-cell RNA-seq and single-cell ATAC-seq data. Our initial step involved deriving mEB from mouse embryonic stem cell, followed by their induction into diverse lineages via treatment by retinoic acid (RA) [52, 129]. Over the course of differentiation, we gathered cells or nuclei to compile high-quality datasets for both scRNA-seq and scATAC-seq at distinct time intervals—namely, days 0, 2, 4, and 10 (Fig. 6a, Additional file 2: Table S6). This dataset contained 32,885 cells from scRNA-seq and 24,139 cells from scATAC-seq. Subsequent preprocessing and clustering procedures (Methods) yielded 17 distinct cell clusters (C1-C17) which can be divided into two lineages (Fig. 6b). Lineage 1 (C1-C5) exhibited markers indicative of mesodermal and endodermal development, including Gata4/6, Foxa2, Hnf4a, Sox7/17. Conversely, lineage 2 (C6-C17) was a neural lineage with an enrichment of numerous neural markers such as Pax6/3/7, Neurog1/2, Pou3f2, and Ascl1 (Additional file 2: Table S7). Employing our model on this temporal dataset led to the identification of 9 cRegulons that underlie the RA-induced mEB differentiation.

These 9 cRegulons (termed M1-M9) captured the fundamental functionalities inherent in RA-induced mEB differentiation (Fig. 6c). Our criterion for annotating the cRegulons involved the utilization of top TFs within TF modules, coupled with functional enrichment analyses of both TFs and TGs (Additional file 2: Table S8). For instance, M1 was a pluripotency-related module that contained stem cell markers such as Nanog and Pou5f1 [87]. M2-M5 were modules linked to neural states: M2 indicated a neural stem state marked by Sox2/1/3 [130] and Pax6 [131]; M3 indicated an early neural state hosting proneural genes Neurog1/2 [132] and neural crest markers Pax3/7 [133]; M4 signified a late neural state, expressing Nfi family for cell cycle exiting [134], Pou4f1/2 [135] for mature neurons, Cux2 for cell projection [136]; M5 exhibited enrichment in neuroglial functions, with Olig1/2 [137] and Sox6/8/10 [138] as the key associated TFs. It is noteworthy that many Hox genes were found in M2 and M3, with Hox2-Hox4 and Hox6-Hox9 included in M2 and M3 respectively (Additional file 2: Table S8), indicating that these modules may function in hindbrain development and spinal cord development, respectively [139]. M6 appeared to function in the development of mesodermal and endodermal lineages, featuring several TFs (Gata4/6, Sox7/17, Foxa2, and Hnf4a) crucial for the specification of these lineages. Meanwhile, M7 contained the Stat family and Irf family of regulators, indicative of immune functionalities. M8, on the other hand, demonstrated enrichment in skeletal muscle functions and included TFs such as Myb, Myog, and Myf5. Lastly, as indicated by Glis1/3, Osr1/2, and Twist1 expressions [140–142], M9 was involved in embryonic mes-endoderm organogenesis, including limb, pituitary gland, gonad, and liver. These 9 cRegulons collectively covered functionalities attributed to both ectodermal neuron development and meso-endoderm development, consistent with the lineage differentiation patterns revealed in clustering analysis (Fig. 6b, Additional file 2: Table S7).

The association between the 9 cRegulons and 17 cell clusters effectively depicted the panorama of cellular state transitions (Fig. 6d, Additional file 1: Fig. S12a). The cRegulons characterized time-specific cell clusters, shedding lights on their temporal functional states. For example, the cells of C7, exclusively present on day 0, were annotated by M1 and M8, indicating they were differentiated into muscular state without leukemia inhibitory factor (LIF) [143] while still retaining some self-renewal ability. Similarly, about 98% of C10 cells observed on day 2 were annotated by M2, M1, and M9, portraying neural stem state and pluripotency functions. C13, exclusive to day 4, found its annotation in the early neural state through M3 and M2. C16, a subpopulation of day 10, was predominantly associated with M4/5, signifying the identity of mature neural and glial state. The cRegulon annotations also reflected the delicate evolution of time-lasting cell clusters across different time points. For example, C4 existed in day 2, day 4, and day 20 and was associated with M6 (mes-endoderm development) and M7 (immunity). The annotation analysis of C4 cells across various days (Methods) revealed an increasing association with M6 (meso-endoderm development) from day 2 to day 4 and a subsequent decreasing association from day 4 to day 10, mirroring the annotations of M7 (Fig. 6e). The permutation test (Methods) showed that the association changes across adjacent time points were significant. On the other hand, C12, an early neural cluster, showed an association with neural stem state (M2) and early neural state (M3), demonstrating an increase followed by a decrease in association with neural stem state and an opposite pattern with its early neural state (Fig. 6e). The association changes were also significant based on permutation test.

The application of cRegulon annotations to explore the functional transition across time points revealed intriguing insights. We annotated the two lineages each time point with 9 cRegulons and show their association in the association plot (Methods, Fig. 6f). Day 0 exhibited associations with M1 and M8, signifying that in the absence of LIF, mESC had already been differentiated toward the muscle direction, which was reported to be inhibited by LIF [143]. Subsequent days showcased enriched pluripotency (M1) and neural stem state (M2) at day 2, progressing into early neural state (M3) by day 4. By day 10, the system showed mature states, encompassing glial state (M4), late neural state (M5), meso-endoderm development (M6), and immune functions (M7). Finally, we studied the cRegulon association landscape of two lineages (Fig. 6f). The annotations suggested that on day 0, before RA-treatment, the cells in the EB were already divided into the progenitors of two lineages of different developmental paths, which was consistent with existing findings that that LIF removal causes mESCs to exit their pluripotent state and differentiate into more restricted precursors [144–146]. Subsequently, lineage 1 demonstrated a diminishing identification with meso-endoderm development (M6) and a concurrent increase in identity with immunity (M7). In contrast, lineage 2, predominantly a neural lineage, sequentially evolved through pluripotent (M1), neural stem (M2), early neural (M3), late neural (M4), and glial (M5) states.

### cRegulons from RA time-series data provide annotation of time course dataset for mouse fetal brain development

We hypothesized that if our method identified regulatory units of time-series process, the inferred cRegulons should be well used to annotate similar and independent time-series dataset. For our RA dataset, the temporal properties of derived cRegulons should be reused by new dataset. To validate this hypothesis, we used the inferred cRegulons to annotate an in-vivo time-series dataset for mouse fetal brain development. We obtained MISAR-seq data of mouse fetal brain [147], encompassing both scRNA-seq and scATAC-seq modalities. This dataset comprised four time points (E11_0, E13_5, E15_5, E18_5) and we annotated each time points with 9 cRegulons from RA data (Methods).

**Fig. 7:**
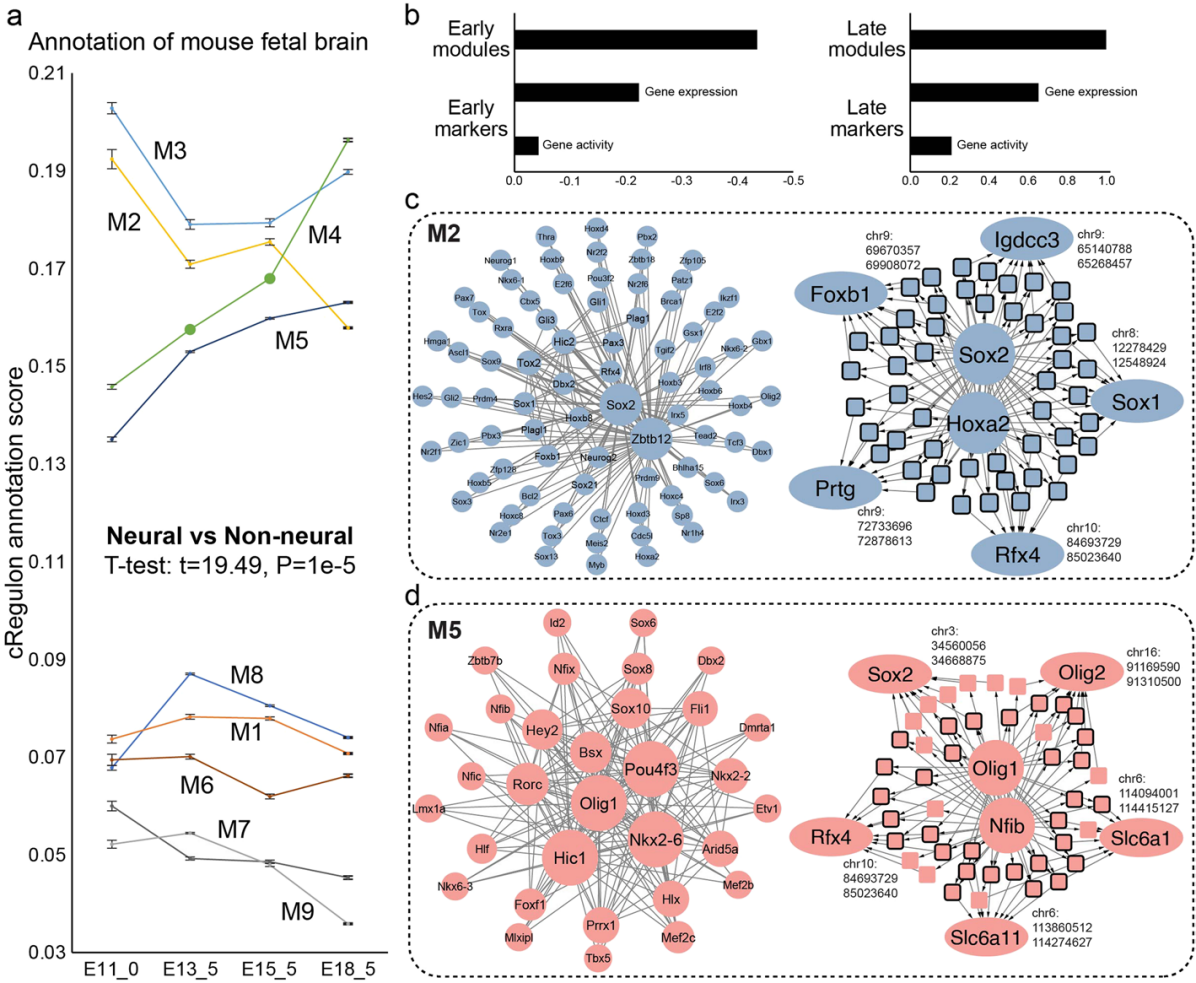
cRegulons inferred from RA-induced mEB differentiation can annotate the temporal states of in-vivo time-series mouse developmental brain data. **a** cRegulon annotation scores of four time points of mouse fetal brain. **b** Comparison of consistency with time points. We compare early modules with early markers (left) and compare late modules with late markers (right). **c** Example of early neural stem state: M2, including TF module (left) and combinatorial regulatory network of Sox2 and Hoxa2. **d** Example of late neural glial state: M5, including TF module (left) and combinatorial regulatory network of Olig1 and Nfib

Notably, we observed a pronounced association of the mouse fetal brain with neural cRegulons (M2–M5), contrasting with the smaller association with non-neural cRegulons (M1 and M6–M9) (Fig. 7a), a finding consistent with the context’s neural characteristics. Furthermore, early neural cRegulons (M2 and M3), particularly M2, exhibited higher association scores at E11_0 and it gradually diminished at later stages. Conversely, late neural states (M4 and M5) displayed an increasing association pattern from E11_0 to E18_5. This temporal shift, characterized by the dominance of early neural states in the initial stages and the prevalence of late neural states in later stages, concurred with the expected temporal dynamics of mouse fetal brain development. Then we compared our temporal annotation ability with another independent annotation by pre-defined maker genes, which have been used to manually annotate cell clusters in mouse brain atlas [148]. Based on the developmental stage of cell cluster in mouse brain atlas that markers belonged to, we classified them into early markers of clusters prior to 13.5 days and late markers of clusters later than 13.5 days. Marker genes’ annotation to time points of mouse fetal brain was their gene expression values or gene activity value. We utilized PCC between annotation scores and time point labels (E11_0, E13_5, E15_5, E18_5) as consistency with temporal states to compare cRegulon and marker genes. For early markers and early cRegulons, we observed cRegulon displayed higher inverse correlation (− 0.43) with time points than early markers (gene expression − 0.22 and gene activity − 0.04) (Fig. 7b, left). On the other hand, late cRegulons had 0.99 PCC with time points, while late markers had 0.65 PCC for gene expression and 0.21 PCC for gene activity (Fig. 7b, right). This comparison demonstrated cRegulon’s better ability to reveal temporal states.

Our early and late cRegulons offered more temporal functional enrichment than markers. We first compared M2 with early markers to evaluate their ability in delineating early neural functions. Functional enrichment analysis of M2’s top 20 TFs and top 200 TGs revealed many neural GO terms, such as “forebrain development,” “neuron differentiation,” and “neuron fate commitment.” M2 was also enriched in “neurogenesis,” indicative of early neural development (Additional file 2: Table S9). We compared cRegulon TFs with early markers and early SCENIC TFs, early ChromVAR TFs, and early SCENIC + TFs inferred from E11.0 and E13.5. We found that early markers and early TFs from other methods could reveal neural functions and cell proliferation functions, but with much lower fold enrichment compared to cRegulon (Additional file 2: Table S9). Then we used M5 to compare the ability to reveal late neural functions. M5 displayed associations with glial functions (“oligodendrocyte differentiation,” “glial cell fate specification”), “cell maturation” and “cell quiescence” (Additional file 2: Table S10), all closely related to cell maturation and glial cells. Conversely, late markers and late TFs from other methods were only enriched in neural pathways, but the maturation functions were lost (Additional file 2: Table S10). These findings underscore the superior precision of cRegulon in elucidating neural temporal functions compared to marker genes.

The cRegulon framework enabled the elucidation of how TF combinations regulate both the early (M2) and late (M5) neural states. The initiation of the neural lineage through M2 involved the acquisition of neural functions while simultaneously maintaining the cell cycle. The TF module within M2 encompassed combinations among Sox2, Zbtb12, Rfx4, and Hox genes (Fig. 7c, left). The co-regulated TGs of these TF combinations exhibited associations with neural stem cells and early neurons. For instance, the prominent co-regulated TGs of Sox2 and Hoxa2 included Sox1 [149] and Rfx4 [150] (Fig. 7c, right). The co-bound REs of TF combinations displayed consistent temporal changes in conjunction with TGs (Additional file 1: Fig. S12b). Additionally, M5 governed the late glial state, prominently featuring TF combinations of Olig1, Pou4f3, Sox10, Sox8, and the Nfi family (Fig. 7d, left). Co-regulated TGs of Olig1 and Nfib were intricately involved in neuroglia development, encompassing entities such as Olig2, Slc6a1 [151], Slc6a11 [152], and Sox2 [153] (Fig. 7d, right). Collaboratively targeting Slc6a1, Olig1, and Nfib utilized 8 shared REs, revealing specific accessibility on day 10 (Additional file 1: Fig. S12c). We also analyzed the interplay between early and late neural cRegulons (Methods, Additional file 1: Fig. S12d). As expected, that two early neural states (M2 and M3) exhibited closer associations, as did the two late neural states (M4 and M5). Between early and late neural states, M2 demonstrated a closer relation to M5, while M3 exhibited a closer connection to M4. This observation implied that the two late neural cRegulons were likely descendants of early neural cRegulons, actively contributing to functionalities in later time points.

In essence, our model of cRegulons provided a holistic picture of the landscape of cell state transitions during RA-induced mEB differentiation (Additional file 1: Fig. S12e). Following the RA stimulus, the embryonic body in its pluripotency state (M1) underwent induction into the neural lineage, regulated initially by M2 and M3 at early stages, and later by M4 and M5. Additionally, the mes-endoderm lineage was also present in this system, primarily governed by M6 and M7. Our comprehensive cRegulon analysis, spanning temporal points, and cell lineages, elucidated the combinatorial control by distinct TF modules (Additional file 1: Fig. S12e), that orchestrated the transition from immature to mature states in two developmental lineages.

## Discussion

Although debatable [154], the Waddington landscape has been a good metaphor to describe cell type development and transition. However, the quantification of cell type landscape is hindered by the shortage of cell-type-specific data and effective methodology. Promisingly, recent advances in single-cell technologies provide more and more cell type resources, which enables more sophisticated gene regulatory analysis. Gene regulatory networks are generally considered to be cell type-specific and are hypothesized to exhibit modular organization, as suggested by several studies [33, 34]. There are many methods to infer the GRN of a cell type using data from that cell type, as well as to extract cell type-specific modules from the corresponding cell type-specific GRN. However, the regulatory modules inferred by such existing methods do not possess the property of being “universal” or “reusable,” which is believed to be an essential character of many modules whose functions are important for multiple cell types [155–157]. Currently, there are no available tools for the inference of such reusable modules as the underlying transcriptional drivers of cell type/state directly from single cell data. In this paper, we present a method for this inference based on the joint analysis of expression and accessibility data from multiple cell types. We believe that gene regulatory analysis must go beyond the compilation of cell type-specific GRNs corresponding to hundreds of cell types. To achieve biological understanding, we will need tools to discover the modular building blocks of these GRNs and to characterize the properties of these GRNs in terms of their usage of the underlying building blocks. At the same time, the importance of TF combination in regulatory modules has been revealed by more and more studies. Motivated by these advances, we proposed the combinatorial regulon concept to systematically model combinatorial regulation. We used simulation data to demonstrate that (1) cRegulon could capture the hallmark TFs characterizing various cell types; (2) cRegulon could adeptly define characteristics of cell types; and (3) cRegulon could identify more accurate TF combinations compared to other methods, when evaluated against an imperfect gold standard with limited coverage of TF combinatorial regulation mechanisms and TF representation. Application to large scale atlas dataset revealed the regulatory units orchestrating early human development, both temporally and spatially. To explore the dynamic landscape of cell state transitions, we generated time series scRNA-seq and scATAC-seq data during RA-induced mEB differentiation. This application gave TF modules governing cell state transitions on levels of cell clusters, time points, and cell lineages. Our method represents the first step in this important direction.

Our model can be extended to more settings. In this paper, we need scRNA-seq data and scATAC-seq data in the same context to ensure (1) plenty of cell types and (2) paired expression and chromatin accessibility information for GRN construction. It will be easier to apply our model to single cell multi-omics data from the same cells, which eliminates the bias of alignment of cell clusters from different omics. On the other hand, if we only have scRNA-seq data, we can still identify plenty of cell types. And some tools, such as Ropen [158], BABEL [159], DeepCAGE [160], and DANCE [161] can be used to predict chromatin state of cell types, which can be integrated with scRNA-seq into cell type-specific GRN. Another way is to utilize GRN models that are only dependent on scRNA-seq, such as SCENIC [44]. After having GRN of multiple cell types, cRegulon model can be built naturally. In principle, any data that can be used to construct cell-type-specific GRNs could be integrated into the cRegulon model (e.g., Perturb-seq, ChIP-seq, HiChIP, and Hi-C). However, demonstrating such extensions remains an area for future work.

The current model still has some limitations. Current GRN construction is on cell type level because of the sparsity of data on the single-cell level and difficulty in integrating multi-omics on single cell. However, cell type identification depends on cell clustering, which is inevitably influenced by clustering methods and the number of clusters chosen. On the other hand, it will be better if we can construct GRN on the single-cell level, which will give us unbiased regulatory information (bias can be introduced by merging cells), cell states at any resolution, and more samples for inferring TF modules. This will be pursued in the future. The usage of expression levels to measure the specificity of TFs for each cell type can also be enhanced by summarizing TF activity using TGs or REs, such as Regulon. There is ongoing debate over whether it is more effective to use TF expression or TF activity inferred from regulons, and whether to construct regulons directly from single-cell data or to utilize pre-built regulons from large databases or sorted cells [162]. Another limitation of our current model is that it only considers the common TGs of TFs to assess their combinatorial effects, thus overlooking information about their binding REs and temporal activity. Our model can be improved by incorporating TFs’ shared enrichment in the same REs and their temporal covariation [81]. Lastly, the current model only utilizes single-cell gene expression and chromatin accessibility data. Current single-cell technologies enable more omics data, such as single-cell chromatin interaction, single-cell methylation, and single-cell spatially resolved omics. We hypothesize that integrating single-cell multi-omics from additional modalities may further improve the modeling of combinatorial regulation, though this remains to be systematically tested.

With the concept and model of cRegulon, more tasks can be reshaped by incorporating TF combination. cRegulon model gives a biologically meaningful and low-dimensional representation to cell types, which can be extended to cell level. This representation can be used for any single-cell task, such as data visualization, clustering, trajectory inference, and cell communications. Cell–cell communication may be a promising task by integrating TF modules of cRegulon and ligand-receptor pairs, since there are already evidence showing integrating paired ligand–receptor and TF activities will benefit cell–cell communication prediction [163]. Another important future direction for cRegulon is extending its capabilities beyond descriptive inference toward predictive modeling of gene perturbations. Integrating dynamic modeling or deep learning components with cRegulon’s regulatory architecture into recent large language models such as CellOracle [164], scGPT [165], and Geneformer [166] can enable predictions of cell state transitions or gene expression changes following TF module perturbations. We view this as a promising avenue for future development, which could further enhance the utility of cRegulon in both basic research and translational applications.

## Conclusion

We provide cRegulon, an approach to model TF combinatorial module from single-cell gene expression and chromatin accessibility data. cRegulon can identify distinct TF modules along with their associated REs as well as TGs, which can be regarded as re-usable functional regulatory units that are basic components of cell type-specific GRNs.

## Methods

### Modeling combinatorial regulation with scRNA-seq and scATAC-seq

Although the input of cRegulon model is GRNs of all cell types and we assume GRNs have been well constructed before running cRegulon, we still provide complete steps to do cRegulon analysis (inference of cRegulons and annotating cell types) starting from raw scRNA-seq and scATAC-seq data.

#### Before cRegulon modeling: single cell data preprocessing, clustering, and GRN construction in each cell cluster

There are existing pipelines for single-cell data preprocessing and clustering. We can choose some standard tools, such Seurat [82], to preprocess data, including data normalization, identification of integration anchors, and dataset integration to effectively mitigate batch effects across samples. Existing tools can also be used to identify cell clusters, such as Seurat and CoupledNMF [83]. After clustering, an important step is to align the cell clusters of scRNA-seq and scATAC-seq data. Several established tools can be applied to accomplish this task. For instance, the “TransferData” function utilizes Canonical Correlation Analysis (CCA) to transfer cell cluster labels from scRNA-seq to scATAC-seq [82]. NNLS employs non-negative least-squares regression to predict scATAC-seq data based on scRNA-seq data and determine their correspondence [167]. CoupledNMF can simultaneously perform cell clustering and align cell clusters through multi-objective optimization.

After determining the cell clusters and their correspondence, we will obtain $K$ cell clusters with both scRNA-seq and scATAC-seq data on the cluster level. For each cell cluster $k$, there will be one gene expression read count matrix ${E}^{k}$ of $N$ genes in ${c}_{1}$ cells from scRNA-seq data and one peak openness read count matrix ${O}^{k}$ of $P$ peaks in ${c}_{2}$ cells from scATAC-seq data. Then we construct a regulatory network with PECA2 model [52] for each cell cluster using its pseudo-bulk gene expression and pseudo-bulk chromatin accessibility data as input. The “pseudo-bulk” stratagem is conducted as follows. We first merge all the cells to measure the pseudo-bulk expression ${PE}_{i}^{k}$ of $i$ th gene and pseudo-bulk openness ${PO}_{r}^{k}$ of $r$ th peak in cell cluster $k$:

3 $$\begin{array}{c}{PE}_{i}^{k}=\frac{{\sum }_{c=1}^{{c}_{1}}{E}_{ic}^{k}}{e}\times {10}^{6}\end{array}$$

4 $$\begin{array}{c}{PO}_{r}^{k}=\frac{{\sum }_{c=1}^{{c}_{2}}{O}_{rc}^{k}}{o}\times {10}^{6}\end{array}$$

Here $e$ is the total number of reads for scRNA-seq data in cell cluster $k$ and $o$ is the total number of reads for scATAC-seq data in cell cluster $k$. ${E}_{ic}^{k}$ is the read count of the $i$ th gene in cell $c$ of cell cluster $k$ and ${O}_{rc}^{k}$ is the read count of peak $r$ in cell $c$ of cell cluster $k$. We select accessible peaks of cell cluster $k$ by the threshold of ${PO}_{r}^{k}\ge 2$ as the candidate REs.

We then input the paired pseudo-bulk gene expression and peak openness into the PECA2 model to construct the regulatory network. PECA2 calculates the trans-regulatory score to measure the regulatory strength of TF $i$ on TG $j$. Hypothesizing that TF regulates the downstream TG by binding at REs, PECA2 computes the trans-regulatory score by integrating multiple REs bound by a TF to regulate TG. The prior TF-TG correlation across external public data from ENCODE database is included in the trans-regulatory score. In detail, the trans-regulatory score ${R}_{ij}^{k}$ of $i$ th TF and $j$ th TG in cell cluster $k$ is quantified as

5 $$\begin{array}{c}{R}_{ij}^{k}=\left({\sum }_{r}{B}_{ir}{{PO}_{r}^{k}I}_{rj}\right)\times {2}^{\left|{D}_{ij}\right|}\times \sqrt{{PE}_{i}^{k}{PE}_{j}^{k}}\end{array}$$

Here ${PE}_{i}^{k}$ and ${PE}_{j}^{k}$ are the pseudo-bulk expressions of the $i$ th TF and $j$ th TG in cell cluster $k$. ${B}_{ir}$ is the motif binding strength of $i$ th TF on $r$ th RE, which is defined as the sum of the binding strength of all the binding sites of $i$ th TF on $r$ th RE. ${PO}_{r}^{k}$ is the pseudo-bulk openness for $r$ th RE in cell cluster $k$. ${I}_{rj}$ represents the interaction strength between $r$ th RE and $j$ th TG, which is learned from the PECA model on diverse ENCODE cellular contexts [168, 169]. ${D}_{ij}$ is the expression correlation of $i$ th TF and $j$ th TG across diverse ENCODE samples. The outputs of PECA2 are the TF-TG regulatory strength matrix ${R}^{k}$ and all the TF-REs-TG regulatory triplets for cell cluster $k$.

#### Pairwise TF combinatorial effect calculation in each cell cluster

Then in each cell cluster, we evaluate the combinatorial effect of two TFs based on the regulatory strength matrix. The combinatorial effect considers two aspects of TFs: TFs’ co-regulation effect and TFs’ activity specificity.

Connection specificity index (CSI) measures the degree of two TFs’ specific co-regulation effect in a cell cluster and a high CSI score means two TFs specifically regulate the same group of TGs. Another reason to choose CSI is that it demonstrates higher accuracy compared to other methods for evaluating TF co-regulation effects across multiple cell lines (Additional file 1: Fig. S13). For cell cluster $k$, with the TF-TG regulatory strength matrix ${R}^{k}$ in Eq. (5) as input, for $i$ th TF and $j$ th TF, we have their regulatory strength on $N$ TGs:

6 $$\begin{array}{c}{R}_{i}^{k}=\left({R}_{i1}^{k},{R}_{i2}^{k},\cdots ,{R}_{iN}^{k}\right); {R}_{j}^{k}=\left({R}_{j1}^{k},{R}_{j2}^{k},\cdots ,{R}_{jN}^{k}\right).\end{array}$$

Here ${R}_{ig}^{k}$ and ${R}_{jg}^{k}$ are the trans-regulatory scores of $i$ th TF and $j$ th TF on $g$ th TG. Then the co-regulation effect of these two TFs is calculated by the Pearson correlation of their regulatory strength:

7 $$\begin{array}{c}{PCC}_{ij}^{k}=\frac{{\sum }_{g}\left({R}_{ig}^{k}-{\overline{R} }_{i}^{k}\right)\left({R}_{jg}^{k}-{\overline{R} }_{j}^{k}\right)}{\sqrt{{\sum }_{g}{\left({R}_{ig}^{k}-{\overline{R} }_{i}^{k}\right)}^{2}{\sum }_{g}{\left({R}_{jg}^{k}-{\overline{R} }_{j}^{k}\right)}^{2}}}\end{array}$$

CSI score considers the specificity of TFs’ co-regulation effect to evaluate their combinatorial regulation:

8 $$\begin{array}{c}{CSI}_{ij}^{k}=\frac{\#\left\{l:{PCC}_{il}^{k}\le {PCC}_{ij}^{k}-\varepsilon , {PCC}_{jl}^{k}\le {PCC}_{ij}^{k}-\varepsilon \right\}}{M}\end{array}$$

Here $M$ is the total number of TFs. $\varepsilon$ is constant with a default value of 0.05.

TF’s specific activity is measured by the TF’s expression specificity index (ESI) in each cell cluster. For TF $i$, we use ${\overline{E} }_{i}^{k}$ to denote its averaged expression in cell cluster $k$ and use ${\overline{E} }_{i}^{-k}$ to denote its averaged expression out of cell cluster $k$. Then the ESI score is defined as:

9 $$\begin{array}{c}{ESI}_{i}^{k}=\left[\frac{{\overline{E} }_{i}^{k}}{{\overline{E} }_{i}^{-k}}-0.5\right], where \left[x\right]=\left\{\begin{array}{c}1, x>1\\ x, 0\le x\le 1\\ 0, x<0\end{array}\right.\end{array}$$

Finally, in the given cell cluster, we define the combinatorial effect of $i$ th TF and $j$ th TF as:

10 $$\begin{array}{c}C_{ij}^k={CSI}_{ij}^k\cdot{ESI}_i^k\cdot{ESI}_j^k\end{array}$$

Here a high combinatorial effect of $i$ th TF and $j$ th TF means they simultaneously have significant specific co-regulation effect and specific expression activity, which make them representative for one cell cluster.

#### Optimization model to identify TF combinatorial modules and cRegulons

Suppose we have $K$ cell clusters and we have their combinatorial effect matrix ${C}^{1}$,${C}^{2}$,$\cdots$, ${C}^{K}$ from the above two procedures. Then our next task is to identify TF combinatorial module from combinatorial effect matrix of all the cell clusters, serving as regulatory units formed by TFs’ combination to underpin cell type landscape. Here we use an optimization model for TF module identification, which is formally formulated as follows:

11 $$\begin{array}{c}\underset{X,A}{\text{min}}{\sum }_{k}\left[{\Vert {C}^{k}-{X{A}_{k}X}^{T}\Vert }_{F}^{2}-\mu {\Vert {C}^{k}\circ \left({X{A}_{k}X}^{T}\right)\Vert }_{1}\right]\end{array}$$

$$\begin{array}{c}s.t.X\ge 0;{\sum }_{i}{x}_{il}^{2}\le 1;{\sum }_{l}{x}_{il}\le 1;l=\text{1,2},\cdots L,i=\text{1,2},\cdots M\\ {A}_{k}=diag\left({A}_{k1},{A}_{k2},\cdots ,{A}_{kL}\right);{A}_{k}\ge 0;{\sum }_{l}{A}_{kl}=1;k=\text{1,2},\cdots K\end{array}$$

Here ${C}^{k}\in {\left[\text{0,1}\right]}^{M\times M}$ is the combinatorial effect matrix of cell cluster $k$ in Eq. (10).

This model has two terms: the first term is designed to decompose TF combinatorial effect matrix into consensus TF modules; the second term constrains the detected TF modules to be TF combinations with large CSI scores. The output of this model is matrix $X\in {\left[\text{0,1}\right]}^{M\times L}$ to reveal the combinatorial effect of $M$ TFs in $L$ cRegulons and diagonal matrix ${A}_{k}\in {\left[\text{0,1}\right]}^{L\times L}$ to annotate cell cluster $k$ with $L$ cRegulons.

#### Extraction of TF combinatorial module and associated regulatory network for cRegulons

Next, we use $X$ matrix to obtain TF modules of $L$ cRegulons. Given the TFs’ combinatorial effect ${X}_{l}={\left({X}_{1l}, {X}_{2l},\cdots ,{X}_{Ml}\right)}^{T}$ of the $l$ th cRegulon, we compute the combinatorial effect of $i$ th TF and $j$ th TF in $l$ th cRegulon:

12 $$\begin{array}{c}{CE}_{ij}^{l}={X}_{il}\cdot {X}_{jl}\end{array}$$

We assume the combinatorial effect of TF pairs in the $l$ th cRegulon follows Gamma distribution, which is learned from the empirical distribution of our experiments (Additional file 1: Fig. S14). We use threshold *P*-value $\le 0.05$ to select TF pairs for $l$ th cRegulon and the significant TF pairs form the representative TF module of $l$ th cRegulon.

Then we measure each TF-TG pair’s regulation strength in $l$ th cRegulon and cell cluster $k$ with ${X}_{l}\left({X}_{l}^{T}{R}^{k}\right)$. We obtain the TGs of TFs in the TF module and approximate the distribution of these TF-TG pairs’ regulation strength by Gaussian distribution. We set the threshold of *P*-value 0.01 to select TF-TG pairs in the regulatory network. Then we obtain the REs of each selected TF-TG pair from the regulatory network. The TFs in TF module, REs and TGs form the regulatory sub-network of $l$ th cRegulon in cell cluster $k$.

#### Annotating cell cluster with cRegulons

Finally, our model output diagonal matrix ${A}_{k}\in {\left[\text{0,1}\right]}^{L\times L}$ for each cell cluster $k$, which is the annotation coefficients and allows us to demonstrate the cell type landscape. Formally, the property of cell cluster $k$ is combinatorically explained by $L$ cRegulons and the association weights are:

13 $$\begin{array}{c}{A}_{k}=diag\left({A}_{k1},{A}_{k2},\cdots ,{A}_{kL}\right)\end{array}$$

where ${A}_{kl}$ is the association score between $k$ th cell cluster and $l$ th cRegulon. To select relevant cRegulons for each cell cluster, we set the threshold to be 0.01 in all the experiments. To select strong associations between cRegulons and cell cluster, we can set the threshold to be 0.1. The $A$ matrix is used to derive association plot (Figs. 3a, 4b, 6d).

We can also derive the cRegulon annotation on cell level with our annotation matrix above. For a cell in cell cluster (either within or outside our dataset), we have its expression vector of $N$ TGs $e=\left({e}_{1},{e}_{2},\cdots ,{e}_{N}\right)$ and the TF-TG TRS matrix $R$ of this cell cluster. Then we use the following formula to annotate this cell with $L$ cRegulons:

14 $$\begin{array}{c}e\cdot {R}^{T}\cdot X\end{array}$$

where $X$ is TF combinatorial matrix from our model. This strategy is used in the annotation of excitatory neurons (Fig. 4f) and beta subtypes (Fig. 4a) in the human fetal atlas application, and in annotation of mouse fetal brain (Fig. 7a) in RA data application.

We can annotate a big cell group that is composed of several cell clusters, such as cell group on sample, organ, and time point level. Given a cell group that is composed of our $K$ cell clusters, the proportion of cell clusters is $p=\left({p}_{1},{p}_{2},\cdots ,{p}_{K}\right)$. Then we use the following formula to annotate this cell group with $L$ cRegulons:

15 $$\begin{array}{c}p\cdot A={\sum }_{k}{p}_{k}{A}_{k}\end{array}$$

This strategy is used in annotating organs in the human fetal atlas application (Additional file 1: Fig. S10b), annotating time points and lineages in RA data application (Fig. 6f).

#### Model initiation, parameter selection, and optimization algorithm

For initiation of our optimization model, we first compute the average of K combinatorial effect matrix: ${C}^{0}=\left({\sum }_{k}{C}^{k}\right)/K$. Then $X$ is initiated by solving a NMF problem: ${C}^{0}={X}^{0}{X}^{0T}$, and ${A}_{k}^{0}$ is set as $diag\left(1/L,1/L,\cdots ,1/L\right)$ for each cell cluster.

The hyper-parameter $\mu$ can be determined by the initiation matrixes:

16 $$\begin{array}{c}\mu =\frac{{\sum }_{k}\left({\Vert {C}^{k}-{X}^{0}{A}_{k}^{0}{X}^{0T}\Vert }_{F}^{2}\right)}{{\sum }_{k}\left({C}^{k}\circ \left({X}^{0}{A}_{k}^{0}{X}^{0T}\right)\right)}\end{array}$$

The hyper-parameter $L$ is the number of cRegulons. $L$ can be determined by elbow rules based on the final loss of our model. First, we try different $L$ to solve the optimization problem and obtain the loss value after convergence. Then we choose the $L$ to be the value when the final losses are not decreased dramatically anymore. This strategy determines the number of cRegulons to be 7 in cell line experiment, 25 in the human fetal atlas application, and 9 in the RA application (Additional file 1: Fig. S15).

Starting from the initiation matrices and hyper-parameters, the multiplicative update algorithm is used to solve the optimization problem of the cRegulon model. We adopt the following update roles:

17 $$\begin{array}{c}X\leftarrow X\cdot \frac{\left(4+{2\mu }_{1}\right)\left(\sum_{k=1}^{K}{C}^{k}X{A}_{k}\right)}{\sum_{k=1}^{K}4{X{A}_{k}X}^{T}X{A}_{k}}\end{array}$$

18 $$\begin{array}{c}{A}_{k}\leftarrow {A}_{k}\cdot \frac{{\left(1+{\mu }_{1}\right)X}^{T}{C}^{k}X}{{X}^{T}X{A}_{k}{X}^{T}X}\end{array}$$

The algorithm will be stopped when the relative error is less than 0.0001.

### Association plot to show the relationship between cRegulons and cell types

We plot cRegulons and cell types into one figure to show their association and cell type landscape more vividly and effectively. Our association plot is based on the hypothesis that the position of cell types is linear combination of the position of cRegulons, and the combination coefficients are given by $A\in {I}^{K\times L}$ matrix of cRegulon output. Formally, the 2-dimensional coordinates of $K$ cell types are $U\in {R}^{K\times 2}$ and the 2-dimensional coordinates of $L$ cRegulons are ${U}^{\prime}\in {R}^{L\times 2}$. Then their linear relationship is derived by:

19 $$\begin{array}{c}U=A{U}^{\prime}\end{array}$$

Two types of association plot are used in our paper. The first association plot is used by Fig. 6f. The coordinates of cRegulons ${U}^{\prime}$ are given by some methods or manual designation. Then the coordinates of cell types will be computed by Eq. (19). The second association plot is represented by Figs. 3a, 4b, and 6d, which start from the coordinates of cell types. The coordinates of cell types $U$ are given by some dimension reduction tools, such as UMAP or t-SNE. Then the coordinates of cRegulons are:

20 $$\begin{array}{c}{U}^{\prime}={A}^{\dag}U\end{array}$$

where ${A}^{\dag}$ is the pseudo reverse matrix of $A$. After we have the coordinates of cRegulons and cell types, we can plot them into one figure.

### Four baseline methods and four naïve methods for identification of cell type-specific TFs and TF pairs

There are four baseline methods for identification of cell type-specific TFs and TF pairs. We first construct three methods with the TF module identification pipeline described in Suo et al. [80]: (1) SCENIC based pipeline: first, we use SCENIC to infer regulons and evaluate TF Regulons’ activity in each cell from scRNA-seq data. Second, the Pearson correlation coefficient (PCC) of TF activity scores is computed for each pair of TFs. Then we transfer the PCC score to CSI scores for each pair of TFs. Within the TF-TF CSI matrix, we use the same procedure as in cRegulon to hypothesize the CSI score of all TF pairs followed Gamma distribution and use threshold of *P*-value $\le 0.05$ to define TF pairs based on SCENIC. (2) ChromVAR based pipeline: first, from scATAC-seq data, ChromVAR uses motif binding to decide TF binding peaks and evaluate TF’s activity in each cell by summing and normalizing the read count of TF’s binding peak, which will give a TF by cell activity matrix. Then we use the same pipeline as SCENIC to define TF pairs based on ChromVAR. (3) SCENIC + based pipeline: from scRNA-seq and scATAC-seq, SCENIC + identifies TFs and evaluates their activity score in each cell, which gives a TF by cell activity matrix. Then we use the same pipeline as SCENIC to define TF pairs based on SCENIC +. The fourth baseline method directly uses the TF-TF regulatory strength, without considering TGs: (4). TF mutual regulation: we make pseudo-bulk data of K562 from scRNA-seq and scATAC-seq and input them into PECA2 model to construct K562’s TF-TG regulatory network. Then we follow the pipeline in Zeng et al. [97] for detecting dense TF network, which gives TF pairs based on TF mutual regulation.

We construct four naïve methods by combining SCENIC TFs, ChromVAR TFs, SCENIC + TFs, and GRN TFs with motif similarity: (1) “SCENIC + motif”: based on scRNA-seq data, “SCENIC + motif” first uses SCENIC to identifies TFs, then compute the motif similarity among these TFs, finally a threshold can select TF combinations. (2) “ChromVAR + motif”: with scATAC-seq data, “ChromVAR + motif” uses ChromVAR to identifies TFs and leverages motif similarity to select TF pairs. (3) “SCENIC + + motif”: with scRNA-seq and scATAC-seq data, “SCENIC + + motif” uses SCENIC + to identifies TFs and leverages motif similarity to select TF pairs. (4) “GRN + motif”: with both scRNA-seq and scATAC-seq data, “GRN + motif” uses PECA2 to infer GRN and selects TFs in high-scored TF-REs-TGs triplets with *P*-value ≤ 1e − 3. We collect motifs from JASPAR, TRANSFAC, UniPROBE, and Taipale. To compute motif similarity, we use the motifSimilarity function from the PWMenrich R package, which calculates the similarity between position weight matrices (PWMs). Since a single TF may be associated with multiple motifs, we first compute the similarity between all pairs of motifs associated with two TFs. Then, we defined the TF-TF similarity as the average of the pairwise motif similarities between their respective motifs. The high motif similarity of these TFs helps identify TF pairs.

### In silico simulation of cell types with different weights of TF modules

We simulate a dataset to evaluate the ability of identifying TF modules (Fig. 2)a. In this simulation, we manually create two TF modules with one shared TF and simulated GRNs for three cell types, each characterized by different weights of the two TF modules.

#### Creating true modular GRN

We manually created a simple and ideal modular GRN with two distinct modules, which serves as our gold standard (Additional file 1: Fig. S2a, b). This GRN consists of 5 TFs and 10 TGs: TF1, TF2, and TF3 co-regulate TG1-TG5 (Module 1, M1), while TF3, TF4, and TF5 co-regulate TG6-TG10 (Module 2, M2). This GRN is considered ideal because the module-specific TFs did not regulate TGs across modules; specifically, TF1 and TF2 do not regulate TG6-TG10, and TF4 and TF5 do not regulate TG1-TG5.

#### Simulating GRNs of three cell types

To simulate cell types associated with different weights of TF modules, we begin by simulating a GRN that includes both M1 and M2, based on the ideal GRN shown in Additional file 1: Fig. S2a, b. The simulation follows this principle: if a TF and a TG are within the same module (either M1 or M2), they are more likely to exhibit a high regulatory strength (A high probability of strong regulatory strength and a low probability of weak regulatory strength); conversely, if a TF and a TG belong to different modules, they are more likely to exhibit a low regulatory strength (A high probability of weak regulatory strength and a low probability of strong regulatory strength). The algorithm for this simulation is as follows:

**Table:** 

For TF-i in [TF1, TF2, TF3, TF4, TF5]:
For TG-j in [TG1, TG2, TG3, TG4, TG5, TG6, TG7, TG8, TG9, TG10]:
If TF and TG **is in same** module (M1 or M2):
Then with P = 0.8${R}_{ij}\sim U\left(\text{0.8,1}\right)$and with P = 0.2${R}_{ij}\sim U\left(\text{0,0.2}\right)$
If TF and TG **is in different** modules:
Then with P = 0.8${R}_{ij}\sim U\left(0, 0.2\right)$and with P = 0.2${R}_{ij}\sim U\left(\text{0.8,1}\right)$

This algorithm will generate the simulated GRN (Additional file 1: Fig. S2c) and the corresponding adjacency matrix which we call the ${R}^{*}$ matrix (Additional file 1: Fig. S2d) that incorporates both M1 and M2.

Next, we simulate the “observed R matrix” for three cell types with different “strengths” (or weights) of the two modules. The strengths of the TF modules in these three cell types are listed in Additional file 1: Fig. S2d. Specifically, to obtain the observed $R$ matrix of a cell type, each entry of the above ${R}^{*}$ matrix is either preserved or perturbed, and the chance of preservation is increased if the corresponding TF-TG relation is included in a module with high strength in the cell type. For instance, SimC1 has a strength of 0.8 for M1, meaning that the regulatory strength within M1 (as shown in Additional file 1: Fig. S2d) will be perturbed (randomly assigned values) with a probability of 0.2. Conversely, SimC1 has a strength of 0.2 for M2, indicating that M2 will be perturbed with a probability of 0.8. The following is the algorithm used to generate cell types with specified module strengths:

**Table:** 

Given a cell type in which strength of M1 is${s}_{1}$and the strength of M2 is${s}_{2}$
For$${TF}_{i}-{TG}_{j}$$in M1:
With P=${s}_{1}$ ${R}_{ij}$is preserved
with P = 1-$${s}_{1}$$ $${R}_{ij}\sim U\left(\text{0,1}\right)$$
For$${TF}_{i}-{TG}_{j}$$in M2:
With P=${s}_{2}$ ${R}_{ij}$is preserved
with P = 1-$${s}_{2}$$ $${R}_{ij}\sim U\left(\text{0,1}\right)$$

As expected, the smaller the strength of a module, the higher the probability that the regulatory strength is assigned randomly. This random assignment disrupts the modularity of the module. In our study, we use the strengths listed in Additional file 1: Fig. S2d to simulate three cell types. As shown, the modular GRN of M1 in SimC1 is well-preserved, while M2 is compromised (Additional file 1: Fig. S2e). SimC2 maintain both M1 and M2 clearly (Additional file 1: Fig. S2f). In contrast, SimC3 exhibit a clear M2 but an ambiguous M1 (Additional file 1: Fig. S2g).

#### Simulating single-cell RNA-seq data of three cell types

Once we generate the GRN for each cell type, we utilize existing tools, such as SERGIO [85], to simulate scRNA-seq data. SERGIO uses the GRN adjacency matrix (as shown in FiguresS1e-g) and the production rates for TF regulators in each cell type as inputs. We decide each cell type’s TF production rates according to their TF module composition: we set a TF’s production rate to be 0.6 if it is in the dominant TF module, 0.4 if it is in the non-dominant TF module, and 0.5 if it is shared by two TF modules. We simulate 1000 cells for each cell type. The final output is a count matrix containing 15 genes and 3000 cells in total. Additional file 1: Fig. S2h shows the UMAP of simulated scRNA-seq data of three cell types.

### Validation with gold standard TF combination pairs from ChIP-seq, ChIA-PET, HiChIP, and PPI data

Because the co-regulation of TFs can be physically achieved by DNA co-binding, chromatin loops, and protein–protein interactions, we use TF ChIP-seq data, ChIA-PET data, HiChIP data, and protein–protein interactions to build TF pairs for approximation of gold standard TF combinations.

For TF ChIP-seq data, we collect ChIP-seq data of 493 TFs (Additional file 2: Table S11) for K562 from ENCODE. We apply ChIP-GSM [73] model, which designed for TF modules detection, to our collected ChIP-seq data. ChIP-GSM output 172 TF pairs for K562.

For ChIA-PET or Hi-C data, we first call loops and then use the number of loops that connected TFs to define TF pairs. A loop connects two TFs if (1) anchor 1 is bound by TF 1 and anchor 2 is bound by TF 2, or (2) anchor 1 is bound by TF 2 and anchor 2 is bound by TF 1. For K562, we use the collected TF ChIP-seq data to define anchor bound by a TF. Then for any TF pairs, we identify their loops that satisfy the above conditions. Finally, we use the number of loops of TF pairs to define TF combinations. We set the threshold of loop number to be 80 and we obtained 4388 ChIA-PET TF pairs for K562.

For PPI, we collect TF protein–protein interaction of human and mouse from the BioGRID database [69]. Totally, there are 1,058,854 PPIs in human and 902,843 PPIs in mouse.

We construct a combined ground truth by integrating evidence from ChIP-seq, ChIA-PET, and PPI datasets. However, since the number of TF pairs varies greatly across these sources—with ChIP-seq containing the fewest—directly merging all three will cause the ChIP-seq signal to be overwhelmed by the others. To address this, we adopt an equal-sampling strategy to ensure balanced representation. Specifically, we randomly sample $N$ TF pairs from each dataset, resulting in one combined ground truth of $3N$ TF pairs. We then evaluate all metrics—precision, recall, F1-score, and AUPR ratio—on this sample of combined ground truth. This sampling process will be repeated 10,000 times, and the final metrics are reported as the average across all repetitions. Here *N* is set to be the number of TF pairs in ChIP-seq.

To measure the accuracy of identifying TF combination, we define the AUPR ratio as the fold change between the AUPR of a method and that of random guessing: $AUPR ratio=\frac{AUPR}{\#real positive/\#sample}$. For random guessing, the AUPR equals the fraction of positive samples in the dataset.

### Seurat analysis pipeline of single-cell data of RA-induced mEB differentiation

For scRNA-seq data, we input the raw fastq files into 10 × Genomics Cell Ranger 6.1.2 and the reference transcriptome for alignment and annotation is version mm10-2020-A. To combine all filtered count matrices for different replicates, the “cellranger aggr” command is applied with the default depth normalization method. We filter cells with less than 2500 read counts and obtain the gene-cell read count $E$ of $N$ genes in ${c}_{1}$ cells. We obtain UMAP embedding with gene expression matrix $E$ after PCA with “umap” package. For scATAC-seq data, we input the raw fastq files into 10 × Genomics Cell Ranger ATAC 2.0.0 software and the reads are aligned to the reference genome version mm10-2020-A. The data for different replicates for each time point are aggregated with the “cellranger-atac aggr” function by specifying depth normalization. We obtain the peak-cell read count $O$ of $P$ peaks in ${c}_{2}$ cells and obtained UMAP embedding with gene activity matrix $O$ after PCA with “umap” package.

With processed gene expression matrix and peak openness matrix, we can use some integrative methods to detect cell clusters, such as Seurat and CoupledNMF. Seurat first conducts standard clustering analysis on scRNA-seq data. We use “NormalizeData” and “ScaleData” to normalize and scale data and use “FindVariableFeatures” to find the top 3000 most variable genes. Then we run PCA with the “RunPCA” function and find neighbors with “FindNeighbors”. Finally, we use “FindClusters” to identify cell clusters and use “FindMarkers” to get the gene expression markers of each cell cluster. For scATAC-seq, Seurat conducts the integrative analysis with scRNA-seq to transfer labels from scRNA-seq to scATAC-seq. Specifically, the read count matrix of scATAC-seq is used to compute gene activity with “CreateGeneActivityMatrix” and is normalized, scaled with “NormalizeData” and “ScaleData” functions. We then use “FindVariableFeatures” to find the top 3000 most variable genes and we run latent semantic indexing with the “RunLSI” function. Next, we use “FindTransferAnchors” to identify anchors between scATAC-seq and scRNA-seq and we use “TransferData” to transfer cell cluster labels of scRNA-seq to scATAC-seq. Finally, we use the “FindMarkers” function to get the gene activity markers with the input of the gene activity matrix. One important task is to decide the number of clusters. To do this, we try different parameters of resolution (from 0.05 to 2.0 with step 0.05) and we evaluate the clustering effect with the Silhouette index [170], Clustree [171], and ROGUE [172] to determine the optimal one.

### Functional enrichment analysis and comparison

Given a list of genes, we use DAVID to conduct functional enrichment analysis and select three kinds of GO terms: biological pathway (BP), cellular component (CC), and molecular function (MF). We filter out less-informative terms, such as “Positive regulation of transcription from RNA polymerase II promoter,” “Positive regulation of transcription, DNA-templated,” “Regulation of gene expression,” and so on. The gene lists used for analysis or comparison are described as follows.

In analyzing functions of cRegulons, we conduct functional enrichment analysis on top 20 TFs and top 200 TGs and consider them together to determine the functions of cRegulon. The GO results are provided in Additional file 2: Table S3 and Additional file 2: Table S8, while the definitions of cRegulons are provided in Table 1 and Fig. 4c for human fetal atlas and RA dataset, respectively.

In comparing concentration of functional enrichment, we get DEGs for each cell type of human fetal atlas from previous publication [10], use GOSemSim to compute the similarity of each GO term pair, and compute the averaged GO similarity score as the concentration of DEGs for this cell type. Given a cell type, we extract 235 genes (50 TFs and 185 TGs) from all its associated cRegulons, and the constituent TF number and TG number are proportional to their association scores. Then we use the same procedure with GOSemSim to obtain the concentration of cRegulon for this cell type. In four examples to compare functional enrichment with DEGs, we use top 20 TFs and top 200 TGs from cRegulon and top 220 gene from DEGs for functional enrichment with DAVID.

### The transition score between two cRegulons and differential degree of cRegulon annotation between two cell types

We utilize the change of the combinatorial effect of TF modules to reveal the transition of cRegulons. Formally, let ${M}_{i}$ and ${M}_{j}$ be the top TFs of $i$ th TF module and $j$ th TF module. We first define the forward score ${FS}_{ij}$ to evaluate the ${M}_{i}$ TFs’ combinatorial effect in the $j$ th TF module:

21 $$\begin{array}{c}{FS}_{ij}=\frac{1}{\left|{M}_{i}\right|}{\sum }_{l\in {M}_{i}}{X}_{lj}\end{array}$$

And we define the backward score ${BS}_{ij}$ to evaluate the ${M}_{j}$ TFs’ combinatorial effect in the $i$ th TF module:

22 $$\begin{array}{c}{BS}_{ij}=\frac{1}{\left|{M}_{j}\right|}{\sum }_{l\in {M}_{j}}{X}_{li}\end{array}$$

Here ${X}_{li}$ is the combinatorial effect value of TF $l$ in $i$ th TF module and ${X}_{lj}$ is the combinatorial effect value of TF $l$ in $j$ th TF module. $\left|{M}_{i}\right|$ and $\left|{M}_{j}\right|$ were numbers of ${M}_{i}$ TFs and ${M}_{j}$ TFs.

Finally, we combine the forward score and backward score to be the transition score from $i$ th TF module to $j$ th TF module:

23 $$\begin{array}{c}{TS}_{ij}=\lambda {FS}_{ij}+\mu {BS}_{ij}\end{array}$$

Here we set $\lambda$ to 0.5 and $\mu$ to 0.5.

We also analyze the differential degree of cRegulon annotation between cell types. Let ${A}_{1l}$ and ${A}_{2l}$ be the annotation score of cRegulon $l$ for cell type 1 and cell type 2. Then we combine the absolute difference of relative difference to define the differential degree of cRegulon $l$ for cell type 1 and cell type 2:

24 $$\begin{array}{c}{D}_{l}=\left(max\left({A}_{1l},{A}_{2l}\right)-min\left({A}_{1l},{A}_{2l}\right)\right)*\frac{max\left({A}_{1l},{A}_{2l}\right)}{min\left({A}_{1l},{A}_{2l}\right)}\end{array}$$

To evaluate the statistical significance of differential degrees based on cRegulon annotation, we use permutation to construct a background of differential degrees. Taking β subtype annotation as an example, there are 31,069 β cells, including 17,712 beta1 cells and 13,357 beta2 cells. We have used 25 cRegulons to annotate two subtypes and compute their differential degree on each cRegulon (${D}_{1}$, ${D}_{2}$, …, ${D}_{25}$). Our background construction contains the following steps:

- We randomly divide all β cells into a pseudo-beta1 group of 17,712 cells and a pseudo-beta2 group of 13,357 cells.
- With the same procedure, we annotate the pseudo-beta1 cell group and pseudo-beta2 cell group and compute their differential degree on each cRegulon (${PD}_{1}$, $P{D}_{2}$, …, $P{D}_{25}$)
- We repeat (a) and (b) for $N=50$ times, which will give 50 background differential degrees for each cRegulon.
- For each cRegulon, we compute the mean (${\mu }_{i}={\sum }_{t}{PD}_{i}^{t}/N$) and standard deviation (${\sigma }_{i}=\sqrt{{\sum }_{t}\left({PD}_{i}^{t}-{\mu }_{i}\right)^2/\left(N-1\right)}$) from background differential degrees for each cRegulon.
- For each cRegulon, we compute Z-score (${Z}_{i}=\left({D}_{i}-{\mu }_{i}\right)/{\sigma }_{i}\sim N\left(\text{0,1}\right)$) and obtain their *P*-value and FDR-adjusted *P*-value.

Second, to evaluate the significance of the change between time points within a cell cluster, such as time-course annotation of RA cell cluster in Fig. 6e, we also design a permutation test. Suppose we are analyzing a cell cluster, there are ${N}_{1}$ cells from time point ${t}_{1}$ and ${N}_{2}$ cells from time point ${t}_{2}$, and we observed a change $c$ of association scores. Then we randomly choose ${N}_{1}$ cells to annotate, randomly choose ${N}_{2}$ cells to annotate, and compute the change of their association scores. This random sampling was repeated 10,000 times, which gave 10,000 changes (${c}_{1},{c}_{2},{c}_{3},\cdots ,{c}_{10000}$) of association scores. Finally, the *P*-value of change $c$ is: $P=\frac{{\sum }_{i}{I}_{{c}_{i}>c}}{10000}$, where $${I}_{{c}_{i}>c}=\left\{\begin{array}{c}1, {c}_{i}>c\\ 1, {c}_{i}\le c\end{array}\right.$$. The *p*-values of significance of the change between time points within RA cell cluster are shown in Fig. 6e.

### Cell culture and differentiation of RA-induced embryoid bodies from mESC

Mouse embryonic stem cell line R1 was purchased from the American Type Culture Collection (ATCC, SCRC-1036). Upon receiving the R1 cells, we followed the ATCC protocol to expand them from the frozen vial. The cells were initially expanded on a previously irradiated MEF feeder layer. We then subcultured them on 0.1% bovine gelatin-coated tissue culture plates in mESC medium. This medium consisted of Knockout DMEM supplemented with 15% Knockout Serum Replacement, 100 μM nonessential amino acids, 0.5 mM beta-mercaptoethanol, 2 mM GlutaMax, and 100 U/mL Penicillin–Streptomycin with the addition of 1000 U/mL of LIF (ESGRO, Millipore). During cell expansion, individual stocks containing approximately $2\times {10}^{6}$ cells per vial were collected for future experiments. For all experiments, the cells were used between passages 5 and 10. We also regularly tested for mycoplasma contamination, and all tests were negative.

mESCs were differentiated using the hanging drop method [173]. Trypsinized cells were suspended in differentiation medium (mESC medium without LIF) to a concentration of 50,000 cells/ml. Twenty-microliter drops (~ 1000 cells) were then placed on the lid of a bacterial plate and the lid was upside down. After 48 h incubation, embryoid bodies (EBs) formed at the bottom of the drops were collected and placed in the well of a 6-well ultra-low attachment plate with fresh differentiation medium containing 0.5 $\upmu$ M retinoic acid (RA) for up to 10 days, with the medium being changed daily.

### Library preparation and scRNA-seq and scATAC-seq sequencing

For scRNA-seq, we followed 10X Genomics library preparation protocol. The EBs were collected at days 0, 2, 4, and 10. They were first treated with StemPro Accutase Cell Dissociation Reagent (Thermo Fisher) at 37 °C for 10–15 min with pipetting. Single-cell suspension was obtained by passing through 37-$\upmu$ M cell strainer (STEMCELL Technologies) twice. After measuring cell concentration, approximately 1 million cells were centrifuged at 300 rcf for 5 min. The cell pellet was washed once with PBS + 0.04% BSA. The final cell concentration was adjusted to 1000 cell/$\upmu$ L in PBS + 0.04% BSA. The sample was then submitted to Stanford Genomics Service Center (SGSC) for single-cell sorting using 10X Chromium Controller (target cells: 5000 per replicate, total 2 replicates per time point). The scRNA-seq library was generated using Chromium Next GEM Single Cell 3’ Kit v3.1 (10X Genomics, PN-1000268). A detailed summary of the sequencing, mapping, and cells was provided in Additional file 2: Table S6. Our scRNA-seq dataset had a high library complexity, with an average of 11,000–15,000 unique molecular identifiers (UMIs) per cell, which was higher than the scRNA-seq of PBMC dataset (5496 UMIs at https://www.10xgenomics.com/resources/datasets/5-k-peripheral-blood-mononuclear-cells-pbm-cs-from-a-healthy-donor-with-cell-surface-proteins-v-3-chemistry-3-1-standard-3-1-0). We detected an average of approximately 3200–4000 genes per cell for each replicate, compared to 1644 genes for PBMC. These findings indicated that a larger proportion of the transcriptome was captured for RA induction compared to PBMC. Moreover, the total number of reads, mapping ratio, and number of cells detected were comparable to those of PBMC, indicating that the scRNA-seq data has good quality.

For scATAC-seq, we followed 10X Genomics library preparation protocol. The EBs were collected at days 0, 2, 4, and 10. Single-cell suspension was first obtained using the same procedure shown above. After measuring cell concentration, approximately 1 million cells were centrifuged at 300 rcf for 5 min at 4 °C. Cells were lysed by incubating in 100 µL chilled Lysis Buffer for 5 min on ice. After washing with 1 mL chilled Wash Buffer, the nuclei were isolated by centrifuged at 500 rcf for 5 min at 4 °C. The final nuclei concentration was adjusted to 3000 cell/$\upmu$ L in 1X Nuclei Buffer (10X Genomics). The sample was then submitted to Stanford Genomics Service Center (SGSC) for single-cell sorting using 10X Chromium Controller (target cells: 5000 per replicate, total 2 replicates per time point). The scATAC-seq library was generated using Chromium Next GEM Single Cell ATAC Library & Gel Bead Kit (10X Genomics, PN-1000175). A detailed summary of the sequencing, mapping, and cells is provided in Additional file 2: Table S6. Our scATAC-seq dataset generally detected a higher average of ~ 15,000–35,000 median high-quality fragments per cell compared to the PBMC dataset (14,866), except one replicate (D2-1) which had 11,008 fragments per cell. The number of peaks detected per cell in our RA dataset was ~ 129,000–220,000, which was on average higher than the PBMC dataset (144,023 at https://cf.10xgenomics.com/samples/cell-atac/2.0.0/atac_pbmc_10k_nextgem/atac_pbmc_10k_nextgem_web_summary.html). The TSS enrichment score for RA was 8.12–10.48, which was comparable to PBMC (10.55). Furthermore, the total number of reads, mapping ratio, and number of cells detected in our RA dataset were comparable to those of PBMC, indicating that our scATAC-seq data has good quality.

## Supplementary Information

Additional file 1: Text S1: Impact of incorporating more cell types into cRegulon. Text S2: Ablation studies of cRegulon. Text S3: cRegulon is robust to cell clustering. Text S4: cRegulon is robust to the unpaired single cell dataset. Text S5: cRegulon can be extended to study RE combination. Fig S1: An example of Sox2, Oct4 and Nanog to combinatorically regulate pluripotency of mESC. Fig. S2: Process of in silico simulation. Fig. S3: Comparison of p-value thresholding TF pairs. Fig. S4: Comparison with four naive baseline methods. Fig. S5: Simulation study of effect of more cell types. Fig. S6: Ablation study shows the importance of components of cRegulon modeling. Fig. S7: cRegulon is robust to cell cluster number. Fig. S8: cRegulon is well applicable to both paired and unpaired single cell data. Fig. S9: Extension of cRegulon to study RE combination. Fig. S10: Heatmap of association matrix between cell types and cRegulons. Fig. S11: Comparison of Functional enrichment concentration. Fig. S12: Detailed combinatorial regulation of RA dataset revealed by cRegulon. Fig. S13: Justification of choosing CSI. Fig. S14: The empirical distribution of TF pairs’ combinatorial effect. Fig. S15: Choosing optimal number of cRegulons with final loss.

Additional file 2: Table S1: Functional enrichment with TGs of M2 and M6 in simulation experiment. Table S2: The AUPRC values of five methods on four gold standard TF pair sets. Table S3: cRegulons of human fetal atlas. Table S4: Top 20 GO pathways enriched in cRegulon, DEGs, ChromVAR TFs, SCENIC TFs and SCENIC + TFs of hematopoietic stem cell. Table S5: Top 20 GO pathways enriched in cRegulon, DEGs, ChromVAR TFs, SCENIC TFs and SCENIC + TFs of Syncytiotrophoblasts-and-villous-cytotrophoblasts. Table S6: Summary of the sequencing, mapping, and cells for scRNA-seq and scATAC-seq of RA data. Table S7: Marker genes analys with Seurat for 17 cell clusters of RA data. Table S8: cRegulons of RA dataset. Table S9: Top 20 GO pathways enriched in early cRegulon, early markers, early SCENIC TFs, early ChromVAR TFs and early SCENIC + TFs. Table S10: Top 20 GO pathways enriched in late cRegulon, late markers, late SCENIC TFs, late ChromVAR TFs and late SCENIC + TFs. Table S11: The ENCODE accession and download url of TF ChIP-seq and H3K27ac ChIA-PET data of K562.

## Data Availability

The simulation data of K562, GM12878, BJ, and H1-ESC are downloaded from GEO under accession GSE126074 [101]. The simulation data of HMEC (ENCSR000COX, ENCSR860HAA, ENCSR228VNQ), HUVEC (ENCSR000CPA, ENCSR000CPB, ENCSR000COZ, ENCSR000EOQ), and GM23248 (ENCSR510QZW, ENCSR510QZW, ENCSR217TAW) are downloaded from ENCODE [174]. The human fetal atlas dataset is downloaded from https://descartes.brotmanbaty.org/ [10, 11]. The pancreatic beta cell dataset is downloaded from GEO under accession GSE200044 [113]. The mouse fetal brain dataset is downloaded from https://www.biosino.org/node/project/detail/OEP003285 [147]. The raw and processed scRNA-seq and scATAC-seq data of RA-induced mEB differentiation are available at GEO under accession GSE227320 [175]. cRegulon software is freely available at GitHub https://github.com/SUwonglab/cRegulon [176] and Zenono https://doi.org/10.5281/zenodo.15749686 [177] under the GPL-3.0 license.
