# Supplementary material for: Modeling combinatorial regulation from single-cell multi-omics provides regulatory units underpinning cell type landscape using cRegulon
Source: Genome Biol. 2025 Jul 24;26:220. doi: 10.1186/s13059-025-03680-w (PMC12291291; doi:10.1186/s13059-025-03680-w)
Supplement: Supplementary file 1 — Additional file 1: Text S1: Impact of incorporating more cell types into cRegulon. Text S2: Ablation studies of cRegulon. Text S3: cRegulon is robust to cell clustering. Text S4: cRegulon is robust to the unpaired single cell dataset. Text S5: cRegulon can be extended to study RE combination. Fig S1: An example of Sox2, Oct4 and Nanog to combinatorically regulate pluripotency of mESC. Fig. S2: Process of in silico simulation. Fig. S3: Comparison of p-value thresholding TF pairs. Fig. S4: Comparison with four naive baseline methods. Fig. S5: Simulation study of effect of more cell types. Fig. S6: Ablation study shows the importance of components of cRegulon modeling. Fig. S7: cRegulon is robust to cell cluster number. Fig. S8: cRegulon is well applicable to both paired and unpaired single cell data. Fig. S9: Extension of cRegulon to study RE combination. Fig. S10: Heatmap of association matrix between cell types and cRegulons. Fig. S11: Comparison of Functional enrichment concentration. Fig. S12: Detailed combinatorial regulation of RA dataset revealed by cRegulon. Fig. S13: Justification of choosing CSI. Fig. S14: The empirical distribution of TF pairs’ combinatorial effect. Fig. S15: Choosing optimal number of cRegulons with final loss. [file 13059_2025_3680_MOESM1_ESM.pdf]

Modeling combinatorial regulation from single-cell multi-omics provides regulatory units underpinning cell type landscape using cRegulon

Zhanying Feng[1,2#], Xi Chen[2#], Zhana Duren[3], Jingxue Xin[2], Hao Miao[1], Qiuyue Yuan[3], Yong Wang[1,4,5,6*], Wing Hung Wong[2*]

[1]State Key Laboratory of Mathematical Sciences, Academy of Mathematics and Systems Science, Chinese Academy of Sciences, Beijing 100190, China

[2]Department of Statistics, Department of Biomedical Data Science, Bio-X Program, Stanford University, Stanford, CA 94305, USA

[3]Center for Human Genetics and Department of Genetics and Biochemistry, Clemson University, Greenwood, SC 29646, USA

[4]School of Mathematics, University of Chinese Academy of Sciences, Chinese Academy of Sciences, Beijing 100049, China

[5]Center for Excellence in Animal Evolution and Genetics, Chinese Academy of Sciences, Kunming 650223, China

[6]Key Laboratory of Systems Biology, Hangzhou Institute for Advanced Study, University of Chinese Academy of Sciences, Chinese Academy of Sciences, Hangzhou 330106, China

[#]These authors contributed equally to this work

*Corresponding authors: whwong@stanford.edu, ywang@amss.ac.cn

Contents

Supplementary Texts 3

Text S1: Impact of incorporating more cell types into cRegulon 3

Text S2: Ablation studies of cRegulon 3

Text S3: cRegulon is robust to cell clustering 4

Text S4: cRegulon is robust to the unpaired single cell dataset 5

Text S5: cRegulon can be extended to study RE combination 6

Reference 6

Supplementary Figures 8

Fig. S1 8

Fig. S2 9

Fig. S3 10

Fig. S4 11

Fig. S5 12

Fig. S6 13

Fig. S7 14

Fig. S8 15

Fig. S9 15

Fig. S10 16

Fig. S11 17

Fig. S12 18

Fig. S13 19

Fig. S14 20

Fig. S15 21

Supplementary Texts

Text S1: Impact of incorporating more cell types into cRegulon

To evaluate the impact of incorporating more cell types into cRegulon analysis, we expanded our study by including human mammary epithelial cells (HMEC), human umbilical vein endothelial cells (HUVEC), and another fibroblast cell line (GM23248). Application cRegulon to this expanded set of 7 cell lines resulted in the identification of 8 cRegulons. We compared these 8 cRegulons to the 7 cRegulons previously identified from 4 cell lines, by computing the Pearson correlation of their corresponding X vectors. We observed strong correlations between most cRegulons, including M1-M4, M6, and M7, with those from the original 4 cell lines (**Fig. S5b**). The associated cell types of these 6 cRegulons also remained consistent with our earlier findings (**Fig. S5c**), indicating that the information from the initial 4 cell lines was preserved. We found two new cRegulons: M5 and M8, which were different from previous cRegulons (**Fig. S5b**). M5 was specific to GM23248 and M8 was more relevant to HMEC and HUVEC (**Fig. S5c**). M5 was characterized by TFs TBX5 and HIF1A (**Fig. S5d**). TBX5 is known to promote cardiomyogenic differentiation of cardiac fibroblasts [1], while HIF-1 is a critical regulator of extracellular matrix remodeling under hypoxic conditions in fibroblasts [2]. M8 was characterized by BCL6B, SNAI2, and EPAS1 (**Fig. S5e**). The TF BCL6B is implicated as a putative endothelial-specific master regulator [3], SNAI2 induces epithelial-mesenchymal transition [4], and EPAS1 is preferentially expressed in vascular endothelial cells [5]. Thus, the functions of the TFs in the new modules are highly relevant for the new cell lines that were added to the analysis. This suggested that the inclusion of additional cell types can lead to the emergence of new regulatory modules involving TFs relevant to the added cell type. Finally, we further validated the TF pairs of K562 (M1) against combined ground truth, which confirmed that cRegulon still outperformed other methods (**Fig. S5f**). In the original 4-cell-line cRegulons, JUN TFs were more enriched in M1, while FOS TFs were more enriched in M2 (**Fig. S5g,** up). With the expanded 7 cell lines, we observed both JUN and FOS TFs in M2 (**Fig. S5g,** down), which is more consistent with their known collaborations within the AP1 family [6-8]. This enhancement was facilitated by the inclusion of additional cell types. Specifically, in the context of 4 cell lines, only 2 cell types (BJ and K562, 50%) showed combinatorial evidence between JUN and FOS TFs (**Fig. S5h**, up). However, with 7 cell lines, a larger proportion (5 out of 7 cell types, 71%) exhibited JUN-FOS combinatorial evidence (**Fig. S5h**, down), making it easier to identify JUN and FOS TFs within the same TF module. These findings underscored the improvement in cRegulon performance with joint analysis on more diverse cell types.

Text S2: Ablation studies of cRegulon

In our model, there are three critical steps (**Fig. S6a**): (1) integration of scRNA-seq and scATAC-seq to construct the gene regulatory network (GRN); (2) consideration of TFs' common target genes (TGs) and activity specificity to evaluate their combinatorial effects; and (3) identification of TF modules across all cell types as universal and reusable regulatory units. The importance of

integrating gene expression and chromatin accessibility to construct the GRN has already been well validated by our previous PECA2 paper.

To evaluate the necessity of considering TGs, we replaced the CSI score of TFs with the mutual regulatory score of TFs (abbreviated as "NoCSI"), which does not account for their common TGs. To evaluate the necessity of considering TF activity specificity, we removed ESI from combinatorial effect calculation (abbreviated as "NoESI"). Additionally, to assess the importance of inferring TF modules from all cell types, we replaced it with inferring TF modules from only one cell type (K562) (abbreviated as "Single"). For each experiment, we determined K562 specific modules by selecting the TF module with the largest Z-score of association score across different cell types. We used the combined TF pair ground truth from ChIP-seq, ChIA-PET, and PPI and computed AUPR ratio as metric to show the effect of CSI, ESI and multiple cell types.

Fig. S6b showed 7 TF modules identified by "NoCSI", with M2 most specific to K562. **Fig. S6c** showed 7 TF modules identified by "NoESI", with M1 most specific to K562. **Fig. S6d** showed 7 TF modules identified by "Single", with M7 most associated with K562.

Fig. S6e and Fig. S6f showed that all three components are important for the accuracy of cRegulon, since removing CSI, ESI, and "all cell types" decreased the performance of cRegulon.

Since ESI is associated with the number of cell types. Then we evaluate the effect of ESI on both more and less heterogeneous data. We included three more cell line data, including human mammary epithelial cells (HMEC), human umbilical vein endothelial cells (HUVEC), and another fibroblast cell line (GM23248), into analysis. CL4-NoESI meant the experiment on dataset of 4 cell lines, and the model is without ESI. CL7-NoESI is the experiment on dataset of 7 cell lines, and the model is without ESI. Then we computed the relatively decreasing rate of removing ESI on CL4 and CL7 to show the effect of ESI on different levels of heterogeneity. And we found that the relatively decreasing rate is 0.21 on CL4 and 0.37 on CL7 (**Fig. S6g**), which means that ESI may exert more influence on more heterogeneous data.

Text S3: cRegulon is robust to cell clustering

In practice, the numbers of cell clusters can introduce variability and may influence our model. We conducted a robustness analysis using the RA dataset and shows that our method is not highly sensitive to the definition of "cell-cluster" by several metrics. Initially, we used Seurat with different resolutions for cell clustering. Then we used several metrics, including clustering steady branch by Clustree package, cluster distance by Silhouette index, and cluster purity by ROGUE package, to determine that 17 cell clusters at a Seurat resolution of 0.6 was optimal for RA. Then we varied the Seurat resolutions for robustness analysis.

We first set a higher resolution of 0.65 to obtain 18 cell clusters. Then we ran our model with those 18 cell clusters to identify 9 cRegulons. We computed the PCC of the output X vectors of 9 cRegulons from 18 cell clusters and 9 cRegulons from 17 cell clusters to evaluate if the TF modules were greatly influenced by finer cell clusters. From the heatmap (**Fig. S7a**), we could observe there was a good conservation (diagonal elements) between two cluster numbers. Then we used

Hungarian algorithm to identify cRegulon alignment between them. For the cRegulon alignment, we achieved maximum PCC 0.95, averaged PCC 0.79, and median PCC 0.89 (**Fig. S7b**), showing that our cRegulon are highly conserved when we use higher cell clustering resolution. As an example, we showed the TF module of M3 from 18 cell clusters (**Fig. S7c**), which was aligned with M5 of 17 cell clusters (M5 was displayed in our manuscript Figure 6d). We could see the core TF pairs in M5 were also involved in Olig1, Sox8/10, and Nfi family for glia functions, which was the same with M5 of 17 cell clusters.

Then we investigated the influence of lower resolutions. We first set a lower resolution of 0.45 to obtain 16 cell clusters and conducted the same robustness analysis as above. We found that there was also a good one-to-one match between 9 cRegulons from 16 cell clusters and 9 cRegulons from 17 cell clusters (**Fig. S7d**). We observed maximum PCC 0.93, averaged PCC 0.77, and median PCC 0.87 for the aligned cRegulons (**Fig. S7e**). The M1 from 16 cell cluster that aligned with M5 from 17 cell clusters was also marked by Olig1, Sox8/10, and Nfi family (**Fig. S7f**). To evaluate if a much worse cell clustering will influence our model, we continue to lower the resolution to 0.05 to obtain 6 coarse cell clusters. In the cRegulon PCC heatmap, we can see that there is still a good matching between 9 cRegulons from 6 cell clusters and 9 cRegulons from 17 cell clusters (**Fig. S7g**). Their aligned cRegulons had maximum PCC 0.86, averaged PCC 0.60, and median PCC 0.62 (**Fig. S7h**). The M2 from 6 cell cluster that was matched with M5 from 17 cell clusters was still marked by Olig1, Sox8/10, and Nfi family (**Fig. S7i**).

In summary, these analysis with higher, lower, and coarse resolutions showed that cRegulon indeed identifies the basic regulatory units of landscape from the whole dataset, which is robust to cell cluster numbers.

Text S4: cRegulon is robust to the unpaired single cell dataset

Single-cell multi-omics data can be either paired or unpaired, meaning that scRNA-seq and scATAC-seq data are derived from the same cells or different cells, respectively. Unpaired scRNA-seq and scATAC-seq data have the potential to introduce bias into models, as additional alignment between the two omics layers is required. To validate that cRegulon is not sensitive to unpaired data, we conducted an analysis using simulated single-cell data from four cell lines, which originally consisted of paired data used to derive seven cRegulons in our manuscript. We then treated this dataset as unpaired and applied the commonly used Seurat pipeline to identify four clusters within the scRNA-seq and scATAC-seq data, followed by alignment between the two. After alignment, we input the pseudo-bulk data into cRegulon and identified seven cRegulons. To assess whether the TF modules were significantly influenced by the unpaired data, we computed the Pearson correlation coefficients (PCCs) of the X vectors for the seven cRegulons derived from unpaired data compared to those from paired data.

As shown in **Fig. S8**, the cRegulons from unpaired data were highly consistent with those from paired data (Unpaird-M1 and Paired-M6, Unpaird-M2 and Paired-M5, Unpaird-M3 and Paired-M3, Unpaird-M4 and Paired-M2, Unpaird-M5 and Paired-4 Unpaird-M6 and Paired-M7, Unpaird-

M7 and Paired-M1), with an average PCC of 0.78 and a median PCC of 0.81. These consistent results indicate that cRegulon is robust and applicable to unpaired data with minimal sensitivity.

Text S5: cRegulon can be extended to study RE combination

Then we showed the potential advantages of cRegulon to reveal the combination of REs. TFs bind on REs to play their regulatory role on TGs. Since TFs will work as a module, the REs will also potentially work together as combinations by the binding events of TFs. Then we sought to evaluate the ability of cRegulon's TF module to predict RE's combination.

We built gold standard RE combinations through the data from multiplexed CRISPRi experiments. Previously, the epistasis effects among 7 enhancers of MYC in K562 were measured by a large-scale of multiplex CRISPRi experiments7. We used 0.4 of epistasis score as a threshold to define gold standard RE pairs (**Fig. S9a**).

To access the share TF modules of these RE (**Fig. S9b**), wwe first used Homer to scan M TFs' binding affinity on these 7 enhancers, which gave binding matrix $B \in R^{7\times M}$. We directly utilized the 7 TF modules from our cell line study, which included K562. The binding affinity of a TF module on an enhancer was defined as the weighted summation of TF binding affinity and the weights were given by TF module matrix $X \in R^{M\times 7}$, which gave the TF module binding matrix $Y \in R^{7\times 7}$. We normalized the TF modules by columns and normalized the enhancers by rows. Then we used a binding affinity threshold of 0.5 to select this RE's associated TF module. Finally, the RE's combination was predicted by Jaccard similarity of their binding TF modules. Alternately, directly using binding individual TFs and the co-accessibility of REs can also be used to predict RE combinations (**Fig. S9b**).

We used the above-defined gold standard RE combinations to compute the AUROC to evaluate the accuracy of predictions of TF module binding, TF binding, and co-accessibility (**Fig. S9c**). We found that TF binding and co-accessibility only gave AUROC of 0.21 and 0.25, respectively, showing poor accuracy. With the help of TF module, the AUROC was improved to 0.49, much larger than the alternative methods. This experiment showed that TF modules have great potential to reveal more detailed cooperations of regulation, such as RE combinatorial regulation.

Reference

1. Jia YY, Chang YQ, Guo ZK, Li H: **Transcription factor Tbx5 promotes cardiomyogenic differentiation of cardiac fibroblasts treated with 5-azacytidine.** *Journal of Cellular Biochemistry* 2019, **120:**16503-16515.
2. Gilkes DM, Bajpai S, Chaturvedi P, Wirtz D, Semenza GL: **Hypoxia-inducible Factor 1 (HIF-1) Promotes Extracellular Matrix Remodeling under Hypoxic Conditions by Inducing P4HA1, P4HA2, and PLOD2 Expression in Fibroblasts.** *Journal of Biological Chemistry* 2013, **288:**10819-10829.

3. Valenzuela NM: **BCL6B is a Putative Endothelial-Specific Master Regulator.** *Circulation* 2021, **144**.
4. Debnath P, Huirem RS, Dutta P, Palchaudhuri S: **Epithelial-mesenchymal transition and its transcription factors.** *Bioscience Reports* 2022, **42**.
5. Maemura K, Hsieh CM, Jain MK, Fukumoto S, Layne MD, Liu YX, Kourembanas S, Yet SF, Perrella MA, Lee ME: **Generation of a dominant-negative mutant of endothelial PAS domain protein 1 by deletion of a potent C-terminal transactivation domain.** *Journal of Biological Chemistry* 1999, **274:**31565-31570.
6. Rauscher FJ, 3rd, Voulalas PJ, Franza BR, Jr., Curran T: **Fos and Jun bind cooperatively to the AP-1 site: reconstitution in vitro.** *Genes Dev* 1988, **2:**1687-1699.
7. Ransone LJ, Visvader J, Sassonecorsi P, Verma IM: **Fos-Jun Interaction - Mutational Analysis of the Leucine Zipper Domain of Both Proteins.** *Genes & Development* 1989, **3:**770-781.
8. Kouzarides T, Ziff E: **The role of the leucine zipper in the fos-jun interaction.** *Nature* 1988, **336:**646-651.

Supplementary Figures

Fig. S1

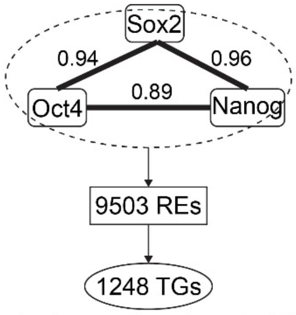

Fig. S1. An example of Sox2, Oct4 and Nanog to combinatorically regulate pluripotency of mESC. The GRN is retrieved from Duren, et al. 2020. These three TFs greatly share the downstream 9503 REs and 1248 TGs. Using connection specificity index (CSI) to measure their combinatorial effect, Sox2 and Oct4 have 0.94, Sox2 and Nanog have 0.96, Oct4 and Nanog have 0.89, showing large combinatorial effects.

Fig. S2

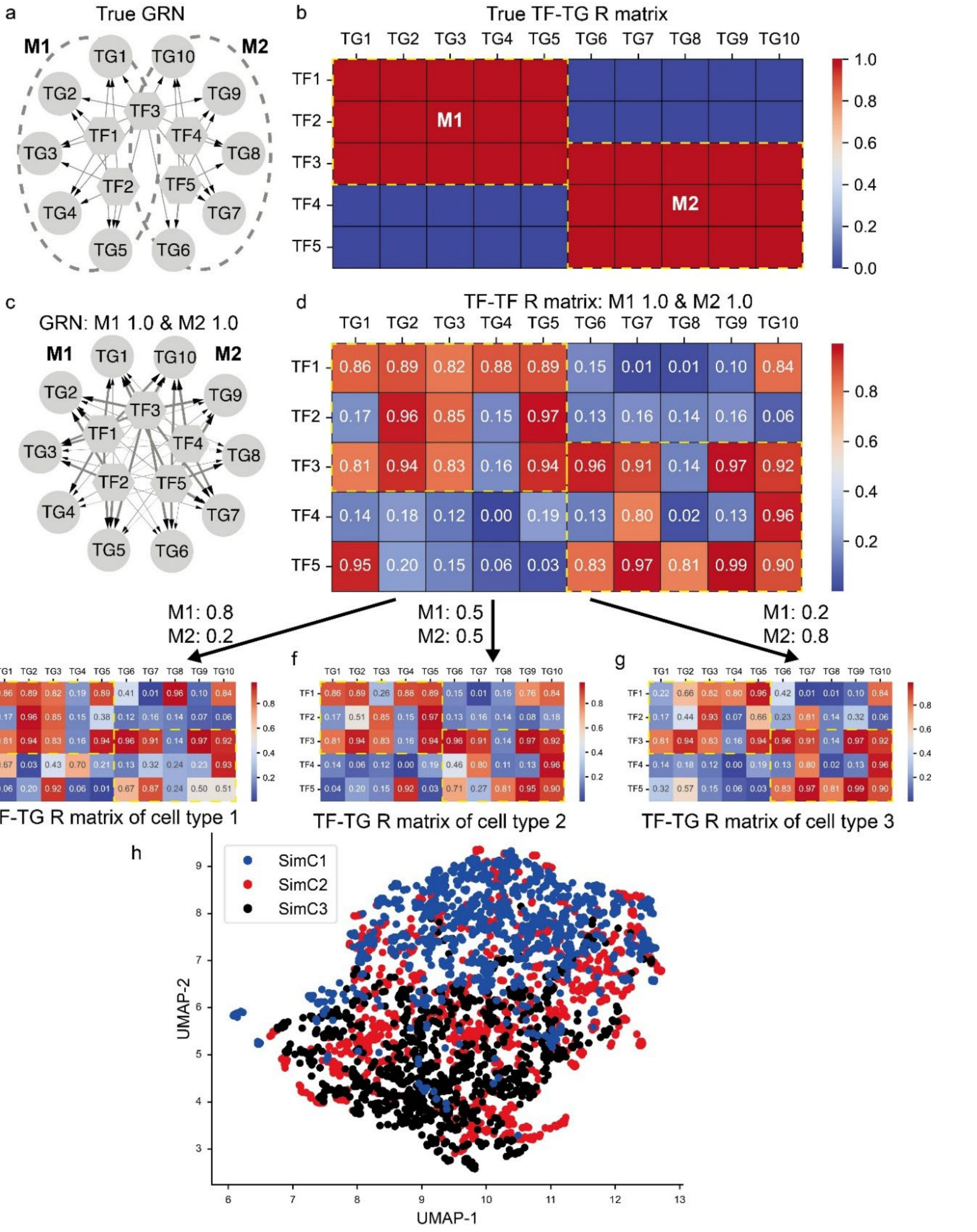

Fig. S2. Process of in-silico simulation. (a) The ideal GRN with two modules. (b) The TF-TG R matrix (adjacent matrix) of the ideal GRN. (c) The simulated GRN with two modules. (d) The adjacent matrix of the simulated GRN. (e) SimC1 with 0.8 M1 and 0.2 M2. M1 is strong, M2 is ambiguous. (f) SimC2 with 0.5 M1 and 0.5 M2. M1 and M2 are clear. (g) SimC3 with 0.2 M1 and 0.8 M2. M1 is ambiguous, M2 is strong. (h) UMAP of simulated scRNA-seq data.

Fig. S3

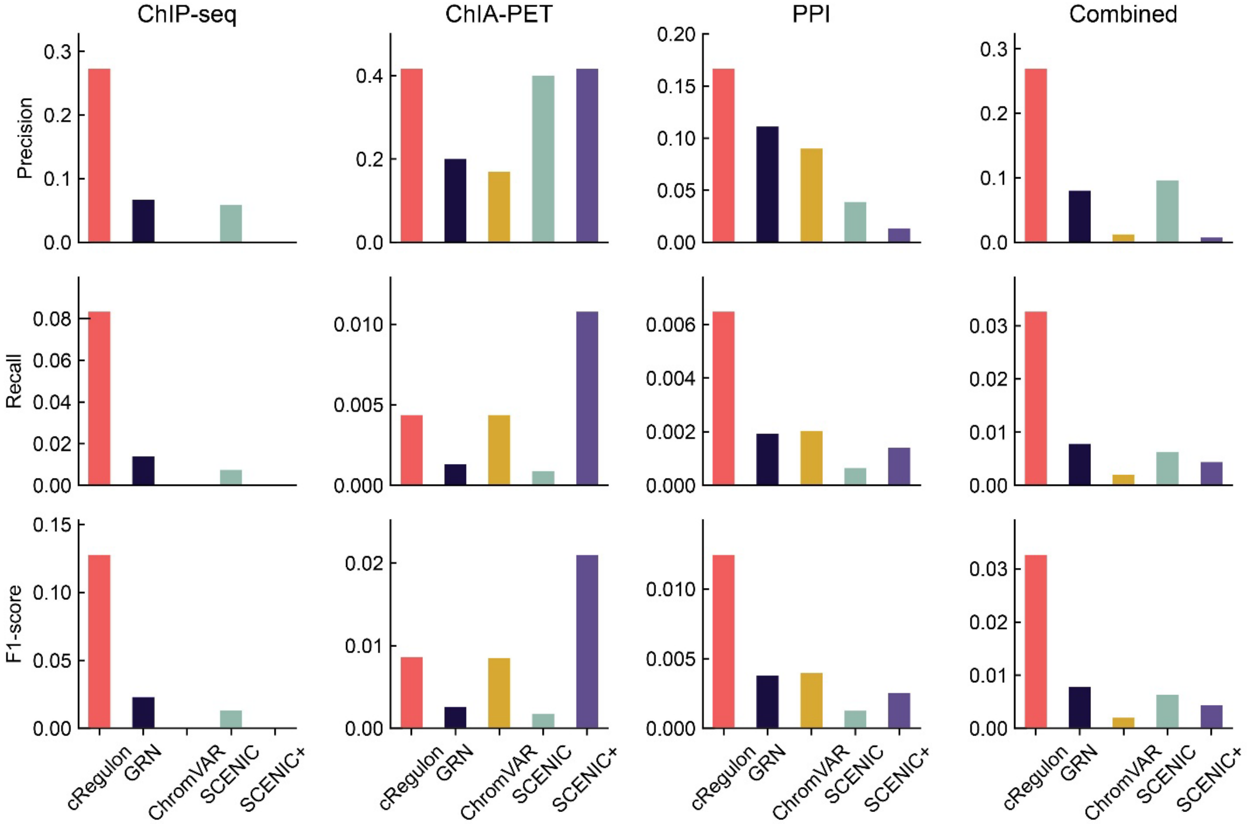

Fig. S3. Comparison of p-value thresholding TF pairs of four baseline methods with precision, recall and F1-score as metrics.

Fig. S4

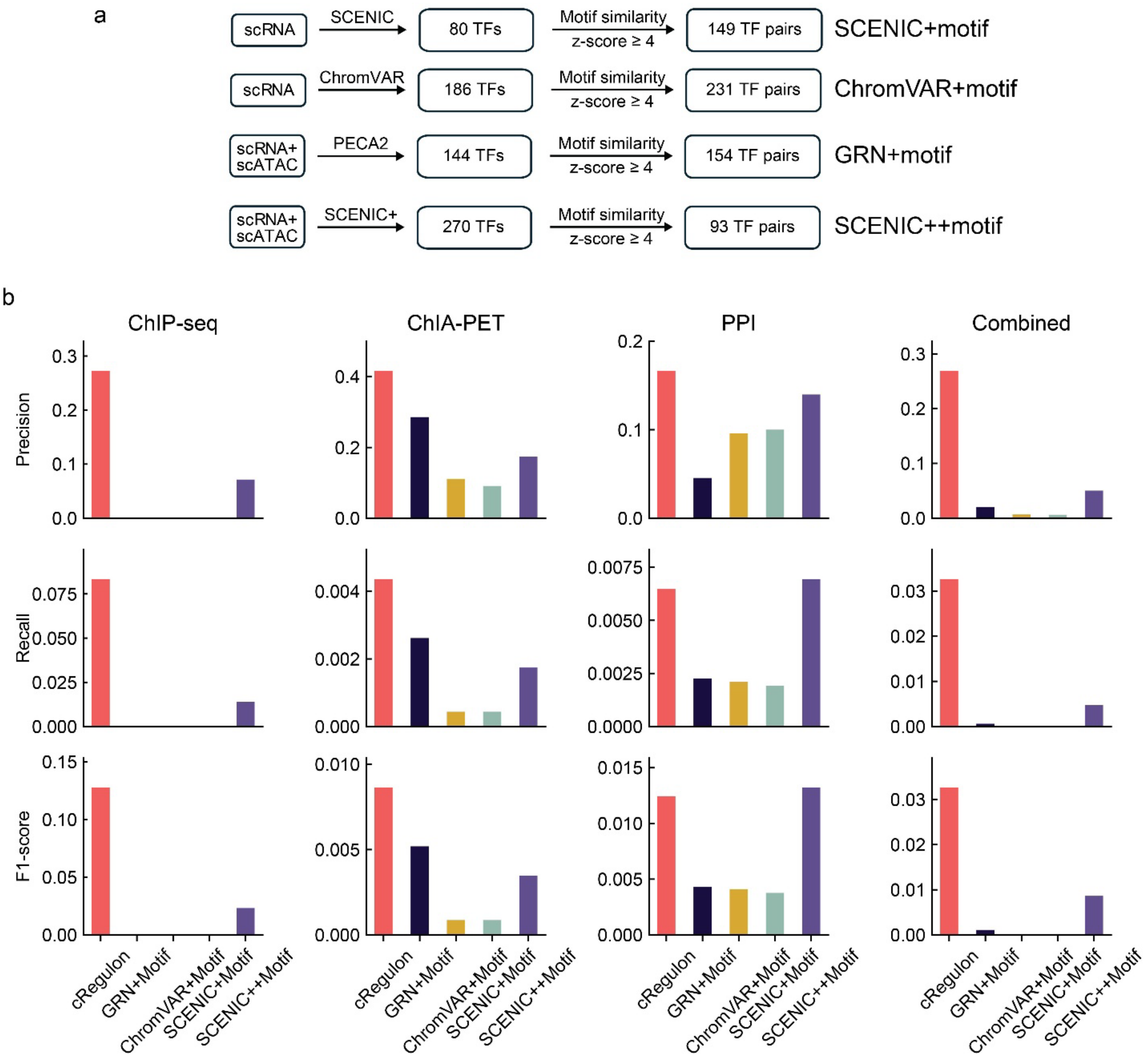

Fig. S4. Comparison with four naive baseline methods. (a) Four methods to identify TF combinations by combining motif similarity with TFs identified by SCENIC, ChromVAR, GRN (PECA2), and SCENIC+ . (b) Comparison of p-value thresholding TF pairs of four naive baseline methods with precision, recall and F1-score as metrics.

Fig. S5

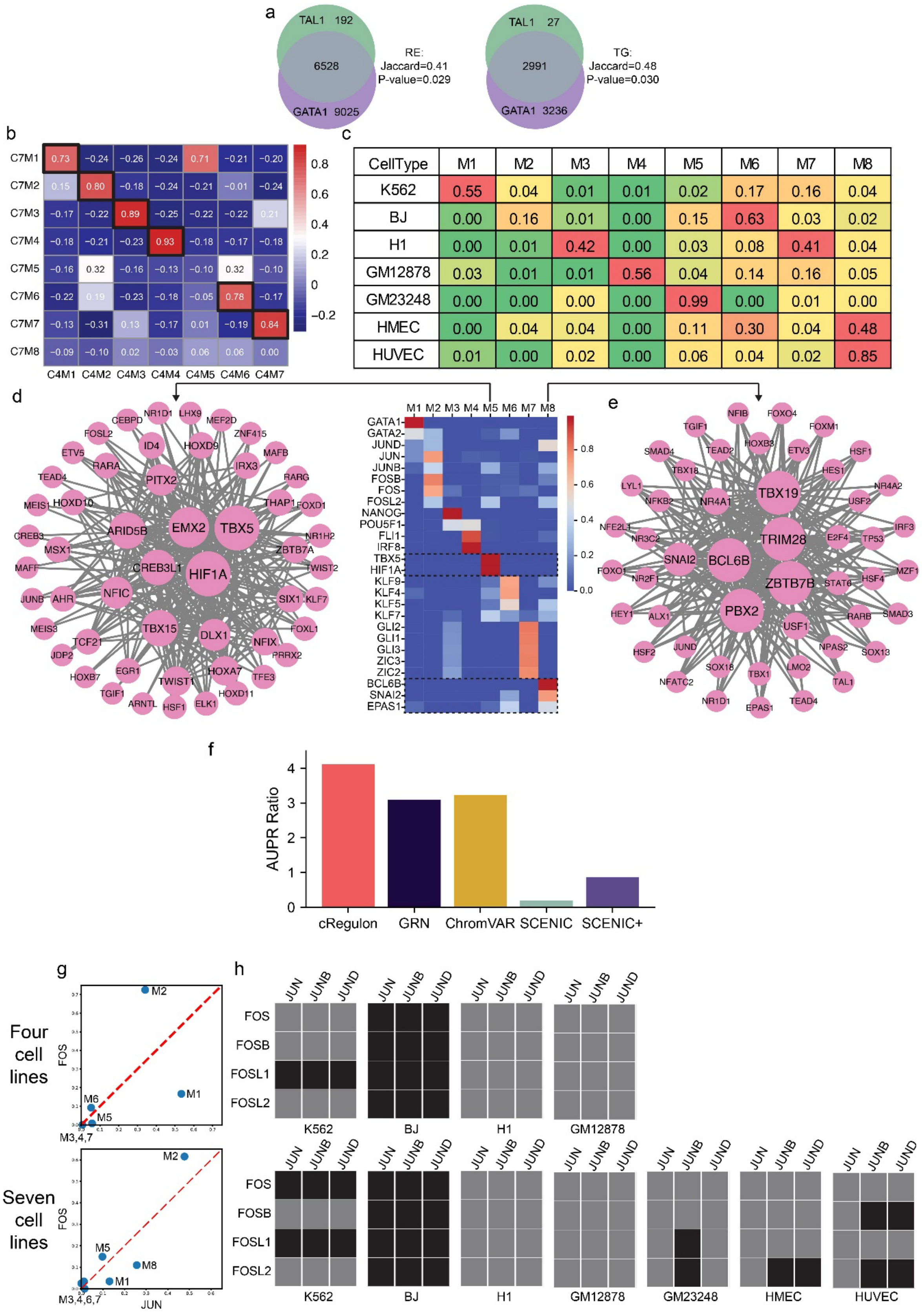
a
TAL1 192
6528
GATA1 9025
RE:
Jaccard=0.41
P-value=0.029
TAL1 27
2991
GATA1 3236
TG:
Jaccard=0.48
P-value=0.030
b
C7M1 0.73 −0.24 −0.26 −0.24 0.71 −0.21 −0.20
C7M2 0.15 0.80 −0.18 −0.24 −0.21 −0.01 −0.24
C7M3 −0.17 −0.22 0.89 −0.25 −0.22 −0.22 0.21
C7M4 −0.18 −0.21 −0.23 0.93 −0.18 −0.17 −0.18
C7M5 −0.16 0.32 −0.16 −0.13 −0.10 0.32 −0.10
C7M6 −0.22 0.19 −0.23 −0.18 −0.05 0.78 −0.17
C7M7 −0.13 −0.31 0.13 −0.17 0.01 −0.19 0.84
C7M8 −0.09 −0.10 0.02 −0.03 0.06 0.06 0.00
C4M1 C4M2 C4M3 C4M4 C4M5 C4M6 C4M7
0.8 0.6 0.4 0.2 0 −0.2
c
CellType M1 M2 M3 M4 M5 M6 M7 M8
K562 0.55 0.04 0.01 0.01 0.02 0.17 0.16 0.04
BJ 0.00 0.16 0.01 0.00 0.15 0.63 0.03 0.02
H1 0.00 0.01 0.42 0.00 0.03 0.08 0.41 0.04
GM12878 0.03 0.01 0.01 0.56 0.04 0.14 0.16 0.05
GM23248 0.00 0.00 0.00 0.00 0.99 0.00 0.01 0.00
HMEC 0.00 0.04 0.04 0.00 0.11 0.30 0.04 0.48
HUVEC 0.01 0.00 0.02 0.00 0.06 0.04 0.02 0.85
d
HIF1A TBX5 EMX2 CREB3L1 ARID5B PITX2 NFIC TBX15 DLX1 ID4 HOXD9
e
TBX19 TRIM28 BCL6B ZBTB7B PBX2 SNAI2
M1 M2 M3 M4 M5 M6 M7 M8
GATA1 GATA2 JUND JUN JUNB FOSB FOS FOSL2 NANOG POU5F1 FLI1 IRF8 TBX5 HIF1A KLF9 KLF4 KLF5 KLF7 GLI2 GLI1 GLI3 ZIC3 ZIC2 BCL6B SNAI2 EPAS1
0.8 0.6 0.4 0.2 0.0
f
AUPR Ratio
cRegulon GRN ChromVAR SCENIC SCENIC+
g
Four cell lines
Seven cell lines
FOS
JUN
M1 M2 M5 M6 M8
M3,4,7
M3,4,6,7
h
JUN JUNB JUND
FOS FOSB FOSL1 FOSL2
K562 BJ H1 GM12878 GM23248 HMEC HUVEC

Fig. S5. Simulation study of effect of more cell types. (a) Overlapping of REs (left) and TGs (right) for GATA1 and TAL1 in K562. (b) Pearson correlation between 8 cRegulons from 7 cell lines (row) and 7 cRegulons from 7 cell lines (column). PCC was computed by corresponding X vectors. (c) Association matrix between 8 cRegulons and 7 cell lines. (d) TF module of M5. (e) TF module of M8. (f) Comparison of TF pair accuracy with four baseline methods on 7-cell-line dataset. (g) Averaged X values of JUN TFs and FOS TFs in 7 cRegulons from 4 cell lines (up) and 8 cRegulons from 7 cell lines (down). (h) The combinatorial effect between JUN and FOS TFs in 4 cell lines (up) and 7 cell lines (down). The black squares indicate non-zero combinatorial effects.

Fig. S6

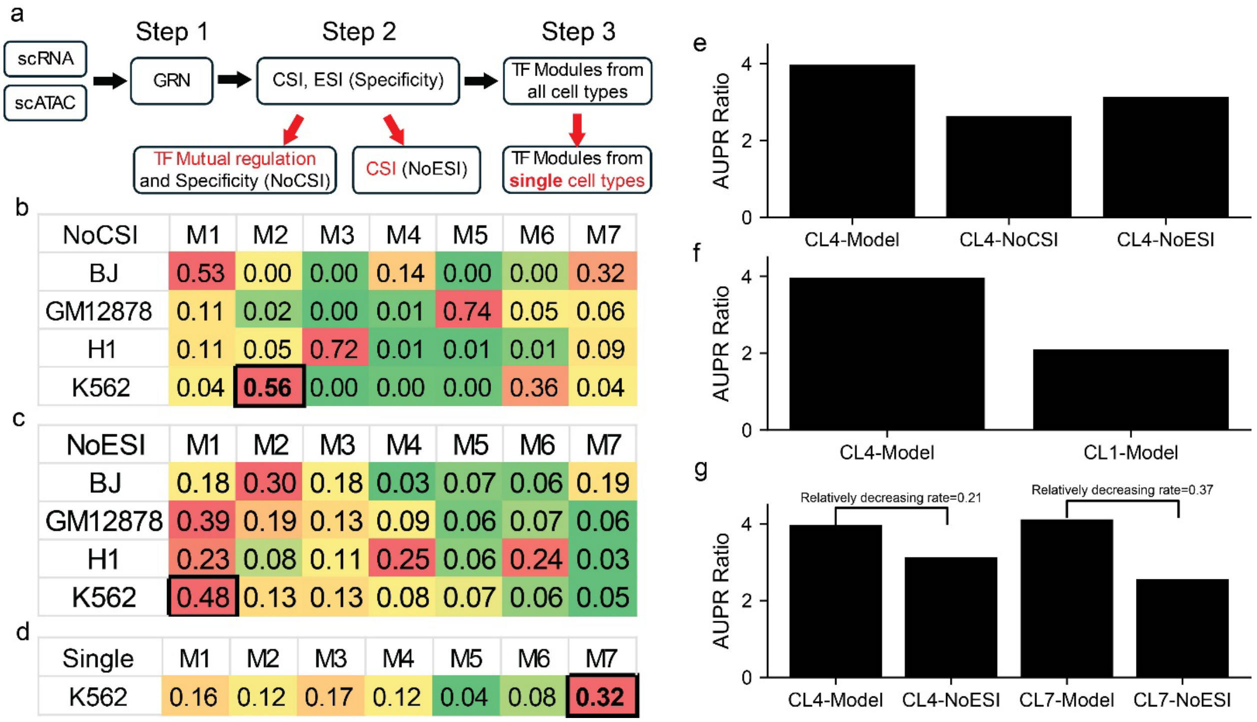

Fig. S6. Ablation study shows the importance of components of cRegulon modeling. (a) Schematic of ablation study. (b) Association matrix of "NoCSI". (c) Association matrix of "NoESI". (d) Association matrix of "Single". (e) Comparison with "NoCSI" and "NoESI" by AUPR ratio of identified K562 TF pairs. (f) Comparison with "Single" by AUPR ratio of identified K562 TF pairs. (g) The effect of "ESI" on data with different levels of heterogeneity, with relatively decreasing rates as metric.

Fig. S7

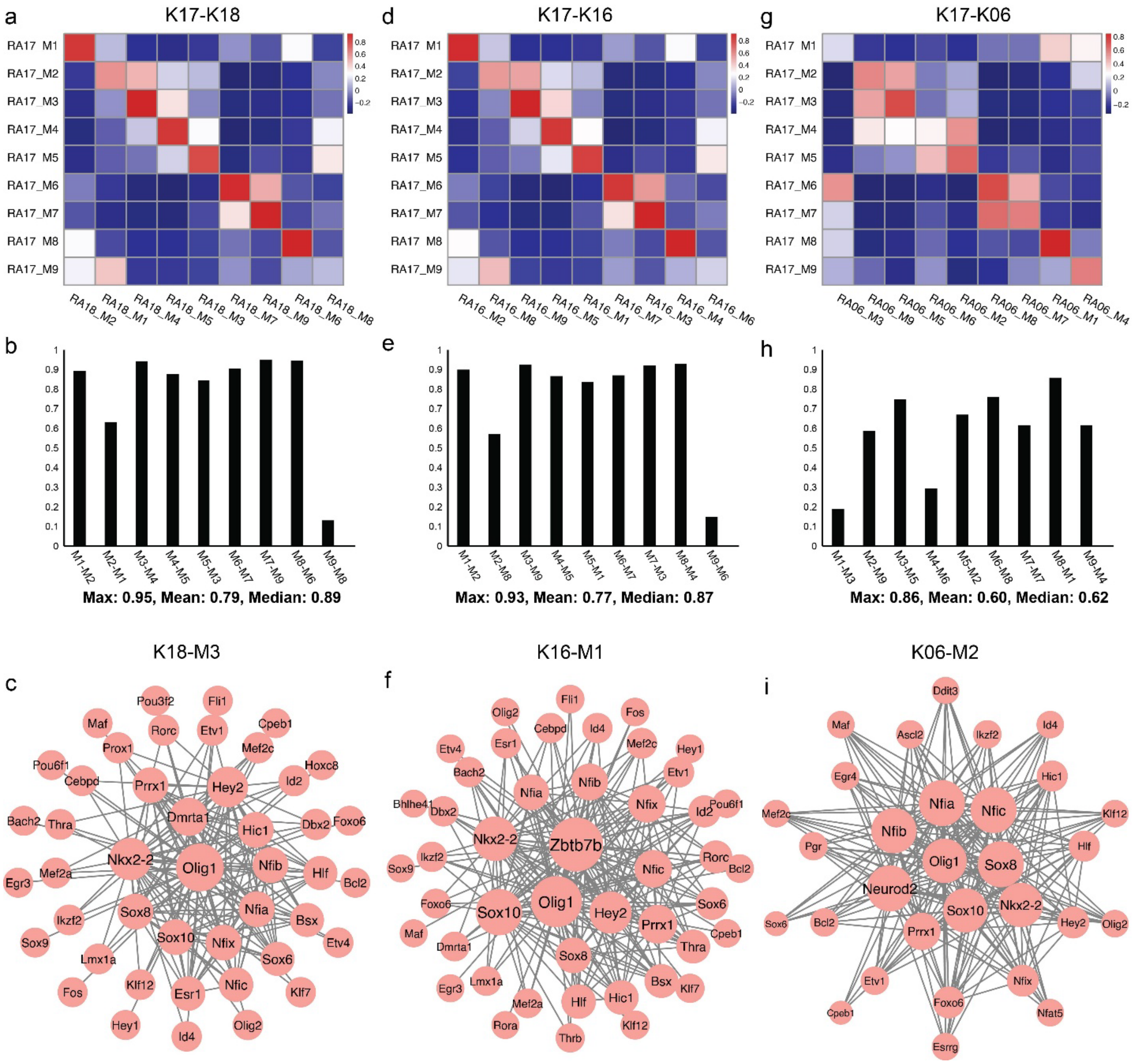

Fig. S7. cRegulon is robust to cell cluster number. (a) Heatmap of Pearson correlation between 9 TF modules from 18 cell clusters and 9 TF modules from 17 cell clusters. (b) The correlation of 9 aligned cRegulons between 18 and 17 cell clusters. (c) The TF module of M3 from 18 cell clusters. (d) Heatmap of Pearson correlation between 9 TF modules from 16 cell clusters and 9 TF modules from 17 cell clusters. (e) The correlation of 9 aligned cRegulons between 16 and 17 cell clusters. (f) The TF module of M1 from 16 cell clusters. (g) Heatmap of Pearson correlation between 9 TF modules from 6 cell clusters and 9 TF modules from 17 cell clusters. (h) The correlation of 9 aligned cRegulons between 6 and 17 cell clusters. (i) The TF module of M2 from 6 cell clusters.

Fig. S8

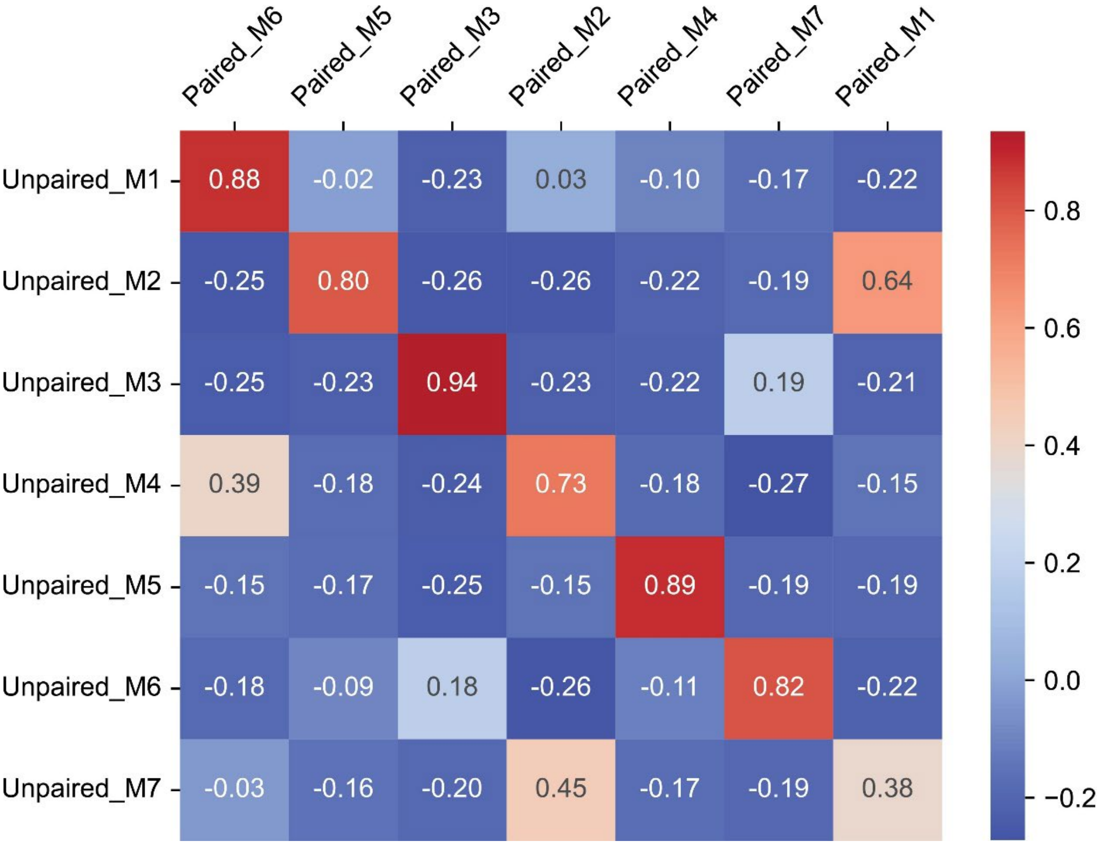

Fig. S8. Pearson correlation of X matrix between cRegulons of unpaired data and paired data. Unpaired_M1 means cRegulon 1 of unpaired data. Paired_M1 means cRegulon 1 of paired data. The other labels are similar.

Fig. S9

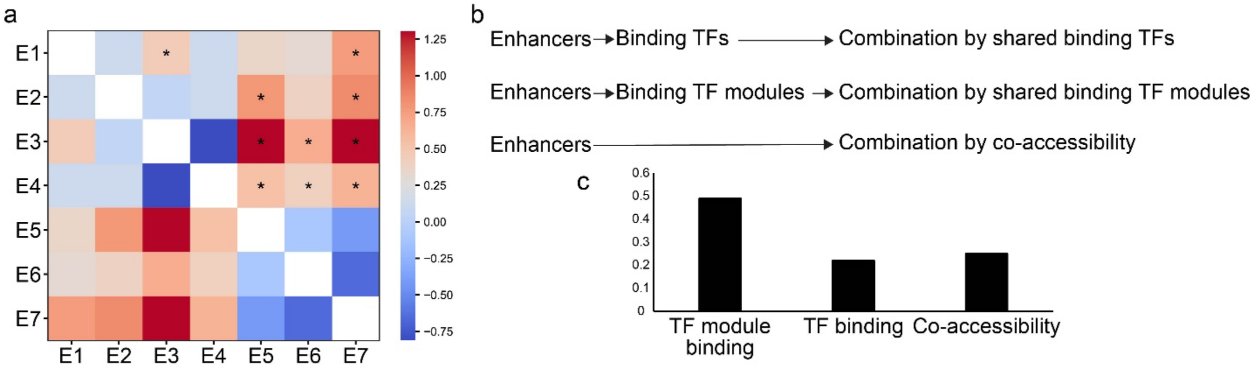

Fig. S9. Extension of cRegulon to study RE combination. (a) The epistasis score among 7 enhancers of MYC in K562. The asterisk indicates gold standard RE pairs with epistasis scores larger than 0.4. (b) The procedures to predict RE combination by TF module binding, TF binding and co-accessibility. (c) AUROC of the predictions of TF module binding, TF binding and co-accessibility.

Fig. S10

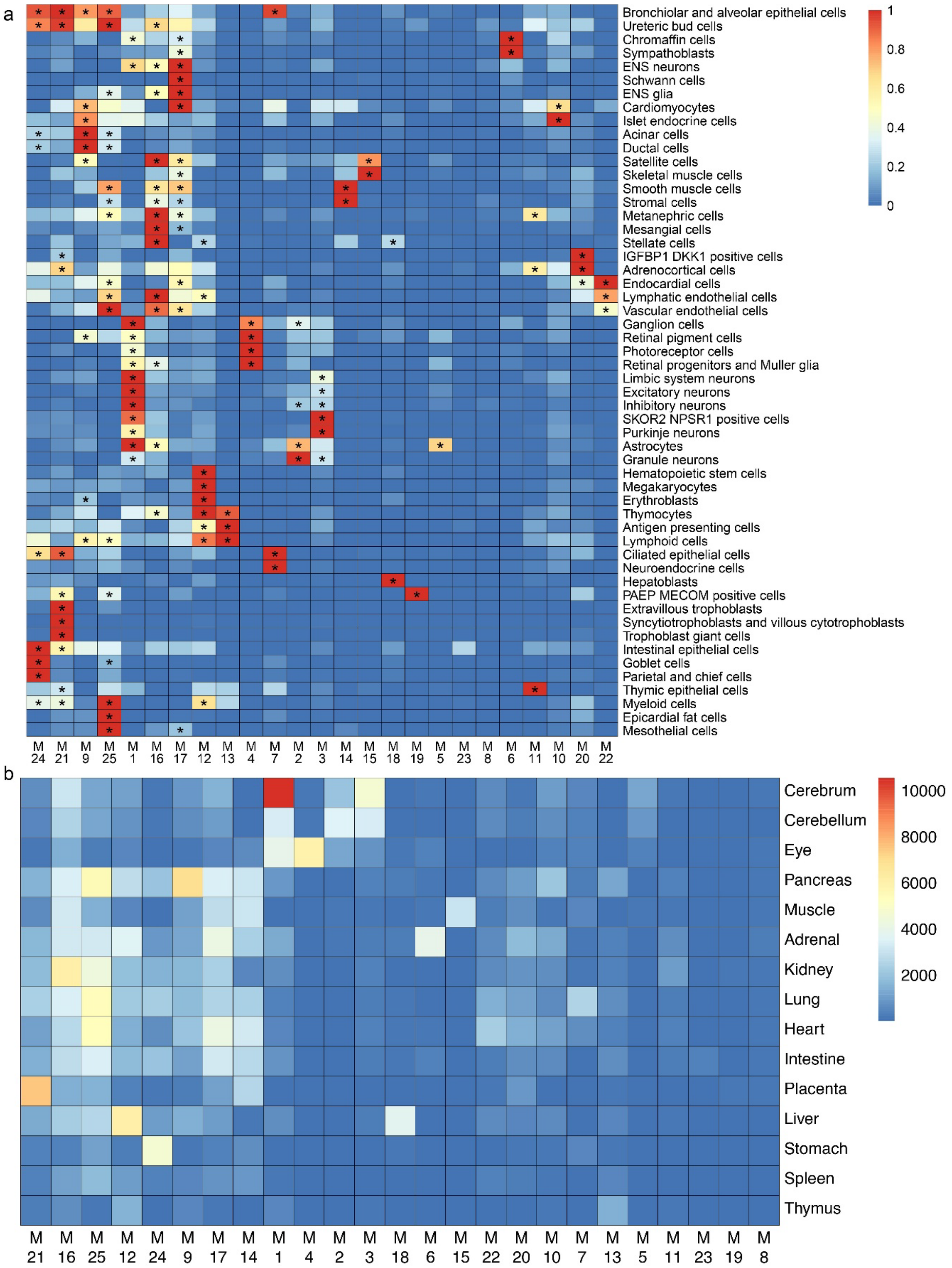

Fig. S10. (a) Heatmap of association strength (A matrix) between 25 cRegulons and 54 cell types. (b) Heatmap of association strength between 25 cRegulons and 15 organs.

Fig. S11

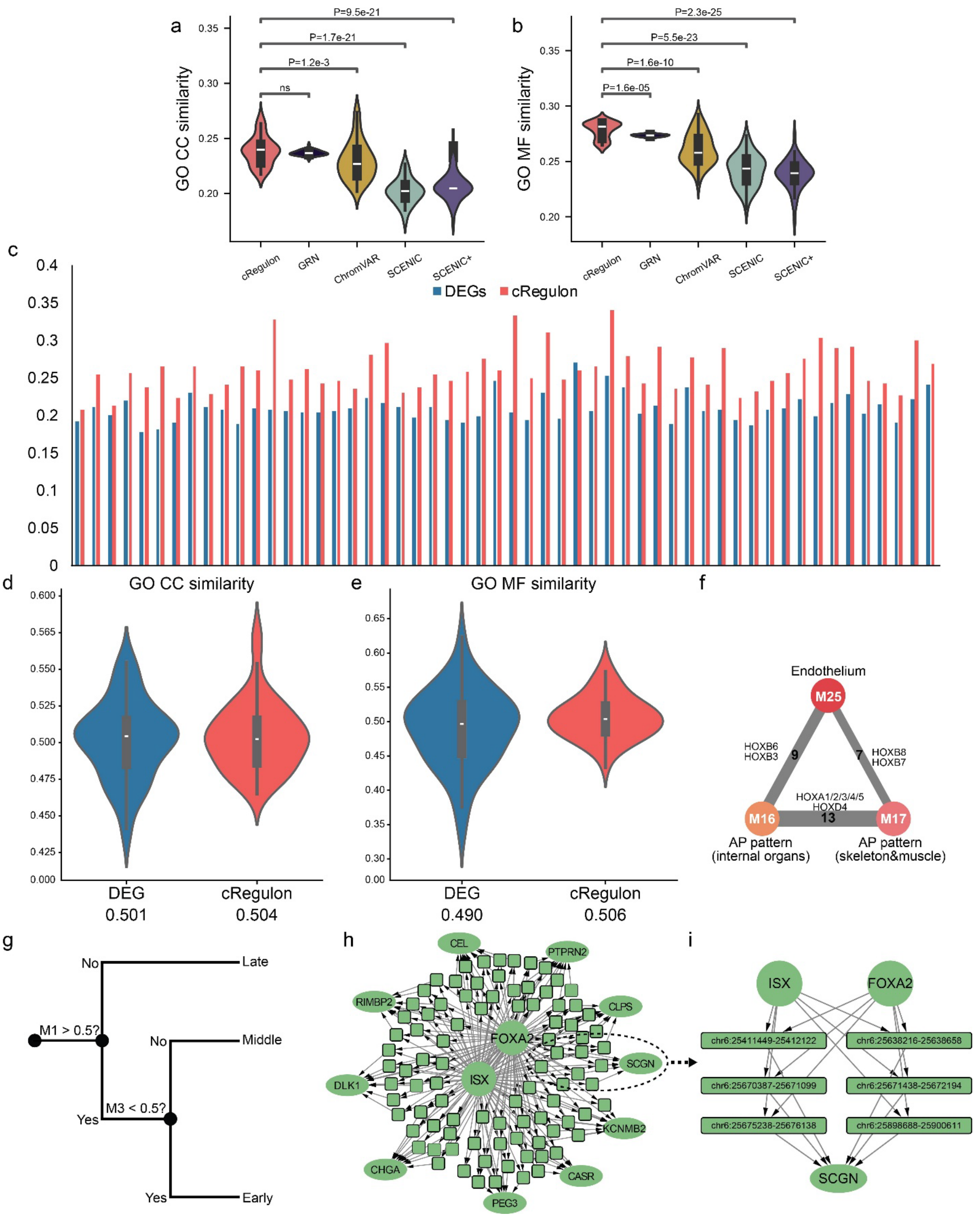

Fig. S11. Comparison of Functional enrichment concentration. (a) The GO CC similarity score of cRegulon TFs, GRN TFs, ChromVAR TFs, SCENIC TFs and SCENIC+ TFs. (b) The GO MF similarity score of cRegulon TFs, GRN TFs, ChromVAR TFs, SCENIC TFs and SCENIC+ TFs. (c) The GO BP similarity score of DEGs and cRegulon genes of

54 cell types. (d) The GO CC similarity score of DEGs and cRegulon genes. (e) The GO MF similarity scores of DEGs and cRegulon genes. (f) The overlapping of 3 cRegulon shows the source of common modules. (g) Schematic for grouping excitatory neurons based on M1 and M3. (h) Combinatorial regulatory network of FOXA2 and ISX on their top 10 TGs. The rectangle with thicker frame indicates shared REs. (i) The local regulation of FOXA2 and ISX on SCGN.

Fig. S12

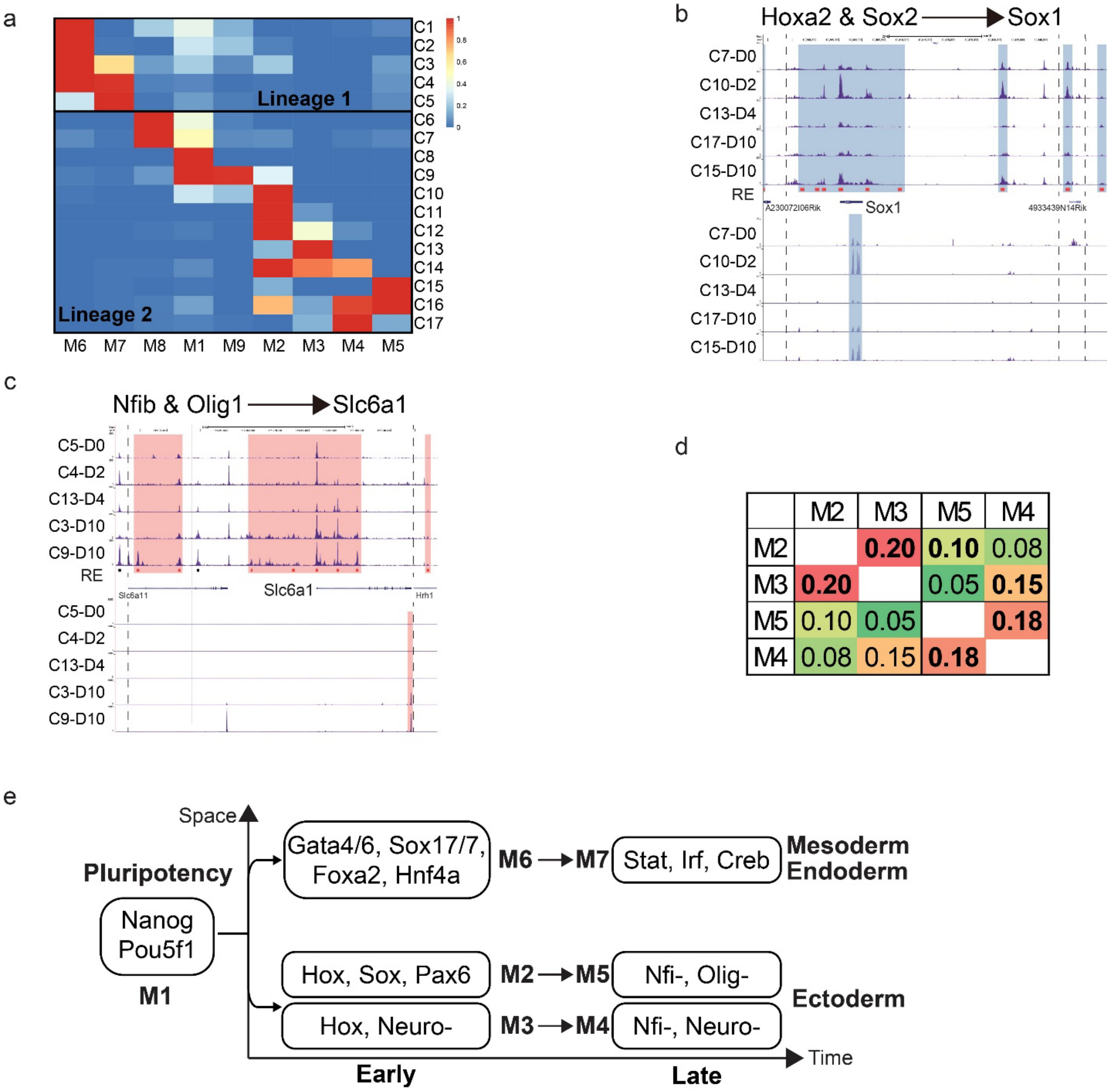

Fig. S12. Detailed combinatorial regulation of RA dataset revealed by cRegulon. (a) Heatmap of association strength (A matrix) between 9 cRegulons and 17 cell clusters of RA data. (b) Dynamical changes of REs and TGs in the combinatorial regulatory network for M2. (c) Dynamical changes of REs and TGs in the combinatorial regulatory network for M5. (d) Heatmap of cRegulon transition scores between two early and two late cRegulons. (e) Schematic summary of cRegulons controlling the landscape of RA-induced mEB differentiation.

Fig. S13

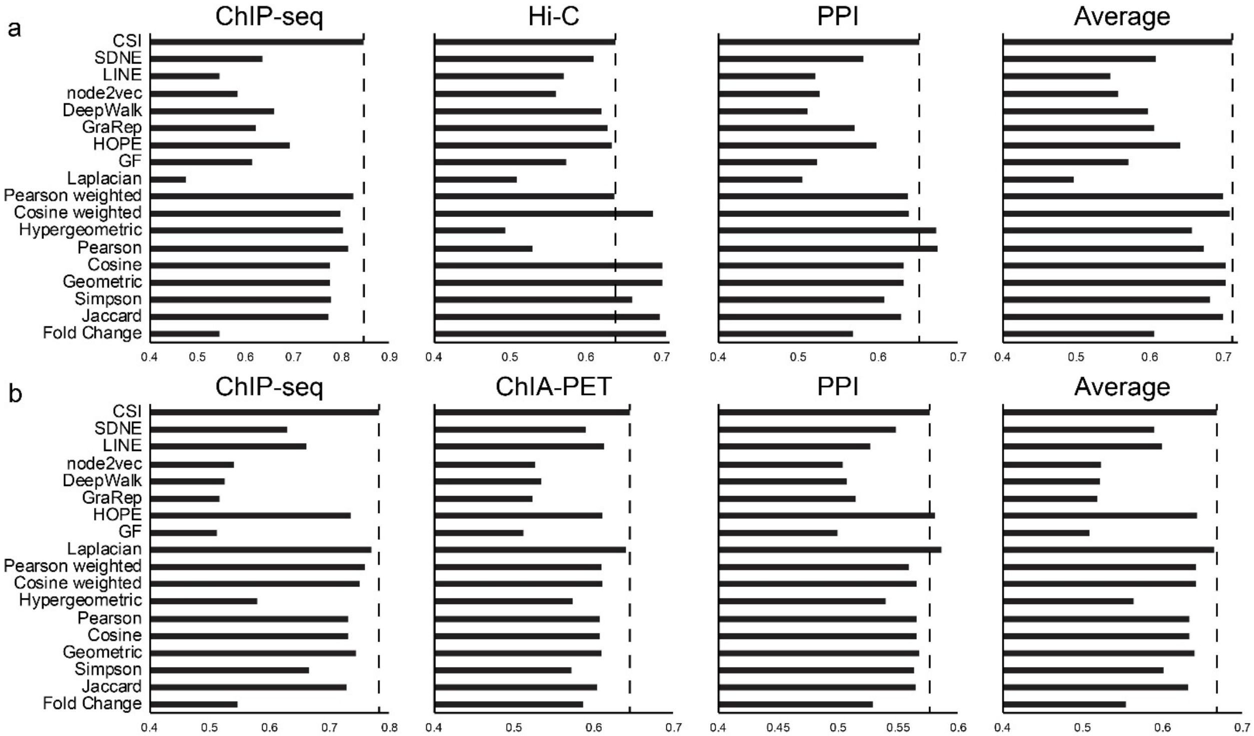

Fig. S13. With gold standard TF pairs from ChIA-PET, ChIP-seq, and PPI data, we compare CSI with other 17 tools for evaluating TF co-regulation effect and find CSI exhibit best performance. (a) Validation of AUROC on HepG2 dataset. (b) Validation of AUROC on K562 dataset.

Fig. S14

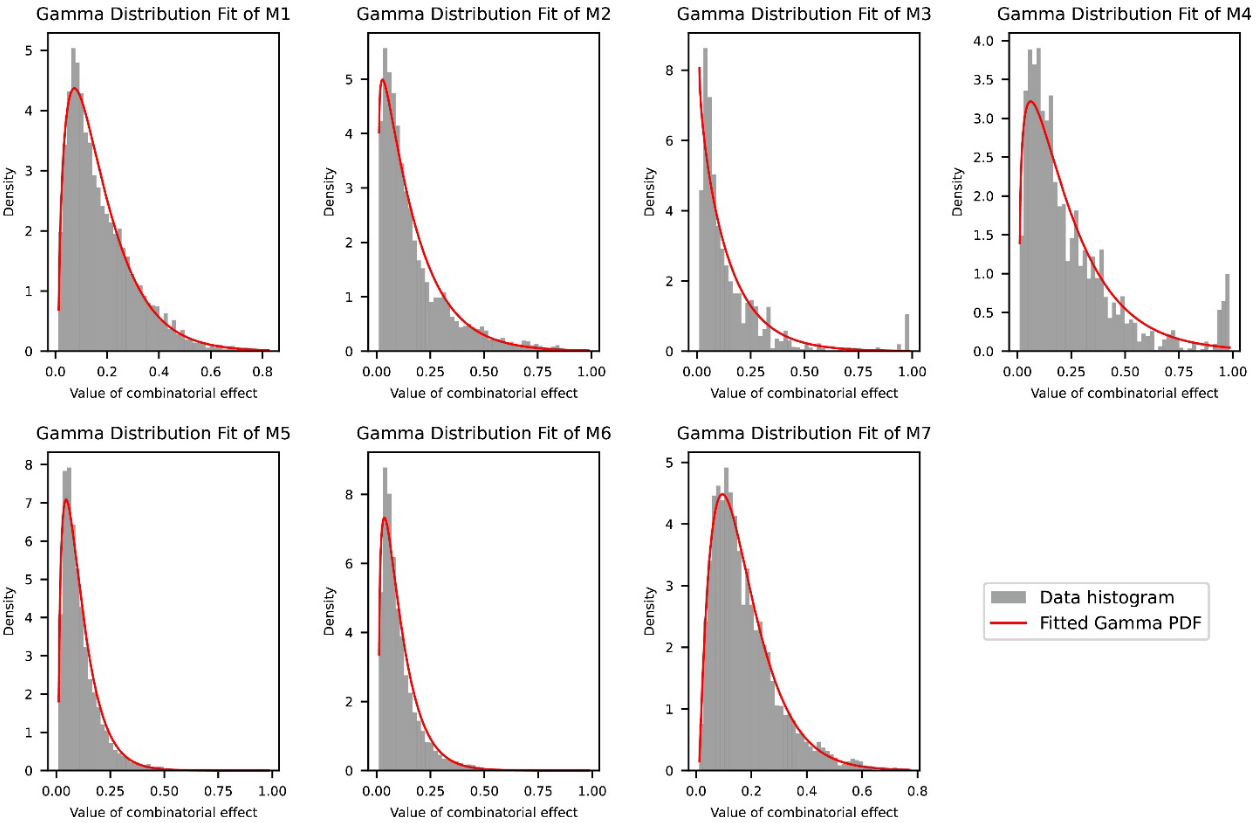

Fig. S14. The empirical distribution of TF pairs' combinatorial effect in 7 cRegulons of 4-cell-line simulation study.

Fig. S15

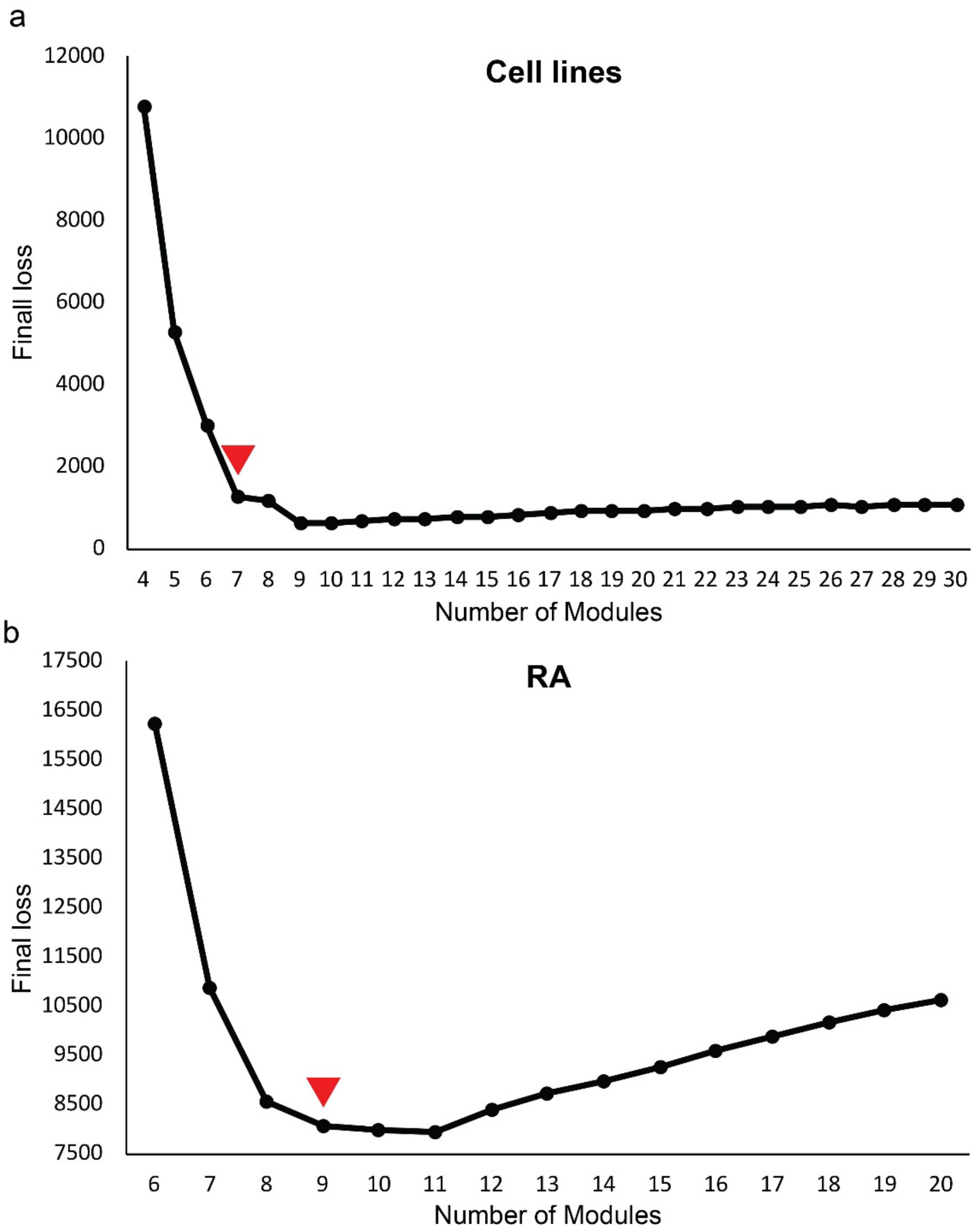

Fig. S15. Choosing optimal number of cRegulons with final loss. (a). Final loss of our model with different settings of TF module number in cell line experiment. The final loss doesn't dramatically decrease after 7. (b) Final loss of our model with different settings of TF module number in RA experiment. The final loss doesn't dramatically decrease after 9.
